# Japanese Society for Cancer of the Colon and Rectum (JSCCR) guidelines 2020 for the Clinical Practice of Hereditary Colorectal Cancer

**DOI:** 10.1007/s10147-021-01881-4

**Published:** 2021-06-29

**Authors:** Naohiro Tomita, Hideyuki Ishida, Kohji Tanakaya, Tatsuro Yamaguchi, Kensuke Kumamoto, Toshiaki Tanaka, Takao Hinoi, Yasuyuki Miyakura, Hirotoshi Hasegawa, Tetsuji Takayama, Hideki Ishikawa, Takeshi Nakajima, Akiko Chino, Hideki Shimodaira, Akira Hirasawa, Yoshiko Nakayama, Shigeki Sekine, Kazuo Tamura, Kiwamu Akagi, Yuko Kawasaki, Hirotoshi Kobayashi, Masami Arai, Michio Itabashi, Yojiro Hashiguchi, Kenichi Sugihara, Japanese Society for Cancer of the Colon, Rectum, Naohiro Tomita, Hideyuki Ishida, Koji Tanakaya, Tatsuro Yamaguchi, Kensuke Kumamoto, Toshiaki Tanaka, Takao Hinoi, Yasuyuki Miyakura, Hirotoshi Hasegawa, Hideki Ishikawa, Takeshi Nakajima, Akiko Chino, Shigeki Sekine, Kazuo Tamura, Kiwamu Akagi, Hirotoshi Kobayashi, Masami Arai, Michio Itabashi, Yojiro Hashiguchi, Kenichi Sugihara

**Affiliations:** 1 Cancer Treatment Center, Toyonaka Municipal Hospital, 4-14-1, Shibahara, Toyonaka, Osaka 560-8565 Japan; 2 Department of Digestive Tract and General Surgery, Saitama Medical Center, Saitama Medical University, Kawagoe, Japan; 3 Department of Surgery, Iwakuni Clinical Center, Iwakuni, Japan; 4 Department of Surgery, Tokyo Metropolitan Cancer and Infectious Diseases Center Komagome Hospital, Tokyo, Japan; 5 Department of Gastroenterological Surgery, Kagawa University, Kagawa, Japan; 6 Department of Surgery, International Catholic Hospital, Tokyo, Japan; 7 Department of Clinical and Molecular Genetics, Hiroshima University Hospital, Hiroshima, Japan; 8 Department of Surgery, Saitama Medical Center, Jichi Medical University, Saitama, Japan; 9 Department of Surgery, Tokyo Dental College Ichikawa General Hospital, Ichikawa, Japan; 10 Department of Gastroenterology and Oncology, Tokushima University Graduate School of Biomedical Sciences, Tokushima, Japan; 11 Department of Molecular-Targeting Prevention, Graduate School of Medical Science, Kyoto Prefectural University of Medicine, Kyoto, Japan; 12 Department of Clinical Genetics, Cancer Institute Hospital of Japanese Foundation for Cancer Research, Tokyo, Japan; 13 Department of Gastroenterology, Cancer Institute Hospital of Japanese Foundation for Cancer Research, Tokyo, Japan; 14 Division of Medical Oncology, Faculty of Medicine, Tohoku Medical and Pharmaceutical University, Sendai, Japan; 15 Department of Clinical Genomic Medicine, Graduate School of Medicine, Dentistry and Pharmaceutical Sciences, Okayama University, Okayama, Japan; 16 Department of Pediatrics, Shinshu University School of Medicine, Matsumoto, Japan; 17 Division of Diagnostic Pathology, National Cancer Center Hospital, Tokyo, Japan; 18 Division of Genetic Medicine, Master of Science, Graduate School of Science and Engineering Research, Kindai University, Higashiosaka, Japan; 19 Department of Molecular Diagnosis and Cancer Prevention, Saitama Cancer Center, Saitama, Japan; 20 Division of Cancer Nursing, College of Nursing Art and Science, University of Hyogo, Akashi, Japan; 21 Department of Surgery, Teikyo University School of Medicine, Mizonokuchi Hospital, Kawasaki, Japan; 22 Clinical Genetics, Juntendo University Graduate School of Medicine, Tokyo, Japan; 23 Department of Surgery, Institute of Gastroenterology, Tokyo Women’s Medical University, Tokyo, Japan; 24 Department of Surgery, Teikyo University School of Medicine, Tokyo, Japan; 25 Tokyo Medical and Dental University, Tokyo, Japan

**Keywords:** Hereditary colorectal cancer, Guidelines, Familial adenomatous polyposis, Lynch syndrome

## Abstract

Hereditary colorectal cancer (HCRC) accounts for < 5% of all colorectal cancer cases. Some of the unique characteristics commonly encountered in HCRC cases include early age of onset, synchronous/metachronous cancer occurrence, and multiple cancers in other organs. These characteristics necessitate different management approaches, including diagnosis, treatment or surveillance, from sporadic colorectal cancer management. There are two representative HCRC, named familial adenomatous polyposis and Lynch syndrome. Other than these two HCRC syndromes, related disorders have also been reported. Several guidelines for hereditary disorders have already been published worldwide. In Japan, the first guideline for HCRC was prepared by the Japanese Society for Cancer of the Colon and Rectum (JSCCR), published in 2012 and revised in 2016. This revised version of the guideline was immediately translated into English and published in 2017. Since then, several new findings and novel disease concepts related to HCRC have been discovered. The currently diagnosed HCRC rate in daily clinical practice is relatively low; however, this is predicted to increase in the era of cancer genomic medicine, with the advancement of cancer multi-gene panel testing or whole genome testing, among others. Under these circumstances, the JSCCR guidelines 2020 for HCRC were prepared by consensus among members of the JSCCR HCRC Guideline Committee, based on a careful review of the evidence retrieved from literature searches, and considering the medical health insurance system and actual clinical practice settings in Japan. Herein, we present the English version of the JSCCR guidelines 2020 for HCRC.

## Introduction

1. Guideline objectives

The number of patients with colorectal cancer has been increasing in Japan, and social awareness is high since it is one of the most common types of cancer. Most colorectal cancers are thought to arise from the accumulation of gene variants in the colonic mucosa and adenomas (sporadic colorectal cancers) due to the effects of lifestyle, environmental factors, and aging. Between 20 and 30% of all colorectal cancers frequently develop in relatives (familial clustering) and are therefore sometimes called familial colorectal cancers. The causative gene in approximately < 5% of colorectal cancers, regardless of familial clustering, has been identified, which is collectively referred to as hereditary colorectal cancer. Hereditary colorectal cancer tends to be complicated by juvenile onset, synchronous/metachronous carcinogenesis, and multiple cancers of other organs, and it is necessary to take measures that are different from those for sporadic colorectal cancer. However, the general clinician’s awareness of hereditary colorectal cancer is not always high.

Familial adenomatous polyposis (FAP) and Lynch syndrome are representative diseases of hereditary colorectal cancer. FAP is often diagnosed because100 or more adenomas usually originate in the colonic mucosa. Meanwhile, Lynch syndrome is the most common disease among hereditary colorectal cancers but is relatively poorly characterized clinically and likely to be missed in daily clinical practice. Lynch syndrome was also previously called hereditary nonpolyposis colorectal cancer (HNPCC), and its disease concepts and diagnostic criteria have undergone a transition with the history of research, which may be confusing in clinical practice.

Within these circumstances, the Japanese Society for Cancer of the Colon and Rectum guidelines 2020 for the Clinical Practice of Hereditary Colorectal Cancer (“JSCCR guidelines 2020 for HCRC”, henceforth referred to as “these guidelines”) were developed for the following four purposes: (1) to deepen the understanding of the concept of hereditary colorectal cancer, (2) to provide guidance on management strategies, including diagnosis and surveillance, for hereditary colorectal cancer, (3) to emphasize the importance of the need to consider the psychosocial burden caused by hereditary diseases in patients and their families (relatives) and their need for support, and (4) to enhance mutual understanding between healthcare professionals and patients by making these guidelines available to the public.

Additionally, it can be thought that summarizing Lynch syndrome as the “JSCCR guidelines for HCRC” may be inappropriate due to the diversity of developing tumors. Given the history to date of the creation of these guidelines in this regard, this question will be left for future examination and revision.

2. How to use these guidelines

These guidelines can be used as a tool for the treatment of hereditary colorectal cancer practice in clinical practice. Specifically, they can be used in the diagnosis, treatment, and surveillance of individual patients or informed consent setting for patients and families. Although the JSCCR is responsible for the content of these guidelines, the responsibility for the individual clinical results should be attributed to the direct practitioner, and the JSCCR and Guideline Committee are not responsible.

3. Users

The users of these guidelines are mainly physicians and healthcare professionals working in the practice of FAP, Lynch syndrome, and related disorders.

4. How to develop these guidelines
1) Circumstances of guideline development

The JSCCR planned to develop the “JSCCR guidelines for the Clinical Practice of Hereditary Colorectal Cancer” as a project of the Familial Colorectal Cancer Committee and published the “JSCCR guidelines for HCRC” in July 2012. Subsequently, several new findings and clinical guidelines, particularly those on Lynch syndrome, were published from overseas. In addition, the Familial Colorectal Cancer Committee analyzed data from “A Retrospective Multicenter Study of Familial Adenomatous Polyposis” and “Registration and Genetic Analysis of HNPCC (Secondary Study),” which were studies conducted by the JSCCR, and obtained new findings. Clinical genetics departments have been established under these circumstances, mainly in specialized institutions, and hereditary tumors have increasingly become an issue of social concern in Japan. Based on the above, the “JSCCR guidelines 2016 for HCRC” was published in November 2016. Afterward, considering the changes in the medical environment, such as the clinical implementation of cancer genome medicine and approval of immune checkpoint inhibitors, the revision of the “JSCCR guidelines 2016 for HCRC” was initiated in January 2019. A draft revision was prepared after several discussions, and public hearing was held in the 92nd annual meeting of the JSCCR in January 2020, after which the revised points were published on the website of the JSCCR to collect public comments. Further revisions were made in reference to these opinions, and this was submitted to the Guideline Evaluation Committee. In turn, further revisions were made in reference to the opinions of this Committee, and the “JSCCR guidelines 2020 for HCRC” (these guidelines) was published in July 2020.

We attempted to develop these guidelines in accordance with the concept of evidence-based medicine. However, the incidence of hereditary colorectal cancer is relatively low, and it is difficult to design high-evidence-level studies. In view of this difficulty in obtaining sufficient evidence, the guidelines have been developed by consensus among members of the JSCCR, based on information obtained from literature searches and considering the medical health insurance system and actual clinical practice situation in Japan. Moreover, considering the special characteristics of hereditary colorectal cancer, members of the Japanese Society for Hereditary Tumors and patient/family associations also participated in the Guideline Development Committee. The Guideline Development Committee comprised specialists in internal medicine, surgery, gynecology, pediatrics, pathology, genetic diagnosis, genetic counseling, and nursing, as well as representatives of patient/family associations.

2) Principles behind guideline development

These guidelines present evidence for each management strategy to allow a clearer understanding of the management strategies, including the diagnosis, treatment, and surveillance of hereditary colorectal cancer; however, the technical aspects of each treatment method have not been discussed.

3) Description method

An outline and treatment of hereditary colorectal cancer are initially shown. FAP and Lynch syndrome, which have relatively high incidence rates among cases of hereditary colorectal cancer, were selected. The outline, diagnosis, treatment, and surveillance of these diseases were described with the abundant use of flowcharts and figures or tables. Easy-to-understand explanations were added when possible as side notes, given the special characteristics of hereditary colorectal cancer, and to deepen the correct understanding of disease characteristics and terminology. Additionally, with the consensus of the Guideline Development Committee, issues with room for discussion were raised as clinical questions (CQs) and accompanied by a recommendation and explanation.

Attempts were made to use clear and nonambiguous expressions in the CQs. The ease of understanding and avoiding insufficiently long texts in the CQ explanations were emphasized. Descriptions of specific figures and values in the research results when referring to a large number of clinical trials were abbreviated as appropriate. The study design on which the recommendations were to be determined was specified whenever possible, including meta-analyses, randomized controlled trials, and observational studies.

Data on FAP and Lynch syndrome obtained in the JSCCR multicenter study were published as documents. Methods for writing and reading pedigrees needed to understand hereditary tumors, description methods of genome variants, and patient support information are also presented as appendices.

4) CQ evidence level and recommendations

Each recommendation in response to a CQ is accompanied, as much as possible, by classifications of the evidence and recommendation categories, based on a consensus reached among members of this Guideline Development Committee.

### Evidence levels

We comprehensively collected the literature on CQs and grouped the evidence presented by individual papers with respect to critical outcomes included in the CQs by study design. Then, as was performed in the JSCCR guidelines 2019 for the treatment of colorectal cancer [1], we assessed the literature-level body of evidence according to the Grading of Recommendations Assessment, Development and Evaluation (GRADE) system [2] and finally determined the evidence level for the CQs. The evidence level was described at four levels: “A: there is strong confidence in the estimated values of the effect”; “B: there is moderate confidence in the estimated values of the effect”; “C: there is limited confidence in the estimated values of the effect”; and “D: there is little confidence in the estimated values of the effect” (Table 1).

**Table 1:** Definition of the evidence level for CQs

Evidence level A (high)	There is strong confidence in the estimated values of the effect
Evidence level B (moderate)	There is moderate confidence in the estimated values of the effect
	The true effect is roughly close to the effect estimate, but it may also differ substantially
Evidence level C (low)	There is limited confidence in the estimated values of the effect
	The true effect may differ substantially from the estimated value of the effect
	There is little confidence in the estimated value of the effect
Evidence level D (very low)	There is little confidence in the estimated value of the effect
	The true effect is likely to differ substantially from the estimated value of the effect

### Strength of recommendation

Draft recommendations were developed based on the outcomes and evidence levels generated by the abovementioned tasks and assessed at a consensus meeting by the Guideline Development Committee members. In the CQ text, the determined recommendations were expressed directly, and diverse expressions were eliminated. The strength of the draft recommendations was determined by voting according to the GRADE Grid method 2), which were assessed according to the following four items: ① certainty of evidence, ② patients’ preferences, ③ benefits and harms, and ④ costs. The strength of the recommendation was described as Strong “For” intervention, A Weak “For” intervention, Weak “Against” intervention, Strong “Against” intervention and ‘Not graded” (Table 2).

**Table 2:** Strength of CQ recommendations

Recommendations	
1 (Strong recommendation)	Strong “For” an intervention
	Strong “Against” an intervention
2 (Weak recommendation)	Weak “For” an intervention
	Weak “Against” an intervention

Representatives of the patient/family association also participated in the CQ voting after freely stating their opinions in the meeting.

[Voting method]

1. Select one of the following five options and vote

- ① Strong “For” intervention
- ② Weak “For” intervention
- ③Weak “Against” intervention
- ④Strong “Against” intervention
- ⑤ Not graded

2. If > 70% or more of the total votes agreed on either ①–⑤ in the first vote, they were determined as such. This condition did not apply:

- If ① + ② exceeds 50% and ③ + ④ is 20% or lower, “weakly recommend to perform.”
- If ③ + ④ exceeds 50% and ① + ② is 20% or less, “weakly recommend not to perform.”

3. If neither of the two conditions were met in the first voting, a second discussion was held under the category of “no consensus was reached” while taking into account the medical circumstances in Japan and disclosing the voting results, and a second voting was held.
4. If no consensus was reached in the second voting, “Not graded” was selected.

Representatives of the patient/family association also participated in the CQ voting after freely stating their opinions in the meeting.

5. Literature search.

A search equation was created for each CQ to collect the most recent literature to add to the previous edition of the adopted literature. PubMed and Ichushi-Web were used as search databases. English-and Japanese-language literatures from September 2015 to February 2019 were systematically searched. Articles were selected from a list of 28,258 abstracted articles (FAP, 1,447 in Japanese and 8,537 in English; Lynch syndrome, 1,271 in Japanese and 17,003 in English) in conjunction with the literature up to the previous edition, and the full text was critically examined after adding articles for manual searches. Important literature published in March 2019 or later was carefully examined before adoption.

## Chapter I. Outlines of hereditary colorectal cancer

### Basic items

- Hereditary colorectal cancer accounts for approximately 5% of all colorectal cancers. Approximately 30% of patients with colorectal cancer are considered genetically predisposed.
- Representative hereditary colorectal cancers for which causative genes have been identified include familial adenomatous polyposis (FAP) and Lynch syndrome. The risk of developing colorectal cancer and type and frequency of concomitant tumors vary depending on the causative gene.
- The onset of colorectal cancers with autosomal dominant inheritance (e.g., FAP, Lynch syndrome) is thought to occur due to a pathogenic germline variant of the causative gene (Side Memo 1: Variant, germline and somatic variants), wherein changes that cause a loss of function in the allele on the opposite side can be acquired as two hits in the epithelial cells of the large intestine, subsequently becoming cancerous.

#### Comments

##### [Definition]

- Colorectal cancer, in which a pathogenic variant of the causative gene has been detected in the germline, is defined as hereditary colorectal cancer regardless of familial clustering. Representative diseases of hereditary colorectal cancer are FAP and Lynch syndrome.
- Some familially clustered colorectal cancers do not have pathogenic variants of the causative genes (Chapter III 2-2: Familial colorectal cancer type X).

##### [Incidence]

**Fig. 1:**
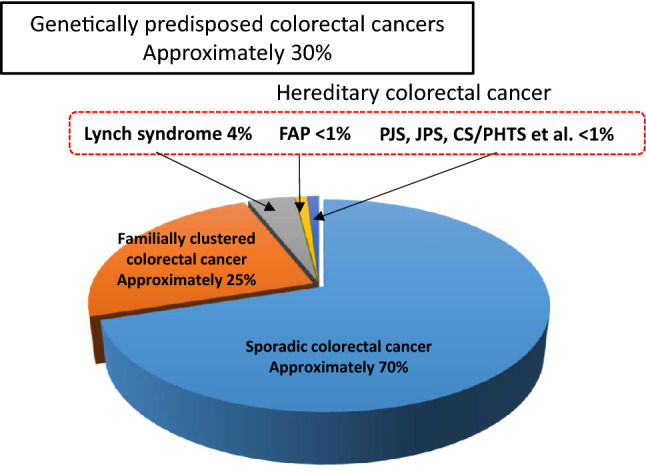
Percentage of genetically predisposed colorectal cancers among all colorectal cancers. PJS Peutz–Jeghers syndrome, JPS juvenile polyposis syndrome, CS/PHTS Cowden syndrome/PTEN hamartoma tumor syndrome

Approximately 30% [3, 4] of all colorectal cancers are considered genetically predisposed colorectal cancers (Fig. 1). Hereditary colorectal cancer accounts for approximately 5% [5] of all colorectal cancers.

- Western studies have estimated that the incidence of Lynch syndrome in all colorectal cancers was between 2 and 4% [6, 7].
- Results of genetic testing (Side Memo 1: Genetic testing) from recent microsatellite instability (MSI) tests for all colorectal cancers or universal screening of mismatch repair protein by immunohistochemistry (IHC) (Chapter III 2-1: Diagnostic flow) reported incidence rates of 2.4–3.7% [8, 9] in Western countries and < 1% [10, 11] in Japan.
- Patients with FAP are estimated to be < 1% [12] of all patients with colorectal cancer, but the exact frequency is unknown.

##### [Major diseases]

- Among the representative diseases of hereditary colorectal cancers (Table 3), FAP, polymerase-proofreading-associated polyposis (PPAP), Lynch syndrome, Peutz–Jeghers syndrome (PJS), juvenile polyposis syndrome (JPS), Cowden syndrome (CS)/PTEN hamartoma tumor syndrome (PHTS), and Li–Fraumeni syndrome (LFS) are the autosomal dominant modes of inheritance. *MUTYH*-associated polyposis (MAP), *MSH3*-associated polyposis, and *NTHL1*-associated polyposis have autosomal recessive modes of inheritance.
- With the exception of FAP, the number of polyps in the large intestine is roughly between 10 and 100. There are often only a few colonic polyps in Lynch syndrome and LFS. The number of colorectal polyps in PJS, JPS, and CS/PHTS ranges from 0 to several dozens, and the histology of the polyps is a hamartoma, with a specific morphology for each disorder.
- The risk of developing colorectal cancer varies by disease, but FAP has nearly 100% penetrance (Side Memo 1: Penetrance). Lynch syndrome has a variable risk of developing colorectal cancer, which depends on the causative mismatch repair genes [13], and women develop endometrial cancer at the same frequency as colorectal cancer [14]. Cross-sectional collaboration between clinical departments is also important because tumors at various sites are known to develop as Lynch syndrome-associated tumors.
- Hereditary colorectal cancers, except Lynch syndrome and LFS, often result in the development of polyps in the stomach and duodenum other than colorectal polyposis.

**Table 3:** Genetic and clinical characteristics of representative hereditary colorectal cancers

Disorder	Inheritance pattern	Causative gene	Frequency	Number of colorectal polyps	Histology of the polyp	Risk of developing colorectal cancer	Non-colonic polyposis	Neoplastic lesions other than colorectal cancer
Familial adenomatous polyposis	Autosomal dominant	*APC* (5q22.2)	1/20,000–1/10,000	Usually ≥ 100 (< 100 in attenuated type)	Adenoma	Approximately 50% in those in the mid-40 s, nearly 100% in those aged > 60 years	Stomach and duodenum	Gastric cancer, duodenal/ampullary cancer, desmoid tumor, osteoma, papillary thyroid cancer, brain tumor, hepatoblastoma, etc.
*MUTYH*-associated polyposis	Autosomal recessive	*MUTYH* (1p34.1)	Unknown	10–100	Adenoma (concomitant serrated adenoma)	43–100% until age 60 years	Duodenum	Duodenal/ampullary cancer, thyroid cancer, sebaceous tumor, etc.
Polymerase proofreading-related polyposis	Autosomal dominant	*POLE* (12q24.33) *POLD1* (19q13.33)	Unknown	0–70	Adenoma	Average age, 35–40 years	Duodenum	*POLE*: duodenal cancer, brain tumor *POLD1*: endometrial cancer, breast cancer, brain tumor
Lynch syndrome	Autosomal dominant	*MLH1*(3p22.2) *MSH2*(2p21–p16.3) *MSH6*(2p16.3)*PMS2*(7p22.1) *EPCAM*(2p21)	2–4% of all colorectal cancers	Usually less than a few	Adenoma	Mid-40 s (different for causative gene)	None	Endometrial cancer, gastric cancer, ovarian cancer, small-bowel cancer, bile duct cancer, pancreatic cancer, renal pelvis and ureter cancer, brain tumor, sebaceous gland tumor, etc.
Peutz–Jeghers syndrome	Autosomal dominant	*STK11* (19q13.3)	1/280,000–1/8,300	0 to several dozens	Hamartoma	Lifetime risk of 39%	Stomach and small-bowel	Gastric cancer, small-bowel cancer, pancreatic cancer, breast cancer, cervical adenocarcinoma, etc.
Juvenile polyposis syndrome	Autosomal dominant	*SMAD4* (18q21.2) *BMPR1A* (10q23.2)	1/100,000–1/16,000	0 to several dozen	Hamartoma	Lifetime risk of 39–68%	Stomach and small-bowel	Gastric cancer, small-bowel cancer, pancreatic cancer, etc.
Cowden syndrome/PTEN hamartoma tumor syndrome	Autosomal dominant	*PTEN* (10q23.31)	1/200,000	0 to several dozens	Hamartoma; ganglioneuroma; adenoma; hyperplastic polyp	Lifetime risk of 9–16%	Esophagus, stomach, and small-bowel	Breast cancer, endometrial cancer, papillary and follicular thyroid cancer, renal cancer, etc.
Li–Fraumeni syndrome	Autosomal dominant	*TP53* (17p13.1)	Unknown	Less than a few	Adenoma	Unspecified	None	Bone and soft tissue sarcoma, adrenocortical tumor, brain tumor, leukemia, breast cancer, etc.
*MSH3*-associated polyposis	Autosomal recessive	*MSH3* (5q14.1)	Unknown	Dozens	Adenoma	Unspecified	Duodenum	Goiter, intraductal papilloma, gastric cancer, brain tumor, etc.
*NTHL1*-associated polyposis	Autosomal recessive	*NTHL1* (16p13.3)	Unknown	Dozens	Adenoma	Unspecified	None	Breast cancer, endometrial cancer, bladder cancer, head and neck cancer, skin cancer, etc.

Adapted from Reference [15]

##### [Mechanisms of tumorigenesis]

**Fig. 2:**
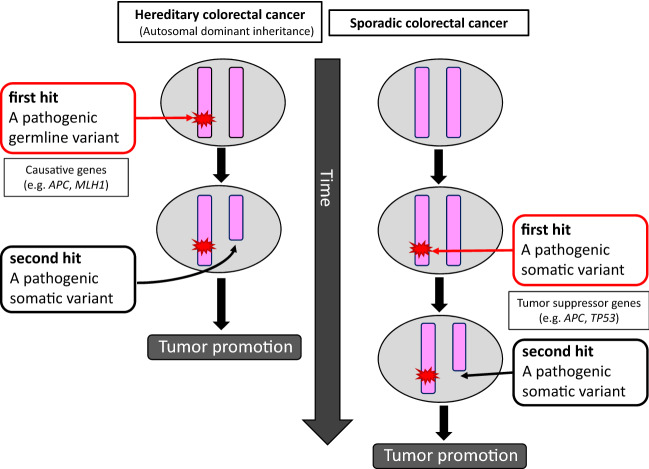
Tumorigenesis mechanism in the two-hit theory of tumor suppressor genes by Knudson

**Fig. 3:**
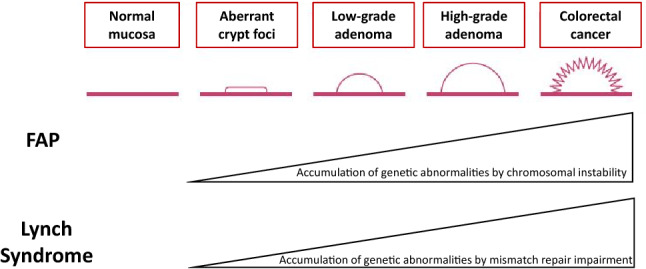
Representative tumorigenesis mechanism of FAP of Lynch syndrome

- Representative hereditary colorectal cancers and their causative genes are shown in Table 3. The causative genes of these diseases are roughly classified into tumor suppressor gene groups (e.g., *APC, TP53*, *PTEN*, *SMAD4*) and repair gene groups associated with base mismatches and base substitutions. Based on the two-hit theory [16] proposed by Knudson, tumorigenesis is thought to occur with the loss of function of the original protein as a result of having pathogenic variants on both alleles of the causative gene. Hereditary colorectal cancers with an autosomal dominant inheritance already have a pathogenic germline variant on one allele (first hit), and when acquired changes occur, such as loss of heterogeneity (LOH) (Side Memo 1: Loss of heterozygosity) or a pathogenic variant on the other allele (second hit), this is thought to facilitate tumorigenesis at a younger age than that for sporadic colorectal cancer (Fig. 2). In the case of FAP, it is thought that aberrant crypt foci (ACF) (Side Memo 1: Aberrant crypt foci) develop when the somatic variant occurs as second-hit in the epithelial cells of the large intestine [17].
- Hereditary colorectal cancers with an autosomal recessive inheritance include MAP, *MSH3*-associated polyposis, and *NTHL1*-associated polyposis. Thus, these diseases develop when each of the alleles with pathogenic variants is inherited from parents who are carriers of pathogenic germline variants in the causative gene.
- The discovery of the *APC* gene, which is the causative gene of FAP, was proposed as the primary mechanism for carcinogenesis of sporadic colorectal cancer, referred to as adenoma–carcinoma sequence [18]. Therefore, hereditary colorectal cancers are also multistage carcinogenic models where multiple genetic abnormalities accumulate due to chromosomal instability (CIN) (Side Memo 1: Chromosomal instability) and MSI, in addition to the causative gene (Fig. 3).
- In addition to the abnormalities in the allele on one side of the *APC* gene in the germline, tumors in FAP have alterations relating to loss of function in the other allele at the somatic level.
- Abnormal APC protein function in patients with FAP increases the translocation of β-catenin accumulated in the cell from the cytoplasm into the nucleus and forms a complex with TCF4, which in turn promotes transcription of oncogenes, leading to cell growth.
- In patients with FAP, the development of colorectal cancer via adenomas from ACF involves alterations in genes related to carcinogenesis, such as the *KRAS* and *TP53* genes [18].
- In patients with Lynch syndrome, a pathogenic germline variant is present in an allele on one side of the mismatch repair gene, and the acquired additions of alterations relating to loss of function in the allele on the other side impair the mismatch mechanism. This results in repeated abnormalities (instabilities) frequently occurring in microsatellite regions, which are simple repeat sequences in the genome. Regions that encode gene products (proteins) involved in tumor suppression (e.g., *TGFBR2*), cellular growth, DNA repair (e.g., *MSH3*, *MSH6*), and apoptosis (e.g., *BAX*) contain repeat sequences, and genetic changes are likely to occur in these regions.
- One of the causative genes of Lynch syndrome, *MLH1* gene, is inactivated by hypermethylation in approximately 6% [19] of all colorectal cancers in the Japanese population, and this is the main cause of sporadic colorectal cancers expressing MSI-H (Chapter III 2-2: Diseases requiring differentiation).

##### [Mechanisms of tumorigenesis]

Side Memo 1

- ■ Variant

A “variant” is a term that refers to the variety of genetic information, mainly in DNA base sequences that differ from the reference sequence. A similar term is “mutation,” but there are inconsistencies in its use, for example, when it is used both in expressions of biological significance and not. Therefore, the term “mutation” should be avoided as much as possible, and “variant” should be used instead. Modifiers, such as “pathogenic,” “benign,” and “uncertain significance” are added to evaluate biological or clinical significance.

- ■ Germline and somatic variants

DNA sequence changes that are inherited through a sperm or ovum are called germline variants. The same change is present in all cells throughout the body because the change is present at the time the ovum is fertilized. In contrast, a change in the sequence of a new nucleotide in a cell other than a germ cell (somatic cell) that constitutes the body is called a somatic variant.

- ■ Genetic testing

Because the term “genetic testing” cannot be distinguished between “genetic testing of somatic cells” and “genetic testing of germline cells,” it was proposed by the Gene-related Test Standardization Experts Committee of the Japanese Committee for Clinical Laboratory Standards that the former be referred to as “somatic cell genetic testing” and the latter as “genetic testing.” These have been classified and defined in the “Guidelines for Genetic Tests and Diagnoses in Medical Practice” (http://jams.med.or.jp/guideline/genetics-diagnosis.html) of the Japanese Association of Medical Sciences, created by associations related to genetic medicine.

- ■ Penetrance

Penetrance is the probability of disease onset in carriers of the genotype of the causative gene in a genetic disorder. Onset at 100% establishment is called complete penetration.

- ■ Chromosomal instability (CIN)

CIN represents abnormalities in the number or structure (deletion, duplication, translocation, etc.) of chromosomes seen in cancer and other cells, and it is thought to cause tumorigenesis.

- ■ Loss of heterozygosity (LOH)

Heterozygosity indicates the presence of different base sequences in a homologous region of a pair of genetic information inherited from the parents. In the case of FAP, pathogenic variants are present only in one of the two alleles of the *APC* gene, and the other allele is normal (wild type) in normal cells. This state is called heterozygosity. However, loss of the wild-type *APC* allele by deletion, referred to as LOH, occurs during the process of oncogenesis.

- ■ Aberrant crypt foci (ACF)

ACF cannot be distinguished from normal mucosa by normal endoscopic observation, and can only be confirmed by magnifying endoscopy as clusters of abnormal crypts showing strong staining with methylene blue. Some ACF are thought to be precursor lesions of adenomas and/or carcinomas.

## Diagnosis

- Patients with suspected hereditary colorectal cancer are screened among patients with colorectal cancer. In the case of polyposis, it is easy to distinguish the disease by obtaining histological diagnosis of adenomatous polyposis or hamartomatous polyposis using biopsy during colonoscopy.

Unlike polyposis syndromes, some hereditary colorectal cancers such as Lynch syndrome and LFS are indistinguishable from sporadic colorectal cancers because of the small number of colorectal polyps. Therefore, it is important to collect information for suspicion of hereditary colorectal cancer, such as medical history and family history and histopathological diagnosis of the resected specimen.

Diagnosis of Lynch syndrome requires genetic testing, which is not currently covered by the national health insurance program in Japan. If hereditary colorectal cancer is confirmed or suspected, genetic counseling should be provided to the patient and family members (relatives), especially first-degree relatives (parents, offspring, and siblings).

### Diagnostic flow

#### STEP 1

##### Risk assessment of hereditary colorectal cancer: inquiry and endoscopy

**Fig. 4:**
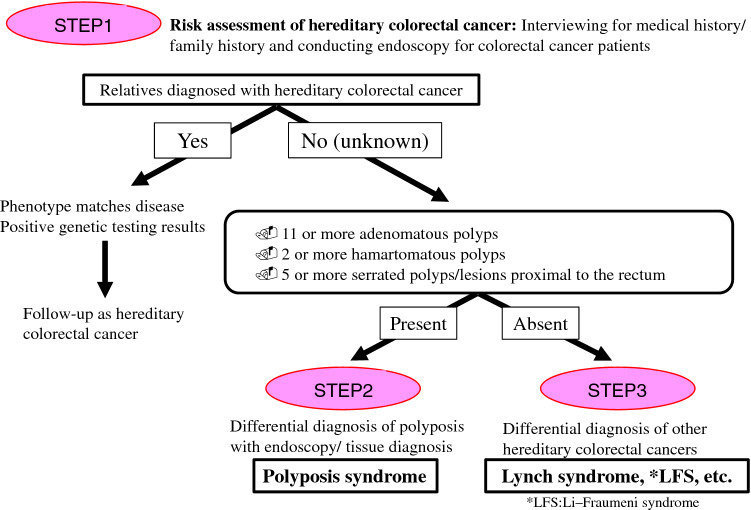
Risk assessment of hereditary colorectal cancer

- In order to select candicates of hereditary colorectal cancer in clinical practice, it is essential to listen to the individual’s disease history, such as early onset, synchronous/metachronous colorectal cancer, and history of malignancy other than colorectal cancer, and conduct interview on the family history of a third-degree relative (at least second-degree relative) on both the maternal and paternal sides of the family. With the progression of the nuclear family, it may be difficult to obtain information of even second-degree relatives; therefore, we recommend collecting the information needed to distinguish the paternal and maternal sides to assess the mode of inheritance and create a pedigree (Appendix I: Principles for writing and reading pedigrees).
- If a known causative gene variant has been identified in the family of a patient with suspected hereditary colorectal cancer, the patient should be followed for hereditary colorectal cancer if the patient has a phenotype consistent with the disease or if genetic testing confirms the disease- causing pathogenic variant (Fig. 4). If these are not true, the risk assessment [13] for hereditary colorectal cancers (Fig. 4) may proceed to STEP 2 if one of the following high-risk factors is present during colonoscopy:
  - Eleven or more adenomatous polyps.
  - Two or more hamartomatous polyps.
  - Five or more serrated polyps/lesions proximal to the rectum.
- For hereditary colorectal cancers other than polyposis, it is essential to have a medical history and family history, for example, for patients with suspected Lynch syndrome. In this case, proceed to STEP 3.

#### STEP 2

##### [Differential diagnosis of colorectal polyposis (Fig. 5)]

- Differentiation between disease groups of adenomatous polyposis, hamartomatous polyposis, and serrated polyposis are made with histological diagnosis of colonic polyps using biopsy during colonoscopy.
- FAP is first considered among adenomatous polyposis when the number of polyps is ≥ 100. When the number of adenomatous polyps is between 11 and 100, candidates, in order of incidence, include (1) attenuated FAP (AFAP), (2) MAP, and (3) PPAP. Genetic testing should be performed after a family history is obtained for definitive diagnosis because these types of polyposis cannot be differentiated by endoscopic findings.
- A family history of an autosomal dominant mode of inheritance is a useful information in the diagnosis of adenomatous polyposis. The presence or absence of concomitant symptoms, such as fundic gland polyposis, duodenal adenoma, exostosis, and congenital retinal epithelial hypertrophy, is also helpful. Meanwhile, the possibility of autosomal recessive inheritance diseases (e.g., MAP), somatic *APC* mosaicism, probands with a *de novo* variant, etc., is examined when the disease is not observed in the parents.
- The number of gastrointestinal polyps in the hamartomatous polyposis group ranges from several to several dozens for PJS and JPS and > 50 for a majority of CS/PHTS. Polyps of PJS and JPS exhibit histologically specific morphology and are included in the clinical diagnostic criteria along with the number of polyps [13]. Each disease (except for digestive tract polyposis) has various concurrent disease states, so diagnosis according to each of the diagnostic criteria may be possible.

**Fig. 5:**
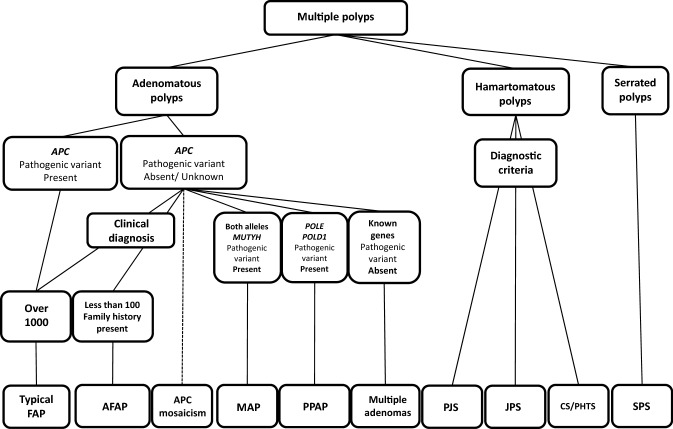
Flowchart of the diagnosis of hereditary colorectal polyposis. FAP familial adenomatous polyposis, AFAP attenuated FAP, MAP MUTYH-associated polyposis, PPAP polymerase proofreading-associated polyposis, PJS Peutz–Jeghers syndrome, JPS juvenile polyposis syndrome, CS/PHTS Cowden syndrome/PTEN hamartoma tumor syndrome, SPS serrated polyposis syndrome

#### STEP 3

##### [Differential diagnosis of other hereditary colorectal cancers]

- An important method in the diagnosis of Lynch syndrome and LFS is to use interviews to check for relatives who are affected by colorectal cancer and other Lynch syndrome-associated tumors, breast cancer, bone and soft tissue tumors, and brain tumors that are highly prevalent in LFS, respectively, across generations.

### Genetic testing

#### [Clinical significance]

- Among adenomatous polyposis cases, typical FAP can be diagnosed from clinical symptoms, whereas other disorders can be definitively diagnosed by identifying pathogenic variants of the causative gene through genetic testing.
- Genetic testing to identify pathogenic variants of the causative gene is essential for diagnosis of Lynch syndrome.
- If the pathogenic variant of the causative gene can be identified in the proband with hereditary colorectal cancer, this enables the diagnosis of their relatives.

##### [Testing methods]

- Blood should be drawn in the amount required for gene analysis (usually 2–10 mL).
- Genetic testing involves the analysis of the sequences of the exons and exon–intron boundary regions that encode the proteins of the causative genes of the disease. Relatively large exon-level deletions and duplications are analyzed using a multiplex ligation-dependent probe amplification (MLPA) method.
- If genetic abnormalities of the proband have been identified, analysis of only the variant sites (single site) in their relatives may be conducted for genetic testing.

##### [Types of tests]

- Genetic testing of hereditary colorectal cancer includes (1) testing for only one presumed causative gene for a suspected hereditary tumor; (2) testing for more than one presumed causative gene for a suspected hereditary tumor; (3) multi-gene panel testing for a set of causative genes, including those required for differential diagnosis; (4) multi-gene panel testing that comprehensively encompasses the causative gene for a hereditary tumor; and (5) whole exome sequencing or whole genome sequencing (Table 4).
  - As an example of (1), genetic testing only for the causative gene, *APC*, is performed if FAP is suspected.
  - As an example of (2), testing for a set of genes including the analysis of *EPCAM* gene deletion, which is adjacent to the upstream region of the *MSH2* gene, induces aberrant methylation in the *MSH2* gene promoter region, and contributes to the loss of MSH2 protein expression, is performed in addition to that for *MLH1*, *MSH2*, *MSH6*, and *PMS2* when Lynch syndrome is suspected.
  - As an example of (3), dozens of multiple colorectal polyps have multiple possible hereditary tumors that are difficult to distinguish with clinical findings. In such cases, it is possible to simultaneously and efficiently identify the cause by examining the gene set containing the causative gene of the disease. If the cause is still unidentifiable, then (4) multi-gene panel testing that comprehensively contains the causative gene for the hereditary tumor or (5) whole exome sequencing, whole genome sequencing may be performed.

**Table 4:** Types of genetic tests

1) Testing for only one presumed causative gene for a suspected hereditary tumor
e.g., *APC* for FAP
2) Testing for more than one presumed causative gene for a suspected hereditary tumor
e.g., *MLH1*/*MSH2*/*MSH6*/*PMS2*/*EPCAM* for Lynch syndrome
3) Multi-gene panel testing for a set of causative genes of a disease requiring differential diagnosis
4) Multi-gene panel testing that comprehensively encompasses the causative gene for a hereditary tumor
5) Whole exome sequencing or whole genome sequencing

- Genetic testing for hereditary colorectal cancer is currently not covered by the national health insurance program in Japan but can be contracted through self-pay treatment or implemented at the research level in some facilities.
- Oncogene panel testing for patients with solid cancers without standard treatment (e.g., cancer of unknown primary or rare cancer) or solid cancers for which standard treatment was completed (including patients with end-stage cancer) was covered by the national health insurance program in May 2019 in Japan. These oncogene panel tests (Table 5) are originally aimed at searching for effective drugs, but hereditary colorectal cancer can also be diagnosed since they also include its causative genes.

**Table 5:** Genes responsible for hereditary colorectal cancer included in oncogene panel testing

・ OncoGuide NCC-Onco Panel System™
*APC, MLH1, MSH2, POLD1, POLE, PTEN, SMAD4, STK11, TP53*
・ FoundationOne CDx Cancer Genome Profile
*APC, MLH1, MSH2, MSH3, MSH6, MUTYH, PMS2, POLD1, POLE, PTEN, SMAD4, STK11, TP53*

##### [Explanation of the results]

- The clinical relevance of the detected variants is generally assessed using classifications from ClinVar (https://www.ncbi.nlm.nih.gov/clinvar/) or InSiGHT (https://www.insightgroup.org/variants/databases/) (Table 6).

A) If “pathogenic variant or likely pathogenic variant”

- Equivalent to pathogenic or likely pathogenic.

- Medical management for a genetic disorder is performed. However, it should be understood that individuals who have a pathogenic variant will not always develop cancer during their lifetime, except for diseases that have a nearly 100% penetrance, such as FAP.

B) If “variants of uncertain significance (VUS) were detected”

- Equivalent to uncertain significance.

- Genetic changes whose impact on diseases is unknown are reported as VUS. Examples include silent variants (where a single change in the base sequence does not affect amino acid synthesis) or missense variants (where amino acid substitution occurs), which do not affect disease development. It is recommended in such case to proceed with the following section, “Genetic abnormalities are not detected,” until the significance of this variant is proven.

C) When “genetic abnormalities are not detected”

- Includes benign or likely benign.

**Table 6:** Classification of gene variants

ClinVar	InSiGHT
Clinical significance	MMR gene variant
Value	Classification criteria
Pathogenic	Class 5, pathogenic
Likely pathogenic	Class 4, likely pathogenic
Uncertain significance	Class 3, uncertain
Likely benign	Class 2, likely not pathogenic/little clinical significance
Benign	Class 1, not pathogenic/no clinical significance

##### [When genetic abnormalities are confirmed in a family]

- If the same genetic changes were not found in a pedigree where a definitive diagnosis was conducted with genetic testing, it is determined that this is not a genetic disease found in the family. It should still be understood in such cases that there is a tumorigenesis risk in the general population.

##### [When no diagnosis is made in the family]

- Given that they may have variants that cannot be detected by the methods used for genetic testing or may be due to abnormalities in unknown causative genes, these should be treated with caution. For example, the clinical detection rate of pathogenic variants of the *APC* gene even in FAP with 100–1000 polyps does not reach 100%, with values approximately 60% [20]. Therefore, the possibility that unknown variants have not been detected due to technical problems or presence of other gene abnormalities must be considered. In clinical practice, this should ideally be managed similar to genetic diseases when clinically considered to be a genetic disease even in cases where pathogenic variants were not detected with genetic testing.

### Genetic counseling

#### [Overview]

- Genetic counseling requires adhering to the three principles of counseling: receptive attitude, nondirective responses, and empathic understanding. Genetic counseling relieves anxiety in family members who have genetic problems by providing accurate medical knowledge in an easy-to-understand manner. Note that consultants’ thoughts, sensitivities, prior knowledge, comprehension, magnitude of anxiety, and sense of trust in healthcare vary between individuals.
- Unlike sporadic colorectal cancer, hereditary colorectal cancer requires long-term surveillance under specialized medical management because it has a variety of concomitant lesions. Therefore, referral to a specialized center with an established genetic counseling and testing system should be considered for patients with characteristics of hereditary colorectal cancer.
- It is imperative to explain the clinical significance of genetic testing and have patients understand the advantages and problems of testing (Table 7) prior to its implementation.
- Since genetic testing for the causative gene of hereditary colorectal cancer is not covered by the national health insurance program in Japan and is a self-pay treatment, it should be explained that the cost is high and there are testing limitations in that the pathogenic variant is not always detectable.
- Genetic testing is performed after a written description of the test and consent form are created, and informed consent is obtained from the patient, while taking care not to burden the client from various medical, ethical, economic, and technical perspectives. Genetic counseling should be continued as necessary and before and after genetic testing.
- The client’s desire to have a family member present should be confirmed at the time of disclosure of genetic testing results. Confirm a time and place with the individual if family presence is not desired.
- Genetic counseling should be provided to family members (relatives) in addition to the patient.
- First-degree relatives (parents, offspring, and siblings) should be fully informed about the disease, and informed consent should be obtained for surveillance of associated tumors according to risk assessment.
- Genetic testing should be conducted in accordance with the “Guidelines for Genetic Tests and Diagnoses in Medical Practice” (http://jams.med.or.jp/guideline/genetics-diagnosis.html) by the Japanese Association of Medical Sciences, “Genetic Testing Research Relating to Familial Tumors and Guidelines Relating to its Application in Treatment (2019 Edition)” (http://jsht.umin.jp/information/opinion/download/guideline2019040101.pdf) by the Japanese Society for Hereditary Tumors, and “Ethical Guidelines Relating to Human Genome/Gene Analysis Research” (https://www.mhlw.go.jp/file/06-Seisakujouhou-10600000-Daijinkanboukouseikagakuka/0000153405.pdf) (implemented on April 1, 2001; partially revised on February 28, 2017) compiled by the Japanese Ministries of Education, Culture, Sports, Science and Technology; Health, Labour and Welfare; and Economy, Trade and Industry. In addition, the privacy of the test subject shall be considered, and the records shall be stored carefully.

**Table 7:** Advantages and problems of genetic testing

Benefits	Problems
A definitive diagnosis of the disease is obtained	Genetic testing has limitations and may not be able to detect the disease
The examination is performed using a small amount of blood	Even if a diagnosis is obtained, the disease onset cannot be always predicted
It can be diagnosed regardless of the presence or absence of family history	It is considered self-pay treatment
Surveillance is available for diseases that can be affected	
Blood tests can be used to diagnose relatives and check whether they are affected	

##### [Content of counseling]

- Clients need to provide a variety of information on cancer and inheritance in order for them to have an accurate understanding of hereditary colorectal cancer (Table 8). Attention should also be paid to the psychological effects and considerations for social discrimination on the person and family that may be brought about by the results of genetic testing.
- Consider the appropriate timing of genetic testing, based on the time of onset of the tumor that develops in relation to inheritance. For example, the time of genetic testing for Lynch syndrome—is generally beyond adulthood due to its onset generally being after adulthood. Surveillance for Lynch syndrome-associated tumors should be conducted for relatives who have been definitively diagnosed through genetic testing and suspected relatives who did not undergo genetic testing.
- The risk of developing hereditary colorectal cancer varies greatly depending on the disease and type of variant of causative gene.
- Patients with hereditary colorectal cancer requires long-term surveillance.

**Table 8:** Provision of counseling information

Content of information provided during counseling
1) Relevant genetic disorders
Reasons for suspected genetic disorders – onset factors (environment, inheritance)
Genes responsible for diseases
Inheritance – autosomal dominant (or recessive) inheritance, the probability that a relative has a pathogenic variant
Characteristics of hereditary tumors – cancer penetrance
Countermeasures against cancer – prevention, early detection, and treatment
2) Genetic testing
Testing purpose, methods, test accuracy and detection rate, test limitations and uncertainties, and costs
Expected benefits – elimination of anxiety due to uncertainty from confirmation, prediction of risk of onset, prophylactic treatment, and helpfulness in diagnosing onset in relatives
Psychological effects on the person and family and likelihood for inheritance in children
Future measures and options if not tested
Response to family members (relatives)
Genetic counseling should be offered to family members (relatives) and the patient
First-degree relatives (parents, offspring, and siblings) should be fully informed about the disease, and informed consent should be obtained for surveillance of the gastrointestinal tract, especially the large intestine
Information on the need for surveillance and significance of genetic diagnosis should be provided

## Chapter II. Familial adenomatous polyposis (FAP)

## Overview

- Familial adenomatous polyposis (FAP) is a hereditary autosomal dominant disease caused by pathogenic germline variants in the *APC* gene and predominantly characterized by the development of multiple colorectal adenomas.
- If not treated, almost all patients with FAP develop colorectal cancer.
- Various associated tumorous and non-tumorous lesions in the gastrointestinal tract and other organs can develop in addition to colorectal cancer.

### Comments

#### [Clinical features]

- Some patients with FAP develop colorectal cancer while still in their teenage years, while approximately 50% of the patients develop colorectal cancer in their 40 s. If not treated, almost all patients with FAP develop colorectal cancer at approximately 60 years of age [21].
- The leading cause of death [22] in patients with FAP is colorectal cancer, which accounted for approximately 80% of all causes of death in patients with FAP until the 1980s; however, the proportion has been decreasing toward approximately 60% since the 1990s.
- Among the main extracolonic manifestations (Table 9), desmoid tumors and duodenal cancer are the leading causes of death of patients with FAP other than colorectal cancer, with an incidence of approximately 10 and 6%, respectively [22].

**Table 9:** Major neoplastic lesions associated with FAP

Fundic gland polyposis^a^	Skull osteoma/jaw osteoma/unerupted teeth/extra teeth (supernumerary teeth)
Gastric adenoma^a^	Epidermoid cyst
Duodenal adenoma^a^	Thyroid cancer
Ampullary adenoma^a^	Congenital hypertrophy of retinal pigment epithelium
Jejunal/ileal adenoma^a^	Hepatoblastoma
Desmoid tumor	Adrenal tumor
	Brain tumor

^a^Possibility of malignant transformation

##### [Causative gene]

- *APC* gene on chromosome 5 (5q22. 2).

##### [Mode of inheritance]

- Autosomal dominant inheritance.

## [Incidence]

- The estimated incidence of FAP in the general population is 1:20,000 to 1:10,000 in Western countries and 1:17,400 in Japan [23]. Less than 1% of all patients with colorectal cancer are estimated to have FAP [12].

## Diagnosis

## Flow of diagnosis (Chapter I, Fig. 5: Flowchart of hereditary colorectal polyposis diagnosis)

### FAP can be diagnosed clinically and/or genetically

#### [Clinical diagnosis]

If either of the following criteria (1) or (2) is satisfied, a diagnosis of FAP is made:

1. Detection of approximately ≥ 100 adenomas in the large intestine, irrespective of the presence or absence of a family history of FAP.
2. Detection of < 100 adenomas in the presence of a family history of FAP.

##### [Genetic diagnosis] (Side Memo 1)

If a pathogenic germline variant is present in the *APC* gene, a diagnosis of FAP is made.

#### Comments

- There are exceptional pathologies other than FAP that are characterized by the presence of approximately ≥ 100 adenomas in the large intestine (*MUTYH*-associated polyposis (MAP), an autosomal recessive disease). Therefore, a family history consistent with autosomal dominant inheritance is a useful clue for the diagnosis of FAP.
- The presence of characteristic extracolonic manifestations is a useful clue for the diagnosis of FAP, irrespective of the number of colorectal adenomas.
- No variants are detected in the *APC* gene in 20–40% of patients clinically diagnosed with FAP [20, 24].
- If a patient prefers to undergo genetic testing for their own treatment or diagnosis in their relatives or if attenuated FAP (AFAP) has to be differentiated from *MUTYH*-associated polyposis (MAP) and polymerase proofreading-associated polyposis (PPAP), genetic testing of the *APC* gene is considered. This testing can be performed in testing companies (not covered by the Japanese national health insurance program) (Side Memo 2: *APC*-associated polyposis) (CQ1).

## Comments

Side Memo 2 ***■ APC*-associated polyposis**

Polyposis caused by pathogenic germline variants in the *APC* gene is sometimes referred to as *APC*-associated polyposis [25]. *APC*-associated polyposis also includes gastric adenocarcinoma and proximal polyposis of the stomach (GAPPS) caused by pathogenic variants in the promoter 1B of the *APC*.

### ■ GAPPS

GAPPS is caused by pathogenic germline variants in the 1B promoter region of the *APC* gene, with fundic gland polyposis as the presenting feature [26]. Since the 1A promoter that controls APC expression in the colon and rectum is inactivated by methylation in the stomach tissue, an aberration of the 1B promoter suppresses APC expression solely in the stomach [25].

## Classification by adenoma burden

FAP is sometimes classified as severe FAP, sparse FAP, and AFAP based on the density of the adenomas. Severe and sparse FAPs are sometimes collectively called classical (typical) FAP.

The adenoma burden has been associated with the site of the germline variant in the *APC* gene and risk of development of colorectal cancer.

## Comments

**Fig. 6:**
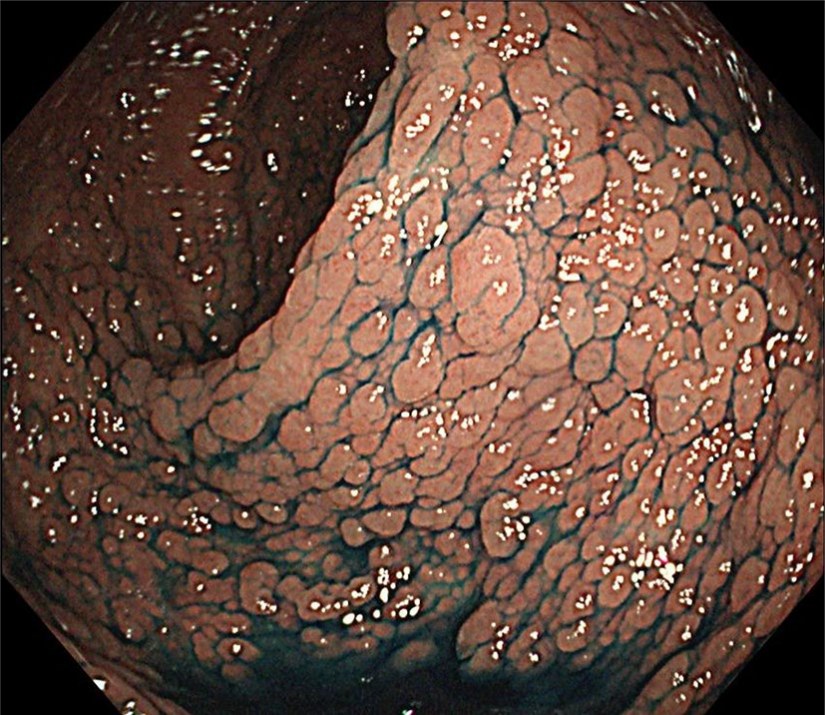
Severe/profuse/dense FAP

**Fig. 7:**
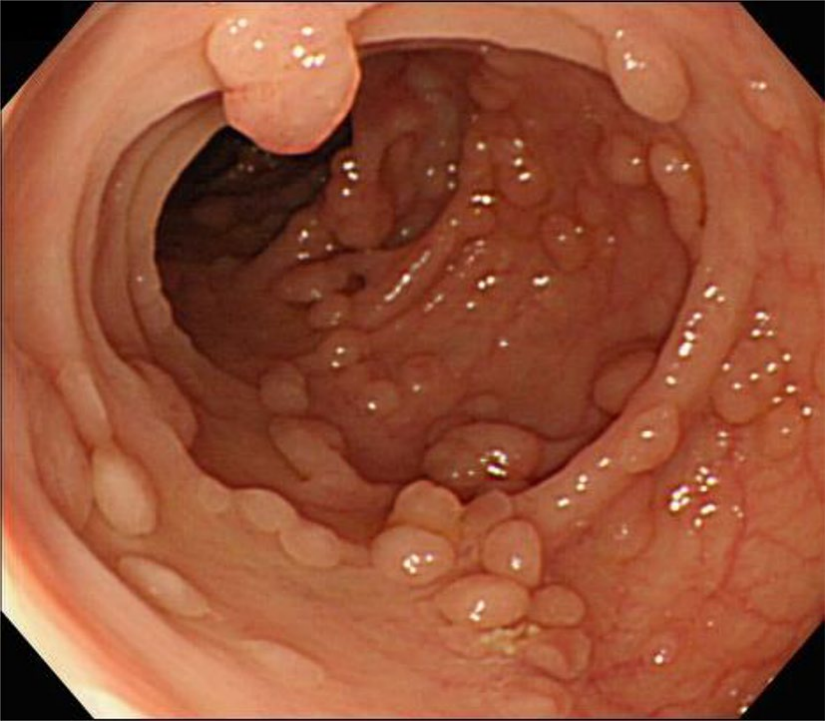
Sparse FAP

- Severe/profuse/dense FAP: a phenotype in which normal colorectal mucosa cannot be macroscopically or endoscopically observed because of numerous adenomas (Fig. 6) (Side Memo 3: Difference between severe and sparse types). However, adenoma density often differs even among regions of the large intestine.
- Sparse FAP: a phenotype in which multiple (> 100) adenomas can be counted but normal colorectal mucosa is visualized (Fig. 7).
- AFAP: a phenotype in which the number of adenomas ranges from 10 to 100.
- In cases of severe FAP, a germline variant is often detected between codons 1250 and 1464 (in particular, codon 1309) [27, 28] in the *APC* gene. In AFAP, the germline variant is often detected in the alternative splicing region (in which an exon is skipped during transcription because of the variant) or 5′ or 3′ region of the *APC* gene [29].
- According to the JSCCR multicenter study, the age at diagnosis of adenomas and age at diagnosis of cancer in the colorectum are often lower in patients with severe FAP than in those with other types of FAP. Approximately half of the patients with severe, sparse, and attenuated types develop colorectal cancer by age 40, 47, and 55 years, respectively.

Side Memo 3 **■ Difference between severe and sparse types**

FAP is sometimes classified based on the number of colorectal adenomas: severe (> 1,000 or 2,000 adenomas) and sparse (100–1,000 or 2,000 adenomas) types. Many studies classified these types of FAP as typical FAP, and FAP associated with a smaller number (10–99) of adenomas are categorized as attenuated FAP (AFAP). Strict differentiation between severe and sparse types is of little clinical significance.

### Diseases and conditions that should be differentiated from FAP

#### Somatic *APC* mosaicism

The presence of somatic variants in the *APC* gene during the process of tumorigenesis results in a mosaic of cells with and without the variants in the *APC* gene. If this abnormality occurs in cells that differentiate into mucosal cells of the large intestine, multiple colorectal adenomas would develop, like in FAP. *APC* mosaicism occurs in 1.6–4% of patients with FAP with identified variants in the *APC* gene and 11–20% of patients with FAP without a family history [30, 31]. Detecting low-allele frequencies of pathogenic germline variants has recently become possible using next-generation sequencers, and the low-allele frequency of pathogenic variants has been detected in 25–50% of FAP patients in whom conventional methods failed to detect pathogenic variants [32, 33]. Clinically, this condition is managed as FAP. Moreover, variants in the *APC* gene, if present in some germ cells (sex mosaicism), may be passed on to the next generation.

#### *MUTYH*-associated polyposis (MAP)

MAP is a hereditary autosomal recessive disease [34] caused by biallelic germline variants of the *MUTYH* gene, which is one of the base excision repair genes. MAP is characterized by the presence of approximately 10–100 adenomas in the large intestine, although some patients with MAP could have as many as 100–1,000 adenomas [35]. The incidence of germline variants in the *MUTYH* gene is unknown among Japanese patients with colorectal cancer. The penetrance of colorectal cancer (proportion of patients who develop colorectal cancer among those with gene variants) is 43–100% in patients aged up to 60 years [36]. Some patients with MAP have developed a variety of lesions, such as those found in patients with FAP. In Japan, there are few case reports on MAP, and this disease remains poorly understood. Treatment for MAP is similar to that for patients with AFAP.

#### Polymerase proofreading-associated polyposis (PPAP)

PPAP is a hereditary autosomal dominant disease caused by pathogenic germline variants in the *POLE* or *POLD1* gene, both of which repair errors in DNA replication (proofreading function) [37]. Many patients have a few dozen colorectal adenomas, while some patients have no adenomas. As extracolonic manifestations, duodenal adenomas/cancers and brain tumors have been reported to develop in patients with PPAP carrying variant of the *POLE* gene [38] and endometrial cancers, breast cancers, and brain tumors have been reported to develop in patients with PPAP carrying variants of the *POLD1* gene [39]. Colorectal adenomas and cancers in PPAP are histologically indistinguishable from these tumors in sporadic cases. Therefore, genetic testing is necessary for a definitive diagnosis.

## FAP-associated lesions

### Extracolonic FAP-associated lesions

Neoplastic or non-neoplastic extracolonic lesions are associated with FAP (Table 9).

## Fundic gland polyposis or gastric adenoma

**Fig. 8:**
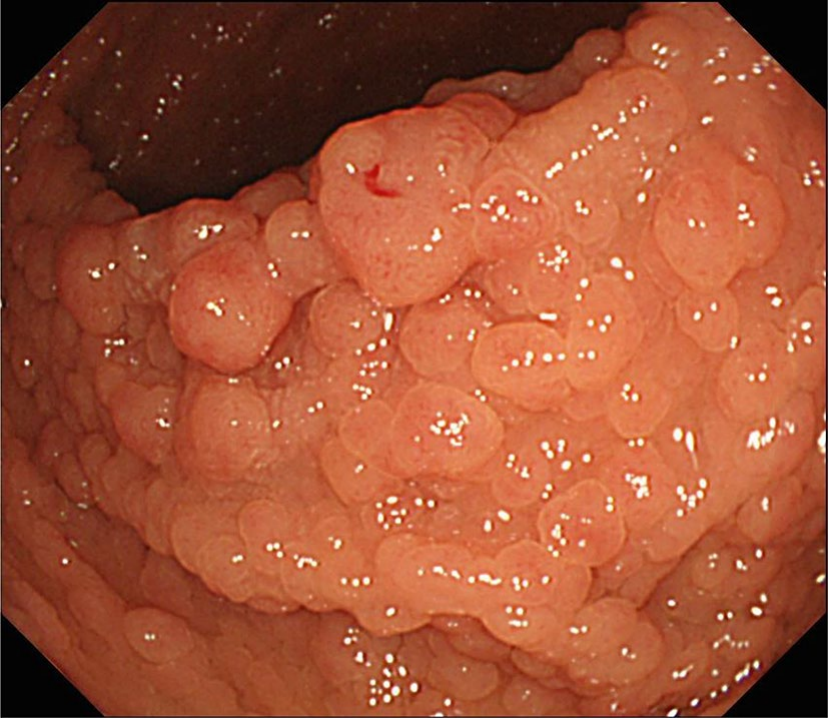
Fundic gland polyposis

**Fig. 9:**
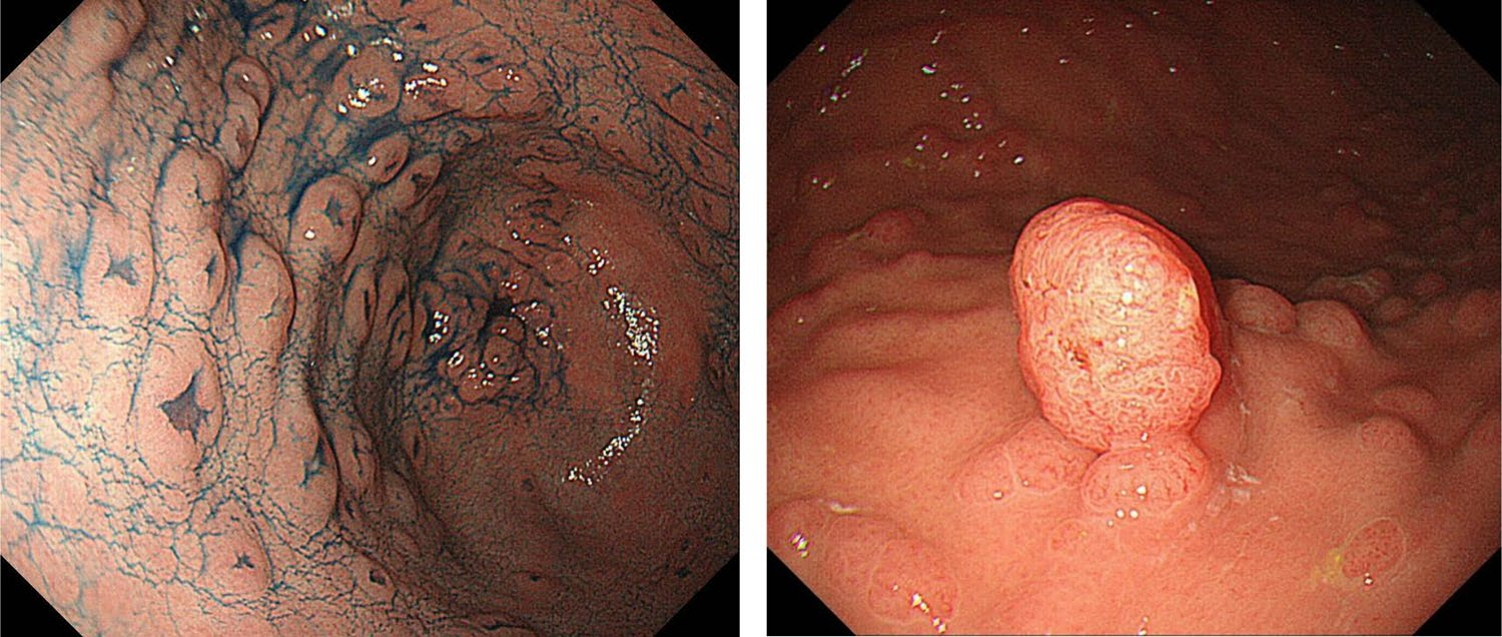
Gastric adenoma (left, depressive type; right, elevated type)

- The presence of fundic gland polyposis (Fig. 8) and gastric adenomas (Fig. 9) (CQ2) is a useful clue for the clinical diagnosis for FAP.
- Patients with FAP without *Helicobacter pylori* (*H. Pylori*) infection often tend to have fundic gland polyposis [40]. Surveillance for fundic gland polyposis is required in patients with FAP because of the risk of malignant transformation of fundic gland polyps in these patients.
- Patients with FAP often develop depressed- or protruded-type gastric adenomas (Fig. 9).

## Duodenal adenoma/cancer

**Fig. 10:**
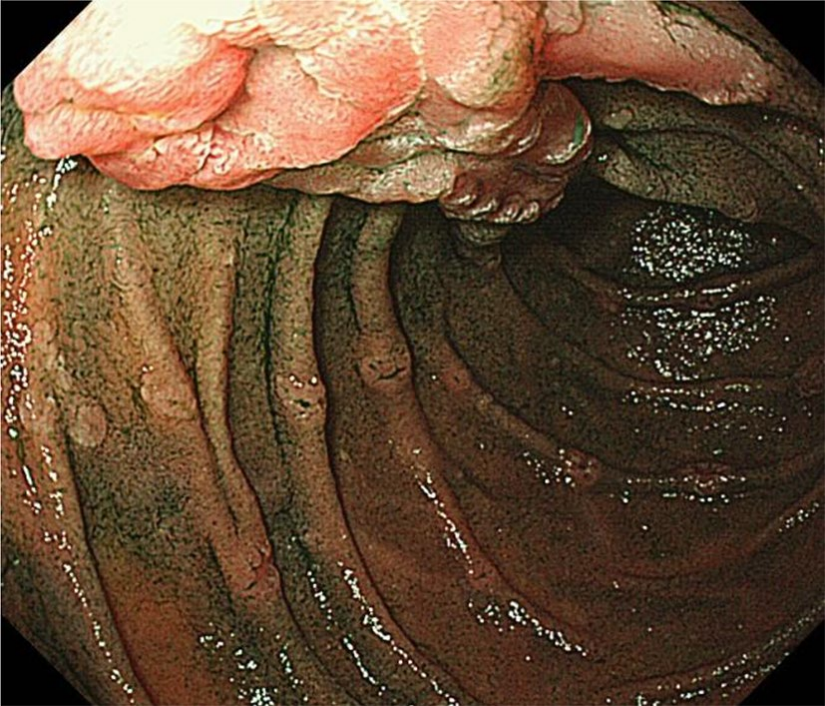
Ampullary adenoma and duodenal adenomas

- The presence of duodenal adenoma (Fig. 10) (CQ2, CQ3) and ampullary adenoma (CQ2) is a useful clue for the clinical diagnosis of FAP.

### [Characteristics]

- Duodenal adenomas are found in 30–90% of patients with FAP [41–43], and adenoma incidence tends to be higher after the age of 40 years [42, 43].
- Duodenal adenomas grow very slowly [42, 44] but require periodic endoscopic surveillance and/or treatment (CQ2).
- Duodenal carcinoma accounts for approximately 3% of the causes of death among patients with FAP [22, 45].
- The relative risk of duodenal cancer in patients with FAP compared with that in the general population is 250–330.8 [46, 47], and the cumulative incidence of duodenal cancer at 57 years of age is approximately 4.5% [48].
- The JSCCR multicenter study found that the cumulative incidence of duodenal adenomas at the age of 50 years was 39.2% and significantly higher in patients with classical FAP than in patients with AFAP (42.5 vs. 23.5%) [49].

#### [Evaluation of duodenal adenoma]

**Fig. 11:**
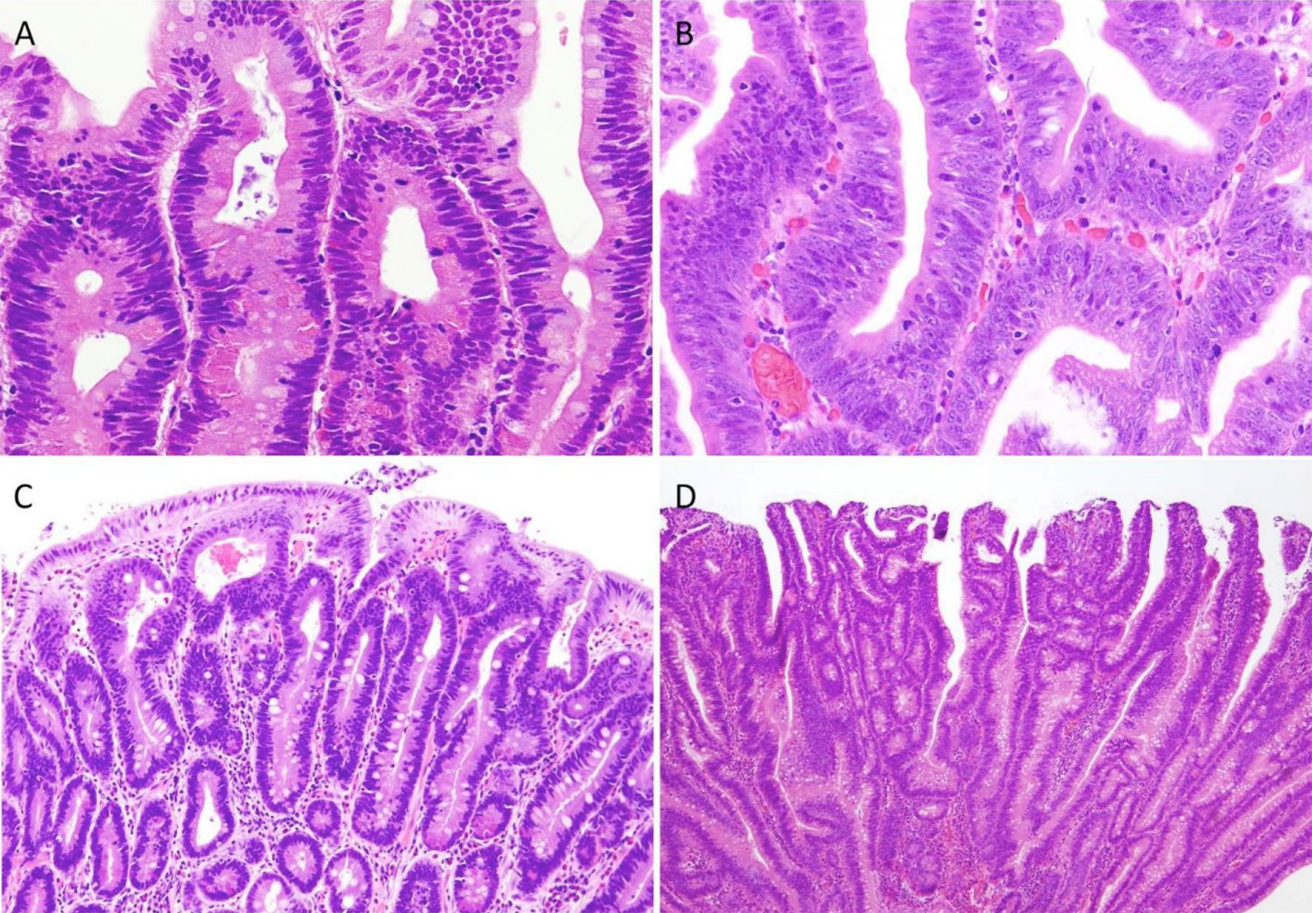
Histology of FAP-associated duodenal adenomas. **a** Low-grade adenoma: the tumor glands are rather uniform and the adenomatous epithelial cells show basally oriented, elongated nuclei. **b** Intramucosal carcinoma: tumor glands show significant irregularity, nuclear stratification, and occasional prominent nucleoli. Note that high-grade dysplasia in the Spigelman classification includes non-invasive intramucosal carcinoma in the Japanese classification. **c** Tubular adenoma: this lesion shows a relatively regular tubular architecture. **d** Tubulo-villous adenoma: this lesion partially exhibits villous architecture, composed of fibrovascular cores lined by dysplastic epithelium

**Fig. 12:**
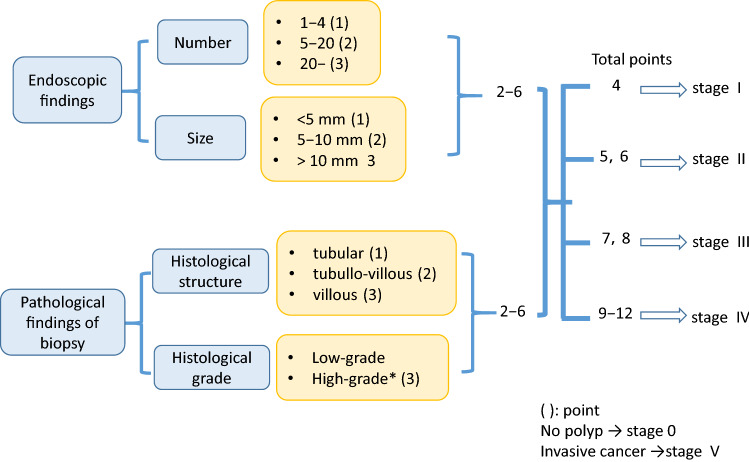
Evaluation of duodenal adenoma by revised Spigelman’s classification

- There exists a clinicopathological classification of duodenal adenomas, called the Spigelman classification [50].
- In the Spigelman classification, the number and maximum diameter of duodenal adenomas are assessed by upper gastrointestinal, endoscopy and biopsy (Fig. 11) is needed to evaluate the histology and severity of dysplasia. Further, some modifications have been introduced to this classification (modified Spigelman classification) [50] (Fig. 12) (Side Memo 4: Changes in the evaluation methods for Spigelman classification).
- Forward- and side-viewing (oblique-viewing) endoscopies are used in the detection of duodenal adenomas.
- The use of narrow band imaging increased the number of duodenal adenomas identified but had no impact on Spigelman classification [51].

##### [Evaluation of duodenal adenoma]

##### [Surveillance]

**Fig. 13:**
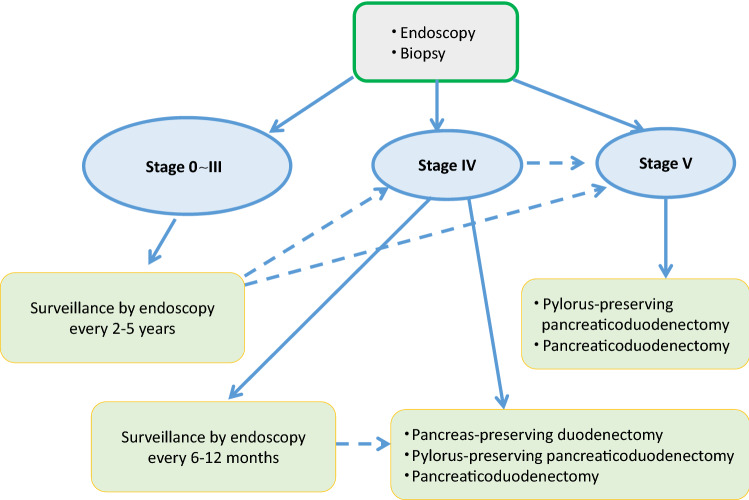
Surveillance of duodenal adenoma based on revised Spigelman’s classification

- No consensus has been reached on the interval of endoscopic surveillance, but it is recommended that endoscopic surveillance should be performed every 4–5 years for cases with stage 0, every 2–5 years for cases with stage I, every 2–3 years for cases with stage II, and every 6 months to 2 years for cases with stage III duodenal adenomas [43, 48].
- A guide for the treatment of duodenal adenomas based on the modified Spigelman classification is shown in Fig. 13.

##### [Treatment]

- There are no clinical trials comparing endoscopic treatment with observation for duodenal lesions in patients with FAP.
- Endoscopic treatments for duodenal lesions (adenomas/noninvasive intramucosal cancer) include snare resection (polypectomy, endoscopic mucosal resection), electrocautery, and argon plasma coagulation.
- Endoscopic electrocautery should be used for adenomas classified as Spigelman stage I/II, but it is alternative treatment if endoscopic or duodenotomy resection is difficult for patients with numerous adenomas [48].
- Complete endoscopic resection of many adenomas classified as Spigelman stage II/III was associated with a high complication rate and recurrence rate of 50–100% [41].
- Assessment of the indication for surgery or surveillance every 6–12 months by a specialist is recommended for patients since malignant transformation occurs in 7–36% of cases [48, 52] with stage IV classification (CQ3).

Side Memo 4 **■ Changes in the evaluation methods for the Spigelman classification**

The Spigelman classification is a staging system for duodenal adenomas associated with FAP that was proposed in 1989 [50]. The adenoma number, size (maximal diameter), histology, and severity of dysplasia are assessed on a scale ranging from 1 to 3, and the total score is used to determine the disease stage. In the Vienna classification of 2000 [53], the grading of the severity of dysplasia was changed from three levels, that is, mild, moderate, and severe, to two levels, namely, low and high grades, and a modified classification was proposed, in which 1 and 3 points are given to the low and high grades, respectively [54]. Recently, the National Comprehensive Cancer Network Guidelines (Genetic/Familial High-Risk Assessment: Colorectal: Version 1. 2020-July, 2020) proposed a classification that was a simpler form of the Spigelman classification or the modified Spigelman classification. This classification consists of stages 0 (no adenomas), I (1–4 tubular adenomas measuring 1–4 mm in diameter), II (5–19 tubular adenomas measuring 5–9 mm in diameter), III (≥ 20 adenomatous lesions measuring ≥ 1 cm in diameter), and IV (dense polyposis or high-grade adenomas). No prospective studies on the validity of endoscopic surveillance or treatment based on these staging systems have been conducted, and this issue needs to be addressed in the future.

## Desmoid tumors

**Fig. 14:**
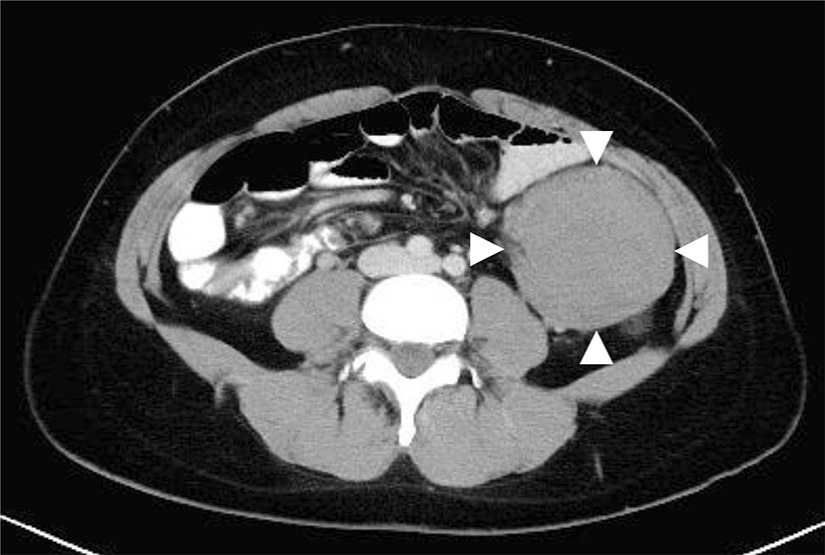
Intra-abdominal desmoid tumor

- Desmoid tumors (Fig. 14) is a useful clue for the clinical diagnosis for FAP.

## [Characteristics]

- Desmoid tumors are a type of fibroma that do not metastasize but tend to show invasive growth.
- Intra-abdominal desmoid tumors account for 70% of all desmoid tumors [55] and often develop in the abdominal wall, mesentery, or retroperitoneum after proctocolectomy (particularly within 2–3 years, median value in the Japanese literature is 26.3 months) [56–58].
- Desmoid tumors are observed in 8–20% of patients with FAP [59–62].
- Desmoids may resolve spontaneously or stabilize [63–65].
- Data from Japan show that the incidence is 10–15% [58, 66]. Intra-abdominal desmoid tumors comprise 71.8–80% of all desmoid tumors, and a significantly higher incidence was observed among those aged < 30 years and in women, with onset within 1 year for approximately two-thirds of this group [58, 66].
- When developing intra-abdominally (including in the retroperitoneum), they can cause bowel obstruction, perforation, abscess formation, ureteral obstruction, etc., often making treatment difficult.
- Past reports have indicated that there are no differences in the risk of developing desmoid tumors depending on surgery. Two recent studies including data from Japan indicated that the risk of developing desmoid tumors was higher in patients undergoing total proctocolectomy with ileal pouch anal canal anastomosis (IPAA) than in patients undergoing total colectomy with ileorectal anastomosis (IRA) [58, 67].
- The mortality rate with the development of desmoid tumors is 0–14% [56, 60, 62, 63, 68].
- Previous studies on the correlation between desmoid tumor development and pathogenic variants of the *APC* gene indicated that pathogenic variants in the 3′ side or codon 1445–1580 were more correlated to onset than in codon 1444 of *APC* [69, 70], but Church et al. indicated a higher incidence in pathogenic variants in the 3′ side than in codon 1399, and although symptoms tended to be stronger and more fatal, there were no correlations between the variant sites of *APC* and development of desmoid tumors [71].

## [Nonsurgical treatment]

- Medications should be considered for large or rapidly growing intra-abdominal or abdominal-wall desmoid tumors (Side Memo 5: Drug therapy for desmoid tumors).
- Radiation therapy is generally not recommended because it can cause bowel injury and is poorly effective [72].

Side memo 5

- ■ Drug therapy for desmoid tumors

[NSAIDs and antiestrogens]

There have been studies on the safety and efficacy of treatment with sulindac (300 mg/day), which is one of the non-steroidal anti-inflammatory drugs (NSAIDs); tamoxifen (40–120 mg/day) and raloxifene (120–240 mg/day), which are antiestrogens; and toremifene (180 mg/day), as well as combinations of NSAIDs and antiestrogens [73–75]. Both sulindac and antiestrogens have a limited effect in reducing the tumor size but suppress tumor growth, although long-term observations are needed since studies have indicated that an average of 15 months elapsed before effects were observed [75–78].

[Molecular targeted drugs]

Recently, the efficacy of a tyrosine kinase inhibitor, imatinib, has also been examined. Desurmont et al. [77] reported that imatinib reduced the tumor size or stabilized the tumor size in 36% of treated cases. In contrast, Chugh et al. [79] reported a 1-year progression-free rate of 66% in patients with inoperable desmoid tumor treated with imatinib, but the tumor size was reduced in only 3% of the patients. Therefore, presently, the efficacy of imatinib remains to be clearly established.

[Cytotoxic chemotherapy]

Regarding cytotoxic chemotherapy, high response rates were reported with a combination regimen of doxorubicin (DOX) plus dacarbazine (DTIC) [80]. In Japan, DOX + DTIC therapy has also been found to be effective, at the same time, its toxicity is concerned [81–83]. In addition to DOX + DTIC therapy, the efficacy of methotrexate (MTX) and vinblastine (VBL) has been reported [80].

[Effects according to treatment type]

Desurmont et al. [77] compared the response rates of intra-abdominal desmoid tumors to various pharmacotherapies. They found that the response rates were 77% to treatment with cytotoxic chemotherapy, 50% to treatment with sulindac + tamoxifen, 40% to treatment with tamoxifen, 36% to treatment with imatinib, and 28% to treatment with sulindac. Thus, the response rate of intra-abdominal desmoid tumors was the highest with treatment with cytotoxic chemotherapy, and they concluded that cytotoxic chemotherapy could be the first-line treatment. However, sufficient studies have not been conducted that the first-line cytotoxic chemotherapy should be done for which type of intra-abdominal desmoid tumors (there is only retrospective study or the single-center empirically data so far), so there is currently no standard treatment [84].

## [Surgical treatment]

- Extra-abdominal desmoid tumors show high recurrence rates after resection (20–25%), although the incidence of postoperative complications is low.
- Because recurrence after resection may be caused not only by incomplete resection but also possibly by new tumor development at the site of incision, excessive peritumoral resection should be avoided [85].
- Surgical treatment of asymptomatic intra-abdominal desmoid tumors is discouraged (CQ4).
- Although surgery should be considered for bowel obstruction due to intra-abdominal desmoid tumors, it may not be successful due to the difficulty of resection or necessity for massive intestinal resection [78].
- A previous study has indicated the absence of any difference in survival between patients treated by complete resection and patients not treated by complete resection [86].
- Data from Japan showed that surgical resections were conducted in extra-abdominal, intra-abdominal, and mixed treatments in 86, 48, and 71% of cases, respectively [83].

## [Treatment of intra-abdominal desmoid tumors based on the Church classification]

**Fig. 15:**
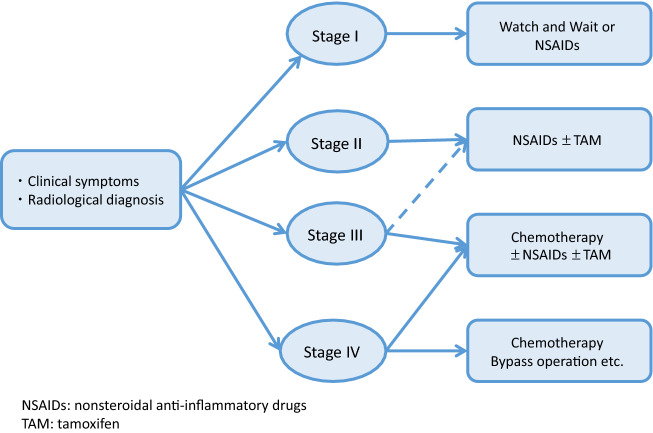
Disease classification and treatment plans for intra-abdominal desmoid tumors

- A staging system for intra-abdominal desmoid tumors developed based on the classification of Church et al. [68] is shown in Table 10.
- An analysis of 26 patients in Japan showed that 11, 8, and 7 patients were at stages I, III, and IV, respectively, with those at stage II having symptoms < 10 cm being rare. Nonsurgical treatment, such as the use of NSAIDs (mainly sulindac) and chemotherapy, was more prevalent at stage I. Moreover, 62.5% of patients at stage III underwent surgery, and administration of NSAIDs (mainly sulindac), hormone therapy, and chemotherapy were conducted at high frequency.
- No prospective studies have been conducted, but options include follow-up observations of NSAIDs for stage I, surgery and NSAIDs + tamoxifen if possible for stage II, chemotherapy ± NSAIDs ± tamoxifen for stage III, and chemotherapy or bypass surgery for stage IV. Sulindac is mainly used as NSAID. No deaths were reported for stage I/II, and mortality rates for stage III and IV were 15 and 44%, respectively (Fig. 15). Stenting is recommended for uretic obstruction [68, 87].

### Other extracolonic concomitant lesions

**Fig. 16:**
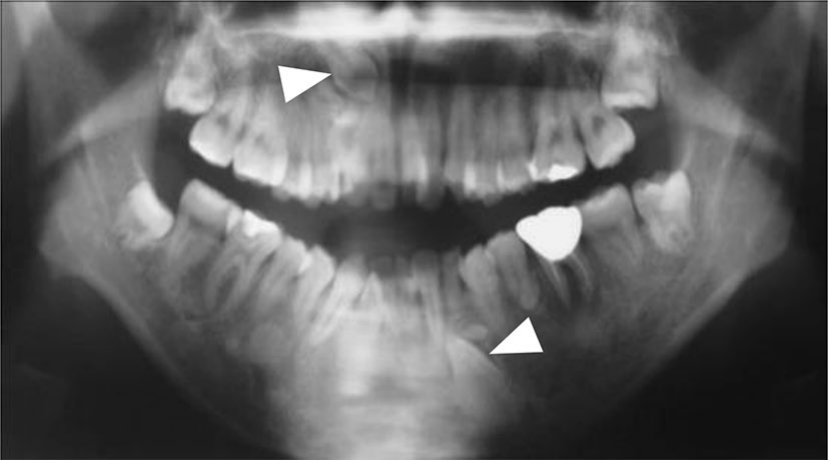
Dental abnormalities (unerupted teeth)

**Fig. 17:**
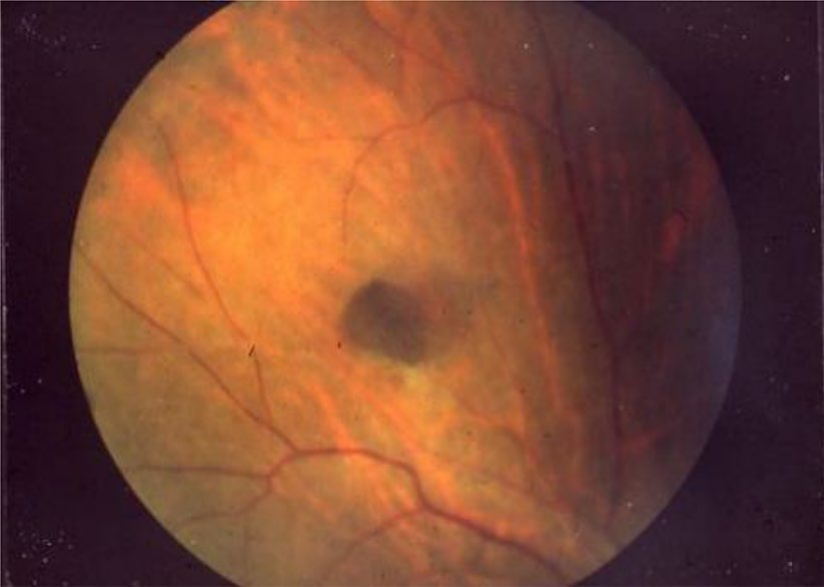
Congenital hypertrophy of the retinal pigment epithelium

- Neoplastic lesions, such as subcutaneous soft tissue tumors or osteomas and dental abnormalities (Fig. 16), is a useful clue for the clinical diagnosis for FAP.
- Congenital hypertrophy of the retinal pigment epithelium (CHRPE) (Fig. 17), a nontumorous lesion, is detectable before the development of colorectal adenomas in patients with FAP and can be used as a supplementary diagnosis for FAP (Side Memo 6: CHRPE).

**Table 10:** Staging system for intra-abdominal desmoid tumors based on Church’s classification [68]

	I	II	III	IV
Size	< 10 cm		10–20 cm	> 20 cm
Growth speed	No growth for 6 months		Present	Present^a^
Uretic obstruction	No		Yes	
Bowel obstruction	No		Yes	
Sensation of tumor	No	Yes		
Pain	No	Yes		
Restriction of daily life	No		Yes	
Hospitalization	Unnecessary			Necessary

^a^Maximal diameter increase of ≥ 50% within 6 months

Other tumorous lesions in addition to desmoid tumors include thyroid cancer, adrenal tumor, hepatoblastoma, and brain tumors (Side Memo 6: Turcot syndrome).

Side Memo 6

- ■ CHRPE

CHRPE is a discontinuous flat pigmented lesion of the retina without clinical symptoms, which does not require any treatment. It does not affect visual acuity and does not show malignant transformation. Because approximately 80% of patients with FAP have CHRPE and it can be detected at birth, it can serve as a supplementary diagnosis in children.

- ■ Turcot syndrome (type 2)

Colorectal polyposis associated with brain tumors (mainly cerebellar medulloblastoma) and with a germline variant in the *APC* gene is called Turcot syndrome, type 2 (see Lynch syndrome for Turcot syndrome, type 1).

## Surveillance and treatment

### Surveillance of colorectal adenomas

- Colonoscopy surveillance of the lower digestive tract for patients with FAP is strongly recommended at an interval of 1–2 years after the age of 10 years for patients with typical FAP and 2–3 years after the age of 10 years (18–20 years) for patients with AFAP.

#### Comments

- The age at which surveillance of the lower digestive tract is the same for both genetically diagnosed and genetically untested patients with FAP.
- An analysis by a European group of colorectal cancer development in patients with FAP aged ≤ 20 years showed that colorectal cancer development was not observed before the age of 10 years and was observed in 0.2% of patients between the ages of 11 and 15 years [12]. Therefore, the recommended age for initiation of colonoscopy surveillance in patients with FAP is after the age of > 10 years [25]. However, caution should be exercised in patients with severe FAP because of the possible development of colorectal cancers before the age of 10 years [88].
- The age at which colorectal cancers develop in AFAP is 10–15 years later than in patients with typical FAP [89], and the development of colorectal cancers is rare in those aged < 30 years [90], so colonoscopy surveillance begin in the patient’s late teenage years (18–20 years) [25].
- Patients with FAP can be endoscopically denied as a possibility in patients who have not undergone genetic testing, if no colorectal adenoma is found by the age of 35 years.
- An interval of 1–2 years (2–3 years for patients with AFAP) is generally recommended for colonoscopy surveillance.

## Treatment of colorectal adenomas

**Fig. 18:**
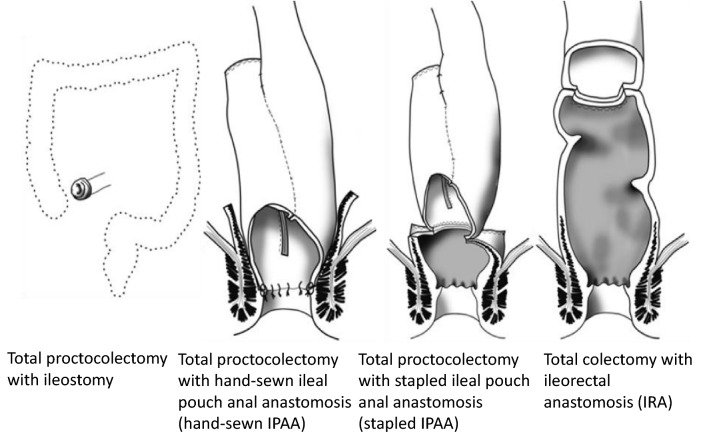
Surgical procedures for patients with FAP (Side Memo 7: nomenclature of operative procedures)

- The definitive treatment is proctocolectomy (prophylactic proctocolectomy) prior to the development of colorectal cancer (CQ5).
- The main surgical procedures include the following (Side Memo 7: Nomenclature of surgical procedures) (Fig. 18, Table 11):

**Table 11:** Characteristics of surgical procedures for patients with FAP

Surgical procedures	Total proctocolectomy with permanent ileostomy (TPC)	Total proctocolectomy with ileal-pouch anal anastomosis (IPAA)	Total colectomy with ileorectal anastomosis (IRA)
Advantages	Colorectal cancer completely prevented	Colorectal cancer almost prevented	Good bowel function
		Preservation of natural anal function	Relatively easy surgical technique
			Lower complication rate than IPAA
Disadvantages	Deteriorated body image with permanent colostomy	A complex surgical technique	Possibility of development of rectal cancer (depending on the number of adenomas, location of the germline variant in the *APC* gene, or length of the residual rectum)
		Unstable bowel function	
		Possibility of cancer development at the remaining rectal mucosa near the anus	
		Possibility of pouchitis	

Total proctocolectomy (TPC) + permanent ileostomy.

Total proctocolectomy + ileal pouch-anal anastomosis (IPAA).

Total colectomy + ileorectal anastomosis (IRA).

IPAA is currently considered the standard surgical procedure and has a high implementation rate [91–94] (CQ5).

It is generally recommended that patients undergo surgery in their 20 s.

## Comments

### [Surgery]

- Recently, laparoscopic surgery has been increasingly used for prophylactic proctocolectomy (CQ7).
- In cases where desmoid tumors are found in the mesentery during prophylactic surgery, IPAA is generally not recommended, owing to the risk of recurrence or enlargement of desmoid tumors and technical problems, but it may be acceptable under certain conditions (Chapter II 3–3: Desmoid tumors).
- Total proctocolectomy for patients with FAP may reduce fertility in women (Side Memo 8: Surgery and fertility, pregnancy, and delivery).
- Drug therapy with NSAIDs has been attempted; however, its usefulness is unclear (CQ8).

Side Memo 7 **■ Nomenclature of operative procedures**

In Western countries, ileal pouch anal anastomosis with mucosectomy (IAA) and ileal pouch anal canal anastomosis (IACA) are often collectively called ileal pouch-anal anastomosis (IPAA), without discrimination between them. In addition, IAA is sometimes called handsewn IPAA, and IACA is sometimes called stapled IPAA. The height of the anastomosis (length of the residual rectum) is not clearly defined for ileorectal anastomosis (IRA). Total proctocolectomy + permanent ileostomy is often called total proctocolectomy (TPC).

Side Memo 8 **■ Surgery and fertility, pregnancy, and childbirth**

A study of 58 Danish female patients with FAP [95] showed that fertility was 90%, which was comparable to the general population. In a study on 162 European female patients with FAP, the fertility of patients with FAP who had not undergone surgery was comparable to the general population. The fertility of patients who had undergone IRA was also comparable to that of the general population, but the fertility rate in patients who had undergone IPAA decreased by a factor of 0.46 [96]. In contrast, a study of 138 Dutch patients with FAP reported that fertility was unrelated to the surgical procedure and associated with the age at the first surgery [97]. Postoperative adhesions are considered a possible cause of reduced fertility after IPAA. Oresland et al. [98] conducted hysterography after total proctocolectomy and reported adhesion of the fallopian tubes to the pelvic walls, unilateral obstruction, and bilateral obstruction in 48, 43, and 10% of patients, respectively. A study including patients with FAP and ulcerative colitis reported that laparoscopic IPAA was associated with significantly higher fertility than open IPAA [99]. However, there have been no prospective trials on patients with FAP. Studies including patients with FAP and ulcerative colitis reported that pregnancy/vaginal delivery after IPAA was safe [100, 101]. However, injury to the anal sphincter and pelvic nerves after episiotomy must be considered in vaginal deliveries after IPAA.

## [Timing of surgery for prophylactic total proctocolectomy]

- The most important considerations in deciding the timing of prophylactic proctocolectomy in patients with FAP are as follows: (1) cumulative prevalence of colorectal cancer [22]; (2) density of the adenomas [102]; (3) size and morphology of the adenomas; (4) age at death, age at cancer onset, and presence/absence of desmoid tumors in members of the pedigree [59]; (5) germline variant site in the *APC* gene [103]; (6) educational, work, and other environments of the patient [104]; (7) fertility [96] and presence/absence of male sexual dysfunction [105] after IPAA; (8) presence/absence of gastrointestinal symptoms, such as diarrhea, abdominal pain, and melena; and (9) histopathological findings of the tumor.
- Considering the prevalence of colorectal cancer, it is recommended that some patients with classical FAP should undergo surgery while in their teens and that most patients with FAP should undergo surgery while they are in their 20s [106, 107].
- Recently, the International Society for Gastrointestinal Hereditary Tumors (InSiGHT) established a staging system based on endoscopic assessments that use the numbers and sizes (polyp burden) of colorectal adenomas (Table 12), showing that the interval between colonoscopy and surgical indication decision was strongly correlated with polyp burden [108]. Clear increases in polyp burden observed over time were also used as criteria for clinical indications for surgical indication [109]. Although the polyp burden match rate with assessors cannot be sufficient, polyp burden determines the intervals of colonoscopy and surgical indication decisions, and surgery should be considered when these clearly increase.

## Treatment of colorectal cancer

- In patients with FAP with locally advanced colorectal cancer, standard treatment for locally advanced colorectal cancer should be performed. If curative resection of colorectal cancer can be expected, the surgical procedure should be selected according to the condition of the patients with FAP.

**Table 12:** InSiGHT polyposis staging system [108]

Site	Stage	Number of polyps	Size	Number of polyps with size > 1 cm	Severe dysplasia or invasive carcinoma	Endoscopic resection^b^
			(maximum)			
Colon	Stage 0	< 20	< 5 mm	0	None	Suitable
	Stage 1	20–200	Irrespective			
	Stage 2	200–500		< 10		
	Stage 3	500–1000		10–50 cells		
	Stage 4	≥ 1000		Irrespective	Present	Unsuitable
Rectum	Stage 0	< 10	< 5 mm	0	None	Suitable
	Stage 1	10–25	Irrespective			
	Stage 2			Irrespective		
	Stage 3^a^	> 25				
	Stage 4^a^				Present	Unsuitable

^a^Independent of complete resection for serrated polyps

^b^Endoscopic resection (polypectomy/endoscopic mucosal resection)

## Comments

- In patients with FAP with locally advanced colorectal cancer, the surgical procedure should be determined after a comprehensive consideration of the stage and site of colorectal cancer. If curative resection of the colorectal cancer can be expected, IPAA or IRA with dissection of the regional lymph nodes is an option; in contrast, if the colorectal cancer cannot be curatively resected, a surgical procedure such as that for sporadic colorectal cancer should be selected.
- Chemotherapy similar to that used for patients with sporadic colorectal cancer should be used for colorectal cancer associated with FAP.
- Even after IPAA or IRA, chemotherapy selection can be guided by the recommendations in the “JSCCR guidelines for the Treatment of Colorectal Cancer” [1].
- If metastatic lesions can be curatively resected, treatment similar to that for metastases from sporadic colorectal cancer should be used.

### Examinations for extracolonic manifestations before proctocolectomy

- It is desirable to conduct extensive examinations to check for extracolonic manifestations before proctocolectomy, irrespective of the presence or absence of associated locally advanced colorectal cancer, although there is little evidence of its usefulness.
- It is recommended to check for the presence of gastroduodenal lesions and desmoid tumors before proctocolectomy (CQ2).
- Examinations for other tumorous lesions can be performed during the surveillance after proctocolectomy (CQ9).

## Comments

- The presence or absence of adenomas and cancers of the stomach and duodenum, including that of the ampulla of Vater, should be determined by upper gastrointestinal endoscopy.
- The presence or absence of desmoid tumors should be checked by palpation, computed tomography (CT), and/or magnetic resonance imaging (MRI).
- Ultrasonography to check for thyroid cancer need not necessarily be performed before colectomy but must be incorporated into the postoperative surveillance plan, especially in female patients.
- Generally, small-bowel follow-through and small-bowel endoscopy (capsule endoscopy) are not performed before proctocolectomy, except when there are symptoms/findings (including preoperative diagnostic imaging findings), raising the suspicion of intestinal lesions (CQ10).
- Because adrenal tumors develop at a low frequency and hepatoblastomas and brain tumors develop commonly only until 2–3 years of age and up to adolescence, respectively, preoperative examinations for these tumorous lesions are generally not required.

## Surveillance after proctocolectomy

- If there is any residual colorectal mucosa after prophylactic proctocolectomy, regular colonoscopic examination is required, in view of the possibility of new colorectal cancer development.
- In patients with FAP undergoing surgery for colorectal cancer, postoperative surveillance similar to that in patients with sporadic colorectal cancer should be planned or performed.

## Comments

- Long-term surveillance to monitor the development of cancer in the remaining rectum is required after IRA (Side Memo 9: Risk of rectal cancer development after total colectomy + IRA).
- Usually 2–3 cm of rectal mucosa is left behind after stapled IPAA, and a small amount of rectal mucosa may also be left behind after hand-sewn IPAA. Therefore, long-term surveillance of the remaining rectum is required after stapled IPAA and hand-sewn IPAA.
- Adenomas in the ileal pouch develop in 6.7–74% of patients after IPAA [56, 110–112], and cancer also develops [113, 114]. Therefore, long-term surveillance is necessary.
- Pouchitis develops in approximately 5% of patients undergoing IPAA for patients with FAP, but the incidence is lower than that after surgery for ulcerative colitis [115]. The condition usually manifests with fever, diarrhea, and anemia, and if these symptoms are noted, colonoscopic examination should be performed immediately.
- In patients with FAP with advanced colorectal cancer treated by curative resection, surveillance for recurrence should be performed as that in patients with sporadic colorectal cancer.

Side Memo 9 ■ Risk of rectal cancer development after total colectomy + IRA.

Long-term follow-up after IRA revealed that 24–43% of patients develop cancer in the remaining rectum [116, 117]. During a 20-year period after IRA, the rectum had to be resected in 10% of patients with AFAP, 39% of patients with sparse FAP, and 61% of patients with severe FAP [118]. With advances in surgical techniques, IPAA has been used in an increasing proportion of cases [91–93], and the use of IPAA in patients with a greater number of risk factors for rectal cancer has reduced the proportion of patients undergoing proctectomy after IRA from 40 to 13% and also reduced the cumulative incidence of cancer development in the remaining rectum after IRA [119–121].

## Surveillance for extracolonic manifestations

- Surveillance should be conducted considering the possible development of desmoid tumors, which tend to develop within 2–3 years after colectomy, and malignancies, such as duodenal cancers.

## Comments

- Extracolonic manifestations requiring treatment often develop after proctocolectomy (colectomy). A method for surveillance of the remaining rectum and extracolonic manifestations after proctocolectomy (colectomy) is proposed, as shown in Table 13 [13].
  [Gastrointestinal tract]
- Polyps in fundic gland polyposis usually show the histological features of hyperplastic polyps and, therefore, do not constitute an indication for surgery. Gastric adenomas develop mainly in the antrum. In Japan, patients with FAP are at higher risk of developing gastric cancer than the general population. Surveillance of gastric lesion should be conducted simultaneously with duodenal surveillance (CQ2).
- The incidence of cancer in the duodenum (including the ampullary region) is high, necessitating regular endoscopic follow-up and treatment of adenomas (CQ2).
- No recommended method for surveillance of the ileum/jejunum has been established yet. Jejunal/ileal cancer rarely develops (CQ10).

## [Desmoid tumor]

- Desmoid tumors often develop in the abdominal wall, mesentery, or retroperitoneum within 2–3 years after proctocolectomy (colectomy) [56, 57]. Palpation and diagnostic imaging should be performed carefully, and careful attention should be paid to the clinical symptoms (abdominal pain, abdominal distension, mass, gastrointestinal obstruction, etc.).

## [Others]

- Among malignancies, attention should be paid to the development of thyroid cancer (especially in women). Neck palpation and ultrasonography should be performed once a year (CQ9).

## Management of families (relatives)

- It is desirable to provide genetic counseling to not only patients but also their relatives.
- Surveillance of the gastrointestinal tract, mainly of the large intestine, should be performed in first-degree relatives (parents, children, and siblings) after obtaining informed consent.

**Table 13:** Surveillance of the remaining rectum and major associated lesions in patients with FAP after surgery

Associated lesions	Initiation age and screening procedures
Remaining rectal adenoma	Annual colonoscopy with polypectomy or ablation after IPAA
	Colonoscopy with polypectomy or ablation every 6 months (depending on age or density of adenoma) in patients who underwent IRA
Duodenal adenoma or cancer (including ampullary lesions)	Baseline upper gastrointestinal endoscopy is initiated at the time of colectomy or at 20–25 years of age, whichever is earlier. Thereafter, upper gastrointestinal endoscopy is repeated regularly depending on the severity
Gastric adenoma or cancer	Upper gastrointestinal endoscopy annually (or simultaneously with examination for duodenal lesions)
Thyroid cancer (for women)	Thyroid ultrasonography and palpation annually starting in the late teenage years
Intra-abdominal desmoid tumor	Abdominal palpation annually. After colectomy, abdominal and pelvic CT or MRI every 3 years in patients with a family history of desmoid tumors
Brain tumor	Annual examination
Jejunal ileal adenoma or cancer	Data to support any recommendation are lacking. Simultaneously performed with radiological examinations (CT/MRI) for desmoid tumors as much as possible

Modification with ref. [13]

## Comments

## [Genetic counseling]

**Fig. 19:**
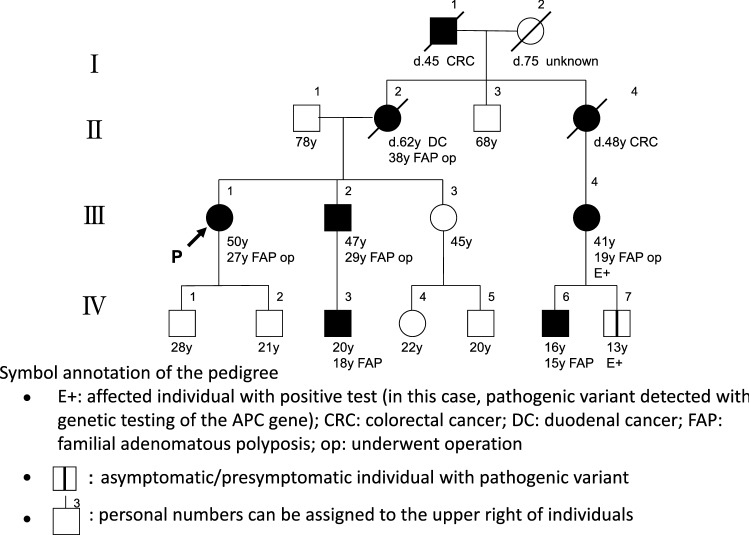
Example of description of pedigree for FAP (Appendix: Principles for writing and reading pedigrees for an outline of description method). Symbol annotation of the pedigree. E+: affected individual with positive test (in this case, pathogenic variant detected with genetic testing of the *APC* gene).  : asymptomatic/presymptomatic individual with pathogenic variant.  : personal numbers can be assigned to the upper right of individuals

- It is indispensable to obtain a family history in patients with hereditary cancer syndrome, including FAP, and desirable to accurately describe/record the family history using a pedigree chart [122, 123] (Fig. 19).

[Response to first-degree relatives]

- If any relatives have colorectal adenomas (particularly two or more colorectal adenomas), the FAP diagnostic chart (Chapter I Fig. 5: Flowchart of hereditary colorectal polyposis diagnosis) should be followed.
- If no adenomas are detected by colonoscopy, colonoscopy surveillance should be performed approximately every 3 years.
- If no adenomas are detected by several colonoscopy surveillances up to 35 years of age, FAP can almost definitely be ruled out.

## [Genetic diagnosis of relatives]

- If genetic testing is performed, genetic counseling needs to be provided by a physician and/or specialist (clinical geneticist or genetic counselor) before and after the genetic testing.
- If a germline variant for the *APC* gene was detected in the family member, then a single site analysis is available with genetic testing using blood samples.

## [FAP in childhood]

- Lesions in the gastrointestinal tract
  - Multiple adenomas appear in the large intestine in teenagers. Many adenomas are asymptomatic, while some have gastrointestinal symptoms, such as bloody stools, anemia, diarrhea, and mucus in the stool [124]. Colorectal cancer in patients aged < 20 years are extremely rare (< 0.2% of patients with FAP) [12]. However, colonoscopy should be performed regardless of age if gastrointestinal symptoms are present.
  - If FAP is diagnosed, colonoscopy should be conducted for colorectal lesions from the age of 12–14 years every 1–3 years until prophylactic proctocolectomy is conducted [124–126].
  - For lesions in the upper gastrointestinal tract, polyps can be found even during childhood, but there have been no studies on gastric or duodenal cancers, and in principle, upper gastrointestinal endoscopy should be initiated in adulthood [124–126].

- Lesions outside of the gastrointestinal tract
  - Hepatoblastoma: The frequency of hepatoblastoma, which mostly develops until 3 years of age, is less than 2% in children with FAP. There are opinions on the recommendation of hepatoblastoma screening tests (abdominal ultrasonography and serum α-fetoprotein every 4–6 months) [125, 126], and some cannot recommend them due to a lack of evidence on its efficacy [124]. Parents of infants with a family history of FAP should be provided with explanations on the risks of hepatoblastoma and individually handled with consideration of their desires.
  - Thyroid cancer: Although thyroid cancer can develop in childhood alongside FAP, surveillance has not been established as in adults.
  - Desmoid Tumors: There have been studies on intra-abdominal desmoid tumors after proctocolectomy even in childhood.

- CHRPE: CHRPE is a discontinuous flat pigmented lesion of the retina that is present in approximately 80% of patients with FAP since birth. There are no clinical symptoms and no need for treatment. Visual acuity is not affected, and it does not show malignant transformation. Bilateral or multiple CHRPE is a characteristic finding in patients with FAP complications.

Side Memo 10 **■ Genetic testing for children**

Genetic testing for the diagnosis of FAP in asymptomatic children with a family history of FAP should be carefully addressed. The “Guidelines for Genetic Tests and Diagnoses in Medical Practice” by the Japanese Association of Medical Sciences (February 2011) states that it is necessary to obtain the consent of an individual standing as a surrogate representative when genetic testing of diseases that develop before adulthood if their pre-symptomatic diagnoses are useful in the management of the examinee’s healthcare. In this case, the surrogate should decide after a thoughtful consideration of the examinee’s beneficence in his/her health care. It is desirable to obtain an assent from the examinee after giving the explanation of the test at a level corresponding to the patient’s ability. Genetic counseling is needed to ensure that the patient understands the advantages and disadvantages in terms of medical care and psychological, social, and economic aspects when establishing a diagnosis of FAP and participates in decision-making. Furthermore, genetic counseling is conducted even after confirmation of diagnosis, and a continuous support system is provided.

## Clinical questions

### **CQ-1**: Is genetic testing recommended in patients with FAP?

Genetic testing in patients with clinically diagnosed FAP is weakly recommended for treatment selection and surveillance reference and differentiation from other types of adenomatous polyposis (Recommendation 2/Evidence level C).

## Comments

### Genetic testing for patients with clinically diagnosed FAP

FAP can often be diagnosed clinically and do not always require genetic testing. Between 20 and 25% of patients develop FAP *de novo* (i.e., variant occurrence as a proband), so they may have pathogenic variants in the *APC* gene even without a family history [127]. Furthermore, 20% of patients with FAP occurring *de novo* exhibited *APC* mosaicism (Chapter II 2-3: Diseases and pathological conditions requiring differentiation) [31]. Numerous studies to date have reported a correlation between locations (genotype) of *APC* gene variants and number of colorectal adenomas and concomitance of extracolonic lesions (phenotype) [27, 28, 118, 128]. Therefore, the genotype may serve as a reference for the timing of proctocolectomy, selection of surgical procedure, prediction of postoperative desmoid tumor development, and concomitant lesion surveillance.

### Diagnosis of AFAP or differential diagnosis of MAP or PPAP

Although AFAP can often be clinically diagnosed based on the number of polyps in the colorectum (< 100) and a family history consistent with autosomal dominant inheritance, extracolonic manifestations (e.g., fundic gland polyposis, duodenal adenoma, osteoma, desmoid tumor, CHRPE), etc., identification of a pathogenic germline variant in the *APC* gene is useful for a definitive diagnosis.

If only the patient or only the sibling(s) of the patient among the family members have < 100 colorectal adenomas, the patient or siblings may have MAP with an autosomal recessive mode of inheritance, and genetic testing of the *APC* gene, followed by, or simultaneously with, genetic testing of the *MUTYH* gene is useful in the differential diagnosis between the two diseases.

PPAP is a disease with an autosomal dominant mode of inheritance caused by a pathogenic germline variant of the *POLE* or *POLD1* genes, and the number of colorectal polyps is often < 100 [37, 129]. Studies have indicated that tumorous lesions other than colorectal cancer include duodenal adenomas or central nervous system tumors in *POLE* gene variants and endometrial cancer, breast cancer, and central nervous system tumors in *POLD1* gene variants [38, 39].

No pathogenic germline variants of the *APC* gene are identified in some patients who have been clinically diagnosed with FAP. According to a study from Western countries [9], pathogenic germline variants of the *APC* gene are identified by usual testing methods in approximately 60% of patients with classical (typical) FAP, and pathogenic *APC* germline variants and biallelic mutations of the *MUTYH* gene are identified in 10 and 7% of patients with 20–99 colorectal adenomas, respectively, and 5 and 4% of patients with 10–19 colorectal adenomas, respectively [20]. The possible reasons for the failure to detect pathogenic germline variants of the *APC* gene include (1) difficulty in the detection of *APC* gene alterations by the analysis method used, (2) presence of unknown causative genes for adenomatous polyposis, (3) *APC* mosaicism, (4) MAP, and (5) PPAP.

## **CQ-2**: Is upper gastrointestinal surveillance recommended in patients with FAP?

Upper gastrointestinal surveillance in patients with FAP is strongly recommended because of the increased risk of gastric and duodenal cancers (including ampullary cancers) compared with that in the general population (Recommendation 1/Evidence level C).

## Comments

Upper gastrointestinal endoscopy for patients with FAP is recommended at the initial diagnosis to confirm the presence of fundic gland polyposis and identify duodenal and ampullary adenomas. In Japan, determination of the optimal surveillance interval requires analysis of the incidence and mortality rates of gastric cancer, correlations with other organ disease types, and associations with background factors, such as family history. The incidence rates of fundic gland polyposis and gastric adenomas in patients with FAP in Japan have been reported to be 50.2–64% and 14.7–39%, respectively [130, 131, 149]. *Helicobacter pylori* (*H. Pylori*)infection and gastric mucosal atrophy tend to be minimal when fundic gland polyposis is present, but associations with the sites of *APC* gene variants and family history have been understudied [131, 132]. Studies in Japan have indicated that the prevalence rate of gastric cancer in patients with FAP was 3.1–4.2% [49, 60], but studies in Western countries indicated that the prevalence rate was low at 0.6% [46]. Studies in Japan on gastric cancer in patients with FAP are more diverse than the general population, including gastric cancer caused by fundic gland polyposis, gastric cancer caused by gastric mucosal atrophy due to *H. Pylori* infection, and coexistence high-grade adenoma/ dysplasia [44, 133, 134], and there are many cases of early detection with periodic endoscopic surveillance [130, 133, 134]. Meanwhile, gastric cancers that develop in GAPPS, which is a polyposis limited to the stomach due to pathogenic germline variants in the promoter 1B of the *APC* gene, require differentiation at the time of initial diagnosis due to its high malignancy [135].

The incidence rates of duodenal adenoma and duodenal cancer in patients with FAP have been reported as 39.2 and 7.7%, respectively [49], and duodenal cancer is thought to be an extracolonic complication that can be a prognosticator. There is no consensus on the necessity, timing, or methods of aggressive treatment for duodenal adenomas, but this is a topic of future research. The incidence of ampullary adenoma complications in patients with FAP is 52%, and slow growth of the adenoma due to long-term follow-up has been indicated, but tumorigenesis is rare at 1%. Therefore, several studies have proposed that aggressive treatment against this adenoma is unnecessary [136, 137].

To date, no prospective study has investigated the efficacy of surveillance of the upper gastrointestinal tract in patients with FAP, but we recommend that surveillance of the upper gastrointestinal tract be performed in patients with FAP because of the higher risk of gastric and duodenal cancers (including ampullary cancers) compared with the general population.

## **CQ-3**: Is pancreas-sparing duodenectomy (PSD) for duodenal adenomas (excluding the ampullary region) recommended in patients with FAP?

PSD for duodenal adenomas is weakly recommended in patients with FAP (Recommendation 2/Evidence level C).

## Comments

Surgery should be considered for duodenal adenomas (excluding the periampullary region) with a diagnosis of stage IV or a higher Spigelman classification among patients with FAP. Surgical procedures include pancreaticoduodenectomy (PD), pylorus-preserving pancreaticoduodenectomy (PPPD), or pancreas-sparing duodenectomy (PSD). In particular, surgical procedures where the entire duodenum is resected without preserving the pyloric ring may be referred to as pancreas-sparing total duodenectomy (PSTD).

PD and PPPD are generally preferred as surgical procedures, but recently, PSD is increasingly being reported as well.

PSD has been performed in 13 Danish patients with FAP between 1999 and 2010, resulting in postoperative complications in six patients (46%) including three cases of anastomotic leakage; nevertheless, their conditions improved through conservative treatments [138]. According to a Dutch multicenter retrospective study, PSD was used in 51% (22 of 43 patients) of patients with FAP who underwent duodenectomy for duodenal adenomas, with the frequency of postoperative complications being equivalent to that of PD and PPD, hence making PSD the most adopted surgical procedure for “prophylactic duodenectomy” since 1999 [139].　In Japan, there is one single-center retrospective study regarding PSD (PSTD) performed in 10 patients with duodenal adenomas diagnosed with stage IV Spigelman classification. In terms of postoperative complications, four patients had pancreatic fistulas that have been managed through conservative treatment, and one patient had surgical site infection and cholangitis with low severity grade, suggesting that PSD (PSTD) is a feasible procedure [140].

In a summary of the studies on PSD between 1995 and 2012, 20% of all 96 patients underwent total duodenectomy, and 80% preserved their pyloric rings [140]. Whether or not total duodenectomy is necessary remains controversial, since there is no definite conclusion regarding the risk of carcinogenesis from the remnant duodenal mucosa after PSD with preservation of the pyloric ring.

Propensity score-matched analysis indicated that compared with PD, PSD was associated with a shorter operative time (391 vs. 460 min) and less frequent exocrine pancreatic insufficiency (11 vs. 30%). Although the incidence of late acute pancreatitis was more common after PSD (16 vs. 0%), there was no difference in any of the early postoperative complications, suggesting that PSD is a reasonable option for duodenal cancer prophylaxis in patients with FAP having high-risk features [141].

Despite the preoperative diagnosis of duodenal adenomas, postoperative histological examinations reveal duodenal carcinoma in 9.6–30% of cases [140, 142]. Therefore, it is crucial to perform preoperative assessments through careful observations of the duodenum and to select the appropriate surgical procedure for duodenal adenoma management among FAP patients with advanced Spigelman stages.

## **CQ-4**: Is surgical treatment for asymptomatic intra-abdominal desmoid tumors recommended in patients with FAP?

Surgical treatment for asymptomatic intra-abdominal desmoid tumors in patients with FAP is not strongly recommended because of the high risk of recurrence after resection and the possibility of spontaneous resolution (Recommendation 1/Evidence level D).

## Comments

The clinical characteristics of desmoid tumors among FAP patients vary from those of sporadic desmoid tumors, and this should be emphasized during desmoid tumor management. In Japan, surgical resection was performed in 86, 48, and 71% of patients with extra-abdominal, intra-abdominal, and mixed desmoid tumors, respectively [83].

Intra-abdominal desmoid tumors often develop in the mesentery after proctocolectomy [56–58], and a massive resection of the intestine may become necessary during surgical resection [76]. A study on surgical treatment relating to intra-abdominal desmoid tumors indicated that there was no difference in survival rate between cases of complete resection and non-resection, including intestinal bypass surgery cases [86]. Intra-abdominal desmoid tumors should be managed carefully by considering their characteristics, including: (1) the recurrence that has been reported to occur in 10–68% of cases despite complete resection [61], (2) the spontaneous decrease of size or resolution in 5–33% of cases [63–65], and (3) the invasive nature of the surgery [78]. Surgical interventions for intra-abdominal desmoid tumors should be limited to cases with the symptom of the bowel obstruction [143], whereas for those without symptoms, conservative treatment (follow-up observations) [144] and/or pharmacotherapy is recommended based on the Church classification [68, 71].

Regarding the management of extra-abdominal (mostly abdominal wall) desmoid tumors, conservative treatment should be conducted. Nevertheless, surgery may be considered when quality of life is affected, such as during instances of motion restriction. Although recurrence rates after extra-abdominal desmoid tumor resection are relatively high (20–25%), postoperative complications are rarely recognized. Excessive resection at the tumor margin should be restricted even when the surgery is aiming for complete tumor removal since desmoid tumor recurrence happens not only from the residual tumor but also by the de novo development in the resection wound area [85].

## **CQ-5**: Is prophylactic total colectomy recommended in patients with FAP?

Prophylactic total proctocolectomy with IPAA in patients with typical FAP is strongly recommended (Recommendation 1/Evidence level C).

## Comments

**Fig. 20:**
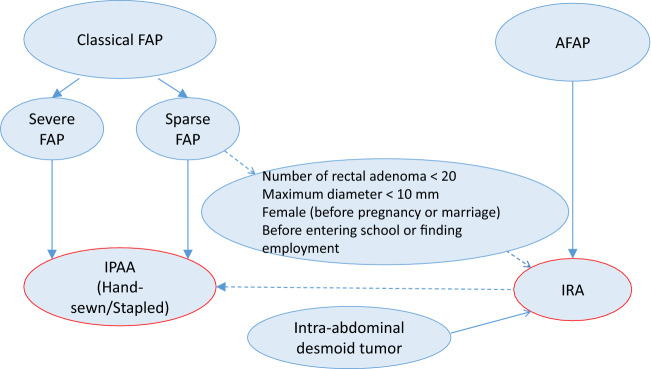
Flowchart for prophylactic surgical procedure in patients with FAP

IPAA is the standard procedure for patients with classical FAP [92] (Fig. 20), and it generally involves the construction of an ileal pouch in a J-shape [145]. Moreover, IPAA is largely classified into two procedures: (1) hand-sewn IPAA, wherein the rectal mucosa is dissected from the dentate line and an ileal pouch is manually anastomosed to the dentate line and (2) stapled IPAA, wherein stapling anastomosis of the surgical anal canal and ileal pouch is performed. Although the hand-sewn IPAA procedure leaves a limited amount of rectal mucosa but requires a skillful operator, complications following IPAA have been reported to decrease as the experiences of the surgical team accumulates [146]. However, the development of adenoma in the ileal pouch is problematic following IPAA. The risk adenoma development in the ileal pouch include male sex, age < 18 years, and concomitant gastric adenomas, since the lesion may be cancerous in the long term [147]. Therefore, long-term surveillance of the ileal pouch with endoscopy is necessary even after total proctocolectomy.

IRA is the alternative recommended procedure for (1) patients with AFAP, (2) patients with a sparse type with < 20 rectal adenomas and maximum diameter < 10 mm, (3) young female patients who wish to be pregnant, and (4) young patients before school/employment [92, 148, 149] (Side Memo 8: Surgery and fertility, pregnancy, and delivery). A comparison between IPAA and IRA is indicated in Table 14. In patients with mesenteric desmoid tumor complications, the IRA procedure tends to be selected due to the difficulties in performing IPAA; however, there are opinions suggesting that IPAA may be conducted when the ileal pouch reaches the pelvic floor [12].

**Table 14:** Comparison between IPAA and IRA

	IPAA	IRA
Defecation frequency [150]	Frequent	Minimal
Nocturnal defecation [150]	Frequent	Minimal
Pad usage [150]	Frequent	Minimal
Fecal urgency [150]	Minimal	Frequent
Postoperative sexual function [150]	Similar	Similar
Dietary restriction [150]	Similar	Similar
Long-term complications [150]	Similar	Similar
Re-operation [150]	Frequent	Minimal
Residual rectal carcinoma [150]	Minimal	Frequent
Postoperative desmoid tumor [58, 67]	Frequent	Minimal

Total proctocolectomy + permanent ileostomy, which used to be administered before anus-preserving techniques became widely used, is rarely performed at present as prophylactic surgical treatment.

According to the multicenter survey in JSCCR, the percentage of patients who received prophylactic total proctocolectomy in their teenage years has decreased, whereas the percentage of those in their 20–40 s has increased [151]. The percentages of colorectal cancer development in patients with FAP by adenoma density (severe FAP, sparse FAP, and AFAP) and their age were as follows: 27, 31, and 46 years, 10%; 41, 48, and 59 years, 50%. These results indicate that timing for prophylactic total proctocolectomy should be considered based on the endoscopic findings of colon tumors in patients with classical FAP (severe FAP and sparse FAP) after the age of 20 years, (Chapter II 4-2: Treatment of colorectal adenomas [surgical timing of prophylactic proctocolectomy]). Furthermore, the development of colorectal cancer in patients with AFAP happens at a relatively advanced age; therefore, the timing for operation should be determined individually, based on the endoscopic findings.

Currently, the intervention trail for colorectal cancer prevention through endoscopic polypectomy in patients with FAP involves enrolling patients who refuse to undergo surgery in several institutions in Japan (Side Memo 11).

Side Memo 11 ■ Endoscopic removal of colorectal polyps for colorectal cancer prevention in patients with FAP.

Advances in colonoscopic treatment techniques have enabled the safe removal of a large number of colonic polyps, so studies have attempted to conduct follow-up observations while endoscopically removing a large number of colonic polyps in patients who refused to undergo surgery [152]. This report indicated no perforations or serious bleeding during endoscopic treatment, and there was no occurrence of advanced cancer during follow-up observations. This study was conducted at a single institution, and a prospective multicenter study is ongoing in Japan. It is an exploratory clinical phase I and II study for safety and efficacy, and the endpoint is the presence or absence of colorectal surgery during the intervention period.

## **CQ-6**: Is temporary ileostomy recommended in patients with FAP undergoing IPAA?

Temporary ileostomy in patients with FAP undergoing IPAA is weakly recommended (Recommendation 2/Evidence level C).

I study for safety and efficacy, and the endpoint is the presence or absence of colorectal surgery during the intervention period.

## Comments

A meta-analysis comparing complications after IPAA showed that the temporary ileostomy group had a lower incidence of anastomotic leakage than the non-ileostomy group, but a higher incidence of anastomotic stricture and bowel obstruction [153].

It has been reported that temporary ileostomy can be avoided under the following circumstances: (1) stapled anastomosis, (2) absence of anastomotic tension, (3) complete anastomosis, (4) sufficient hemostasis, (5) absence of anastomotic air leak, and (6) no evidence of malnutrition, infection, anemia, or regular steroid use [154]. From these points of view, it is considered that temporary ileostomy may be useful in the prevention of anastomotic leakage and pelvic abscess or suppression of the degree of these adverse events as much as possible after IPAA. However, it should be noted the abovementioned studies included both patients with ulcerative colitis and those with FAP, the latter accounting only for a small proportion of the subjects.

Studies of IPAA conducted on patients with FAP only have reported that temporary ileostomy was performed in most patients, while some patients underwent stapled IPAA without temporary ileostomy [155, 156]. In a study on the usefulness of temporary ileostomy in patients with FAP aged < 20 years, patients in whom temporary ileostomy was not performed showed favorable long-term defecation control but had significantly higher incidence of anastomotic leakage within 30 days of surgery (17.2vs. 0%, *P* = 0.002) and higher reoperation rate (20.7 vs. 4.6%, *P* = 0.02) [157]. Furthermore, of the 97 patients who underwent temporary ileostomy, 21 developed desmoid tumors, while there were 18 patients who did not undergo temporary ileostomy, of which none developed desmoid tumors (22 vs. 0%) [158]. However, most subjects included in this study underwent stapled IPAA, and further studies on temporary ileostomy in patients undergoing hand-sewn IPAA are required.

The JSCCR multicenter study showed that temporary ileostomy was performed in 55% of patients who underwent IPAA and more commonly performed with laparoscopic surgery (77.8 vs. 38.1%, *P* < 0.0001) [94]. Hand-sewn IPAA was also conducted more often in some high-volume centers, with fewer cases of temporary ileostomy [159].

A systematic review of the closure of temporary ileostomy [160] revealed that closure was safe but that 16.5% of subjects had postoperative complications, including bowel obstruction in 7.6% (reoperation in 2.9% of all cases), anastomotic leakage in 2.0%, wound infection in 4.0%, and late complications, such as incisional hernia in 1.9% and bowel obstruction in 9.4%.

Considering those mentioned above, temporary ileostomy can be avoided in selected patients with FAP undergoing IPAA, but it is difficult to clarify its indications. Therefore, it is practical to determine the need for temporary ileostomy on a case-by-case basis, considering its advantages and disadvantages.

## **CQ-7**: Is laparoscopic surgery recommended in patients with FAP?

Laparoscopic surgery for proctocolectomy in patients with FAP is weakly recommended (Recommendation 2/Evidence level C).

## Comments

Recently, laparoscopic surgery has been used in an increasing proportion of patients undergoing IPAA or IRA (IPAA, 23–53%; IRA, 58–62%) [119, 161–163]. According to previously published retrospective studies, laparoscopic surgery requires a longer time, but there are no differences between laparoscopic and open surgeries in the incidence rate of postoperative complications, mortality rate, reoperation rate, or readmission rate [162, 164–167]; furthermore, the laparoscopic approach yields better cosmesis with less intraoperative bleeding and shorter length of hospital stay [164, 165, 167]. In addition, laparoscopic surgery was also reported to be associated with a lower incidence of postoperative bowel obstruction, due to lower risk of occurrence of intra-abdominal adhesions and lower incidence of postoperative infertility in women 119). Moreover, no differences in postoperative sexual or anal function were observed between laparoscopic surgery and open surgery [168, 169]. According to the JSCCR multicenter study, which collected cases from 2000 to 2012, laparoscopic surgery has been used in > 70% of cases [93], and among the subjects of this study, the laparoscopic approach had been used in 74 of 171 (43%) patients undergoing IPAA and 52 of 85 (61%) patients undergoing IRA [94].

Large randomized controlled trials that compared open and laparoscopic surgeries for colorectal cancer in the general population serve as references for laparoscopic surgery in patients with FAP with colorectal cancer complications [170, 171]. There are no prospective studies on the safety of patients with FAP with advanced colorectal cancer from an oncological perspective. In practice, laparoscopic total proctocolectomy or colectomy with lymph node dissection is thought to be widely performed in clinical practice, similar to cases of sporadic colon and rectal cancer, but the details of the surgical outcomes are unclear.

Despite the longer operative time of laparoscopy, its safety and short-term results are assured, and indications of laparoscopic surgery for the present disease should be determined according to the proficiency of the institution (understanding of the laparoscopic surgery and present disease) and based on sufficient informed consent. Laparoscopic surgery should be an option for cases with colorectal cancer complications, but its indication should be decided upon consideration of the stage and site of the cancer and using various guidelines such as the “Practice Guidelines for Endoscopic Surgeries” by the Japan Society for Endoscopic Surgery [172].

## **CQ-8**: Is chemoprevention for colorectal adenomas recommended in patients with FAP?

It is strongly recommended that chemoprevention not be performed for colorectal adenomas in patients with FAP because evidence on agents in terms of efficacy and safety is still lacking (Recommendation 1/Evidence level A).

## Comments

Sulindac, an NSAID, has been studied to determine the effects of chemoprevention on patients with FAP. In randomized controlled trials, sulindac has not been shown to reduce the incidence of colorectal adenomas [173] but has been shown to reduce the incidence and size of colorectal adenomas in patients with FAP [174–176]. However, the number and size of colorectal adenomas increased after sulindac treatment was discontinued [174]. Long-term treatment with sulindac in patients with FAP after IRA inhibited the increased number or size of colorectal adenomas and development of atypical adenomas, but half of the patients exhibited rectal mucosal damage [177]. To date, there is no evidence that sulindac reduces the risk of developing colorectal cancer in patients with FAP. Moreover, its efficacy as chemopreventive agent is limited because its long-term treatment induces mucosal damage and discontinuation of the drug given the increases in tumorigenesis.

Randomized controlled trials of chemoprevention with celecoxib, which is a selective cyclooxygenase-2 (COX-2) inhibitor [178], showed a decreased number or size of colorectal adenomas, but it increases risk of cardiovascular events was a problem [179]. A randomized controlled trial reported that eicosapentaenoic acid, which is a fish oil, reduced the number or size of colorectal adenomas in patients with FAP [180], but no effects on reducing the onset of colorectal adenomas in the general population were observed in the general population [181].

The CAPP1 trial was a randomized controlled trial that analyzed the effects of chemoprevention with high-dose aspirin (600 mg/day) and indigestible starch on younger patients with FAP (10–21 years), but neither reduced the number of adenomas from the sigmoid colon to the rectum [182]. A small, double-blind, randomized trial (J-FAPP Study II) using low-dose aspirin (100 mg/day for 6–10 months) was conducted in Japan, but no reductions in the primary endpoint of adenoma size were detected [183]. No studies indicated that aspirin controlled the increase in the number or size of colorectal adenomas in patients with FAP.

## **CQ-9**: Is surveillance for extra-gastrointestinal lesions recommended in patients with FAP?

At this time, surveillance for extra-gastrointestinal lesions in patients with FAP is weakly recommended (Recommendation 2/Evidence level C).

## Comments

Thyroid cancer, brain tumor, hepatoblastoma, etc., are known to develop in patients with FAP.

Thyroid cancer is reported to develop in 2.6–11.8% of patients with FAP [60, 184, 185]. Most patients were female (male:female ratio = 1:44–80), and the average age is 25–33 years. A study indicated that these tumors developed in 11.1 and 0% of female and male patients with FAP. Most cases are papillary thyroid cancers, with the characteristic histology of the cribriform–morular variant in > 50% of cases [186, 187]. If a cribriform papillary carcinoma is found, FAP should be suspected, and colonoscopy should be performed. The incidence rates of multiple and bilateral thyroid cancer are both high at 28.6–69% and 42–67%, respectively. However, due to its favorable prognoses, total thyroidectomy is not always recommended [188]. One study recommends ultrasonography in addition to palpation as a screening examination for thyroid cancer in patients with FAP.

Some patients with FAP develop brain tumors (Turcot syndrome, type 2). Medulloblastoma is the most common brain tumor (approximately 60%), and others include astrocytomas, ependymomas, pineoblastomas, gangliogliomas, and intracranial pharyngiomas [189]. The relative risk of brain tumors in patients with FAP compared to the general population is 7 (92 for medulloblastoma), and the average age is young at 18.5 years.

Adrenal tumors develop in 7.4–13% of patients with FAP [190–192]. Recently, Shiroky et al. reported that, of 311 patients with FAP, 48 (16%) had adrenal tumors, with a mean age at diagnosis of 45 years and bilaterality of 23%. The majority were discovered incidentally by CT: 80% were adenomas, > 97% were benign (other myelolipomas, hyperplasia, etc.), and only one was cancerous (approximately 2%).

## **CQ-10**: Is capsule endoscopy recommended in patients with FAP?

Capsule endoscopy in patients with FAP is weakly recommended in suspected case of development of malignant tumor within a range where it cannot be observed by upper gastrointestinal endoscopy (Recommendation 2/Evidence level D).

## Comments

Studies have indicated that capsule endoscopy for patients with FAP can be safely completed even after colorectal surgery or in younger patients [193–196]. However, it is essential that capsule endoscopy be conducted after proctocolectomy with attention given to confirming the presence of prior passage failure and capsule retrieval [197]. Two prospective observational studies [195, 196] on the duodenum (including the ampullary region) showed that upper gastrointestinal endoscopy was superior to capsule endoscopy (see Surveillance of colorectal adenomas), so observations should be conducted with upper gastrointestinal endoscopy to the extent possible.

The detection rate of jejunal and ileal polyps with capsule endoscopy in patients with FAP was 30.4–60% [193–196]. Meta-analysis results have shown that the development of jejunal and ileal polyps is positively associated with the development of duodenal polyps [195]. A study on the location of jejunal and ileal polyps using capsule endoscopy showed that, of 29 patients with FAP, 21 had small intestinal polyps, 76% of which were in the proximal jejunum versus 3% in the distal jejunum and ileum [198]. However, in Japan, there are few studies on jejunal and ileal cancers in patients with FAP since the incidence of carcinogenesis from small intestinal polyps is unknown. Furthermore, given that the efficacy of searching the small intestine using capsule endoscopy has not been shown, we suggest performing capsule endoscopy for patients with FAP when the development of malignancy is suspected within a range where it cannot be observed by upper gastrointestinal endoscopy.

## Chapter III. Lynch syndrome

## Overview

- Lynch syndrome is a hereditary autosomal dominant disease, mainly caused by pathogenic germline variants in one of the mismatch repair genes (Side Memo 12: Mismatch repair function).

These patients and their families are at an elevated risk of developing various malignancies, including colorectal and endometrial cancers.

## Comments

### [Clinical features]

- Colorectal cancers in patients with Lynch syndrome are characterized by early age at onset and development of multiple tumors (synchronous/metachronous) and preferentially develop in the right colon.
- The frequency of poorly differentiated adenocarcinoma is higher in Lynch syndrome-associated colorectal cancers than in sporadic colorectal cancers. The histological features of colorectal cancer associated with Lynch syndrome include the presence of tumor-infiltrating lymphocytes, medullary growth pattern, mucinous/signet-ring cell differentiation, and Crohn’s-like lymphocytic reaction [6, 199–201] (Chapter III 2-STEP 1: Characteristic pathohistological findings in MSI-H colorectal cancer).
- A variety of other Lynch syndrome-associated extra colorectal cancers such as endometrial cancer, ovarian cancer (CQ12), gastric cancer, small-bowel cancer, bile duct cancer, pancreatic cancer, renal pelvic/ureteral cancer, brain tumor, and skin tumor (CQ11) can also develop. Recently, breast cancer, bladder cancer [202], and prostate cancer [203] have also been reported to develop in association with Lynch syndrome.
- The risk of Lynch syndrome-associated tumors varies depending on the causative gene, type of variant, environmental factors, etc. Pathogenic variant carrier does not always develop Lynch syndrome-associated tumors [14, 199, 204–209] (Table 15).

**Table 15:** Cumulative lifetime risk of Lynch syndrome-associated tumors (up to the age of 70 years)

Type	Cumulative risk
Colorectal cancer	54–74% (men)
	30–52% (women)
Endometrial cancer	28–60%
Gastric cancer	5.8–13%
Ovarian cancer	6.1–13.5%
Small-bowel cancer	2.5–4.3%
Bile duct cancer	1.4–2.0%
Pancreatic cancer	0.4–3.7%
Renal pelvic/ureteral cancer	3.2–8.4%
Brain tumor	2.1–3.7%
Sebaceous gland tumor	Unknown

### [Major causative genes]

- Pathogenic germline variant in any of the following genes:

*MLH1* on chromosome 3

*MSH2*, *MSH6*, and *EPCAM* (only a 3′ deletion in the case of *EPCAM*) on chromosome 2.

*PMS2* genes on chromosome 7

### [Mode of inheritance]

- Autosomal dominant inheritance

### [Mechanisms of malignant transformation]

- In patients with Lynch syndrome, a pathogenic germline variant is present in one allele of one of the mismatch repair genes, and an acquired genetic change (or methylation of the promoter region) in the other (wild type) allele impairs mismatch repair function. As a result, deviations in the number of tandem repeats often occur in the tumors. The genes involved in tumor suppression (e.g., *TGFBR2*), cell proliferation, DNA repair (e.g., *MSH3* and *MSH6*), apoptosis (e.g., *BAX*), etc., contain repetitive sequences in the coding regions, and alterations tend to develop in these regions.
- The adenoma–carcinoma sequence underlies the development of colorectal cancer in patients with Lynch syndrome, as in cases of sporadic colorectal cancer. There are many unknown aspects on the details of this sequence (Chapter I Fig. 3: Representative tumorigenesis mechanism of FAP and Lynch syndrome).
- The *EPCAM* (*TACSTD1*) is located upstream of the *MSH2*, adjacent to it, and deletion of its 3′ region (sequences necessary for transcription termination) can cause Lynch syndrome. The deletion causes fusion transcript of the *EPCAM* and *MSH2*, thereby inducing aberrant methylation of the *MSH2* promoter region followed by loss of expression of the MSH2 protein. The risk of colorectal cancer development in individuals with this *EPCAM* deletion is comparable to that in patients with pathogenic variant in *MSH2*, although the risk of endometrial cancer development is lower in these individuals [210]. *EPCAM* deletions have been reported to account for 1–3% of Lynch syndrome cases [211].

### [Incidence]

- Recent studies have shown that it accounts for 0.7–3.7% [8–10] of all colorectal cancers.
- The incidence in the Japanese general population is unknown.

Side Memo 12

- ■ Changes in the nomenclature of Lynch syndrome

In 1966, Lynch et al. [212] reported families in which colorectal and endometrial cancers were more frequently encountered than in the general population. In 1984, Boland et al. [213] classified the conditions into two categories; Lynch syndrome I, characterized by an increased risk of development of only colorectal cancer, and Lynch syndrome II, characterized by increased risk of development of not only colorectal cancer but also cancer of other organs in the family members. These two conditions have come to be collectively called Lynch syndrome or HNPCC. In 1990, however they were unified as HNPCC, and in a workshop of the International Collaborative Group on HNPCC (ICG-HNPCC) held in Amsterdam, standardized Amsterdam criteria I [214] were proposed to collect HNPCC pedigrees. Causative genes have been reported one after another since 1993. As a result, it had been found that there are many Lynch syndrome families but do not fulfill the Amsterdam criteria I meanwhile many others that fulfill the Amsterdam criteria I but in whom no causative genes can be identified. Therefore, in 1998, the revised Amsterdam criteria (Amsterdam criteria II) (Table 16), taking into consideration the development of malignant tumors other than colorectal cancer, such as endometrial cancer, were proposed for collaborative research on HNPCC [215]. Thereafter, the appropriateness of the term HNPCC was repeatedly discussed, and it was thought that the term is inappropriate considering the characteristics of the disease, i.e., the development of various malignant tumors other than colorectal cancer. Currently, the term Lynch syndrome, named after Dr. Lynch, is commonly used.

- ■ Mismatch repair function

**Table 16:** Amsterdam criteria II (1999) [215]

At least three relatives have Lynch syndrome (HNPCC)-associated tumors (colorectal cancer, endometrial cancer, renal pelvic/ureteral cancer, small-bowel cancer), and all of the following criteria should be fulfilled:
1. One of the affected individuals is a first-degree relative to the other two relatives
2. Afflictions are present across at least two generations
3. At least one cancer has been diagnosed before the age of 50 years
4. The tumor has been pathologically confirmed to be cancer
5. FAP has been excluded

Cells have the intrinsic function of detecting and repairing DNA mismatches that occur during DNA replication. Mismatch repair dysfunction increases the frequency of mispairs and insertions/deletions of simple repetitive sequences by 10–1,000-fold, which results in MSI (Side Memo 14: Method for MSI testing and evaluation of the results).

- ■ Germline epimutation

Recently, it was found that epimutations are involved in tumorigenesis in some cases of Lynch syndrome. Epimutations refer to modifications of molecules involved in gene expression, such as aberrant DNA methylation, that can cause changes in gene expression without alterations in the DNA sequence. Although rare, aberrant germline methylation (hypermethylation) of the promoter region of the *MLH1* gene has been reported as a cause of Lynch syndrome [216].

## Diagnosis

## Flow of diagnosis

**Fig. 21:**
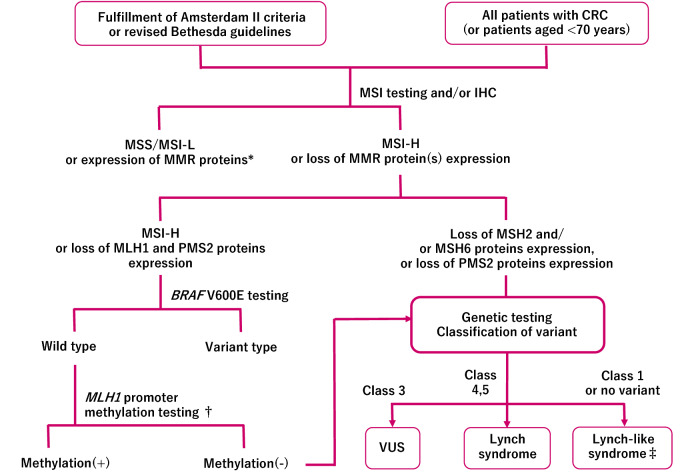
Diagnostic process for Lynch syndrome. MSI microsatellite instability, IHC immunohistochemistry, MSI-H high-frequency MSI, *MSI-L* low-frequency MSI, MSS microsatellite stability, MMR mismatch repair, VUS variant of uncertain significance. *Does not continue to genetic testing, ^†^Only *MLH1* methylation testing may be performed without *BRAF* V600E testing. ^‡^Loss of MMR protein(s) expression probably due to somatic two hits

**Fig. 22:**
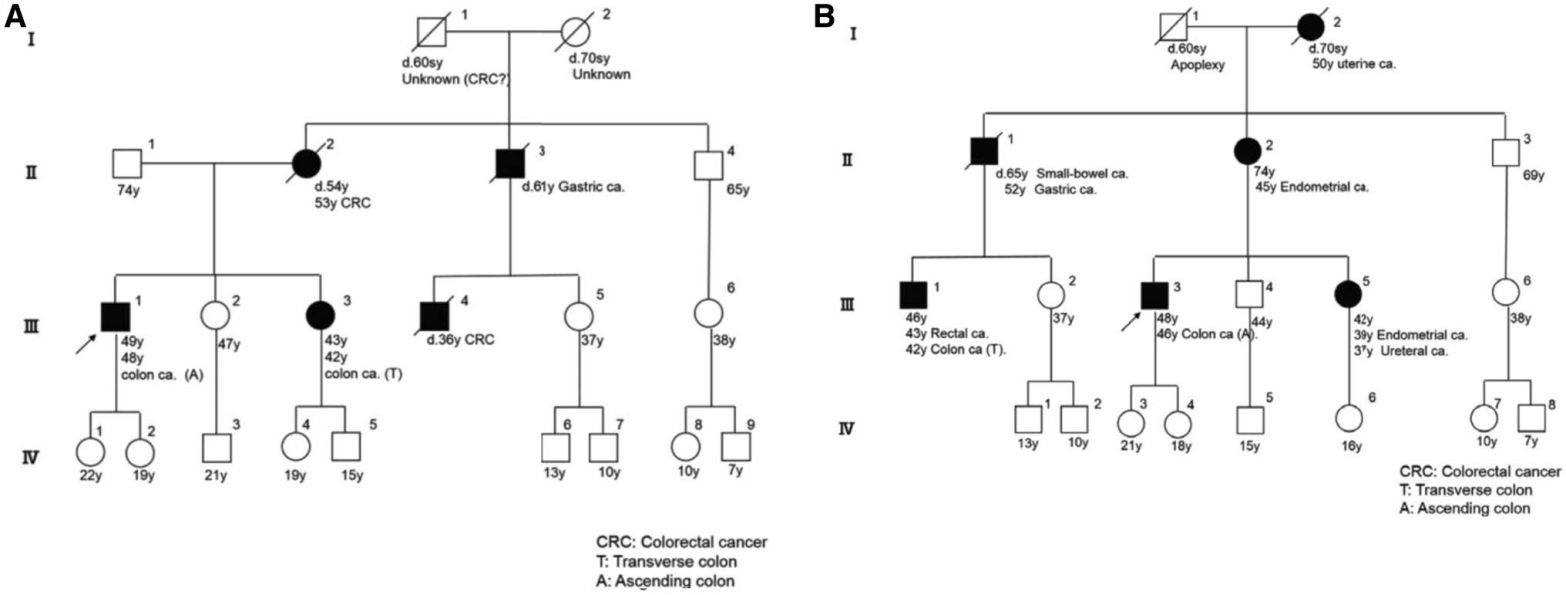
Family history fulfilling Amsterdam criteria II [215] (Appendix: Principles of writing and reading pedigrees). **a** Multiple family members with colorectal cancer. **b** Multiple family members with colorectal cancer and Lynch syndrome-associated extracolonic cancers

- ■ Diagnosis should be made according to the following STEP 1 to STEP 3 in patients with clinicopathological findings (including family history) suggestive of Lynch syndrome (Fig. 21).

- STEP 1: It should be checked whether the patient fulfills the Amsterdam criteria II [215] (Table 16, Fig. 22a, b) or revised Bethesda guidelines [217] (Table 17) (primary screening). For universal screening, all colorectal and endometrial cancers (or those age < 70 years) continue to STEP 2 (CQ14).
- STEP 2: MSI testing or IHC for the causative gene products in the tumor tissue should be performed to confirm high-frequency MSI (MSI-H) or the loss of mismatch repair proteins (secondary screening) (Side Memo 13: Precautions for MSI testing in screening tests for patients with Lynch syndrome, Side Memo 14: Evaluation of MSI test methods and results)
- Cases showing MSI-H or loss of MLH1/PMS2 expression do not need to continue to STEP 3 if the tumor has the *BRAF* V600E variant or *MLH1* promoter methylation.
- STEP 3: Genetic testing is conducted to identify a pathogenic germline variant in mismatch repair genes as a definitive diagnosis. (CQ15).

**Table 17:** Revised Bethesda guidelines (2004) [217]

Tumors from patients with colorectal cancer should be tested for MSI in the following conditions:
1. Colorectal cancer diagnosed in a patient aged < 50 years
2. Presence of synchronous, metachronous colorectal, or other Lynch syndrome (LS)-associated tumors^a^, regardless of the age
3. Colorectal cancer with MSI-H histology^b^ diagnosed in a patient aged < 60 years
4. Colorectal cancer diagnosed in a patient with one or more first-degree relatives with a LS-associated tumor, with one of the cancers being diagnosed at the age < 50 years
5. Colorectal cancer diagnosed in two or more first- or second-degree relatives with LS-associated tumors, regardless of the age

^a^Colorectal cancer, endometrial cancer, gastric cancer, ovarian cancer, pancreatic cancer, biliary tract cancer, small-bowel cancer, renal pelvic/ureteral cancer, brain tumors (usually glioblastoma in Turcot syndrome), sebaceous gland adenoma, and keratoacanthoma in Muir–Torre syndrome

^b^Tumor infiltrating lymphocytes, Crohn’s-like lymphocytic reaction, mucinous carcinoma/signet-ring differentiation, or medullary growth pattern

## Universal screening

Recently, universal screening, in which MSI testing or IHC for mismatch repair proteins is performed in all patients (or patients aged < 70 years) with colorectal or endometrial cancer, is recommended as a highly sensitive and cost-effective method for the diagnosis of patients with Lynch syndrome in Western countries.

## Comments

- Screening sensitivity with MSI testing, IHC for mismatch repair proteins, and the combination of both all showed high sensitivity in a recent pool analysis, with 0.93 (95% confidence interval [CI], 0.87–0.96), 0.91 (95% CI 0.85–0.95), and 0.97 (95% CI 0.90–0.99), respectively [218].
- The incidence of Lynch syndrome detected by universal tumor screening is reported to be 2.4–3.7% of all colorectal cancers [8, 9].
- Although mismatch repair deficient colorectal cancer tends to be more frequent among the patients with advanced age onset colorectal cancer, Lynch syndrome-associated colorectal cancer tends to be rarely included among them [10, 219, 220]. Therefore, screening patients under a certain age (e.g., age < 70 years) has been proposed in all patients with colorectal cancers, considering the efficiency and cost-effectiveness of screening.
- In Western countries, it is thought that MSI testing and IHC for screening Lynch syndrome do not require individual patient consent, but in Japan, it is recommended that a prior explanation be provided in screening tests for patients with Lynch syndrome [221].

### STEP 1. Criteria for primary screening

## Comments

- It has been reported that 27–41% [212, 219] of families with Lynch syndrome fulfilled the Amsterdam criteria II [215] and that 68–89% fulfilled the revised Bethesda guidelines [217]; thus, more patients with Lynch syndrome can be identified using the revised Bethesda guidelines [223].
- Approximately one-fourth of all patients with colorectal cancer fulfilled the revised Bethesda guidelines [222]. However, most of them are not patients with Lynch syndrome but patients with sporadic colorectal cancer [217].
- In the JSCCR project studies, 1.2% of patients with colorectal cancer fulfilled the Amsterdam criteria II [223].

## Characteristic histopathology of MSI-H colorectal cancer

**Fig. 23:**
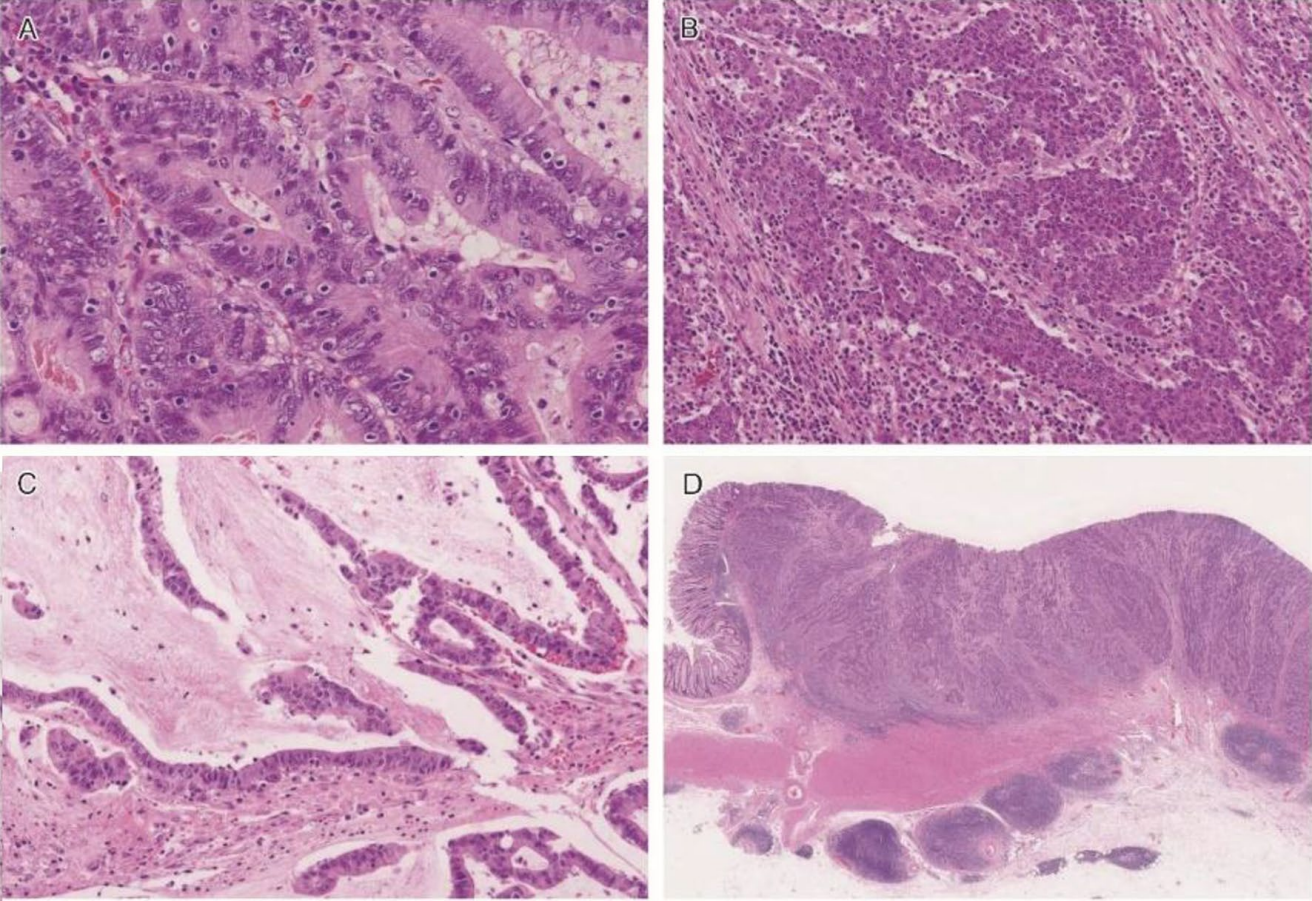
Histologic characteristics of MSI-H colorectal cancer. **A** Tumor infiltrating lymphocytes. Numerous intra-epithelial lymphocytes showing clear halos. **B** Medullary carcinoma. Tumor showing a solid growth pattern without glandular structure. **C** Mucinous carcinoma. Prominent extracellular mucin noted. **D** Crohn’s-like lymphocytic reaction. Characterized by peritumoral lymphocytic aggregates

MSI-H colorectal cancer more commonly has several histologic characteristics compared with non-MSI-H colorectal cancer, so these findings are useful in selecting patients with suspected Lynch syndrome. The revised Bethesda guidelines [217] consider the following four findings: (1) tumor infiltrating lymphocytes, (2) medullary growth pattern, (3) mucinous carcinoma/signet-ring differentiation, and (4) Crohn’s-like lymphocytic reaction (Fig. 23a–d). However, these histologic features are not necessarily unique to Lynch syndrome and are also common to sporadic MSI-H colorectal cancers [224].

## STEP 2. Tests used for secondary screening

## MSI testing

In the tumor cells with impaired mismatch repair function, the number of repetitive sequences of one to several nucleotides in the genome is different from that in normal cells. This phenomenon is called MSI.

It is reported that MSI-H is observed in > 90% of colorectal cancers in patients with Lynch syndrome [225]. Meanwhile, the overall percentage of MSI-H in colorectal cancers was 12–16% in Western studies [225–227] and 6–7% in Japanese studies [228, 229]. Therefore, MSI testing is useful as a screening test for Lynch syndrome. In cases with clinical findings suggestive of Lynch syndrome, if the results of MSI testing of the colorectal tumor (colorectal adenomas can also be examined although the rate of detection is lower in adenoma than cancer) show MSI-H, Lynch syndrome should be strongly suspected.

MSI testing was covered by the national health insurance program from 2006 for malignancies in patients with colorectal cancer suspected of having Lynch syndrome. Sufficient explanations about possibility of hereditary cancer and consent are required when MSI test is performed. (Side Memo 14: Evaluation of MSI test methods and results).

Side Memo 13

- ■ Important point for MSI testing in screening Lynch syndrome.

In MSI testing, colorectal cancer from *MSH6* associated Lynch syndrome may not demonstrate MSI-H. [230, 231]. Therefore, genetic testing of the mismatch repair genes should be considered even for low-frequency MSI (MSI-L) or microsatellite stability (MSS) if the Amsterdam criteria II [215] are fulfilled or Lynch syndrome is strongly suspected from personal history (e.g., juvenile onset, multiple cancers) [13].

Side Memo 14

- ■ Evaluation of MSI testing methods and results.

**Fig. 24:**
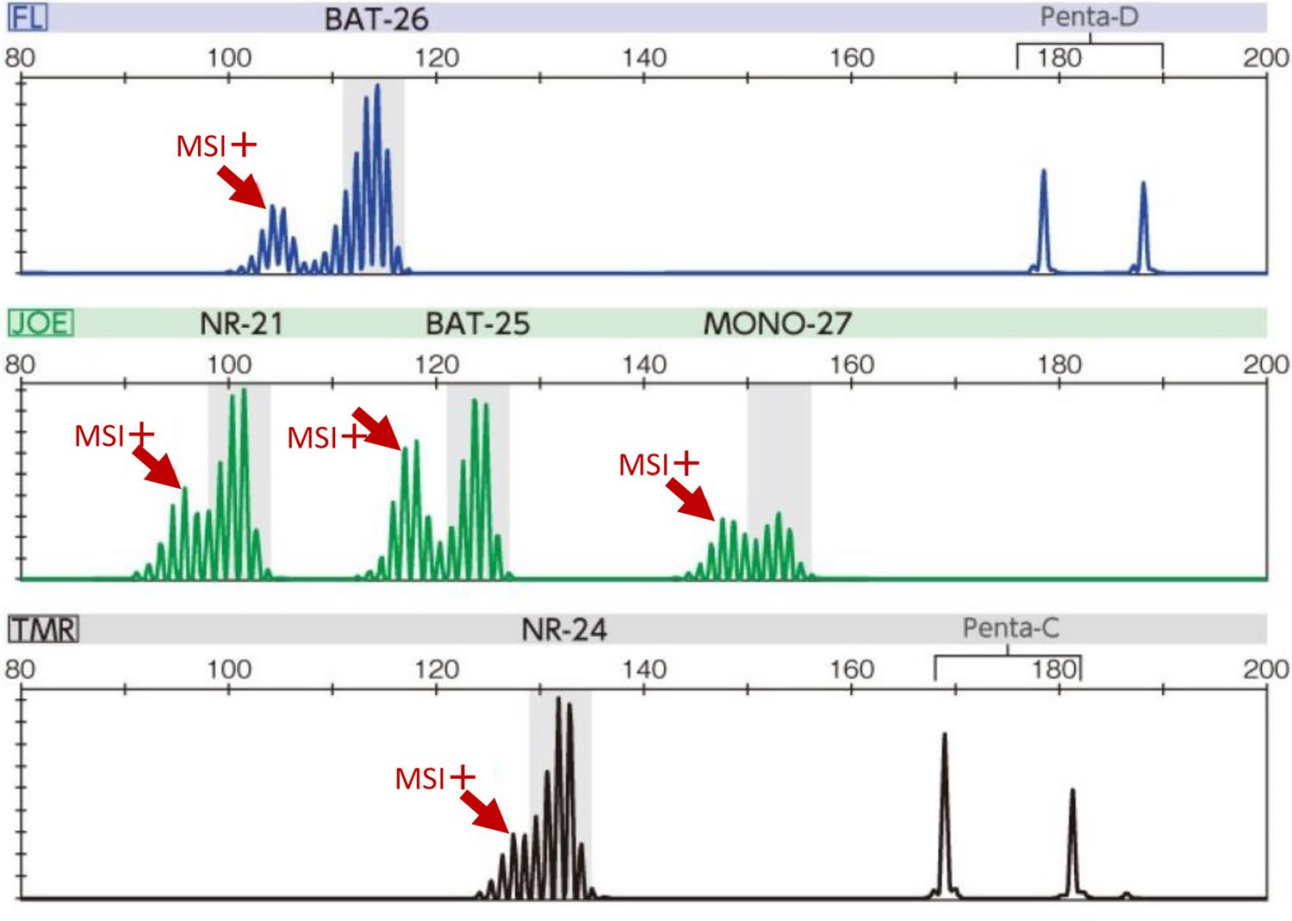
Example of MSI analysis using the Promega Panel. All five mononucleotide repeat markers (BAT-26, NR-21, BAT-25, MONO-27, NR-24) showed different microsatellite length, indicating to be MSI-H

Frozen samples or formalin-fixed paraffin-embedded specimens of tumor tissues are required for MSI testing. MSI of tumor tissues are generally determined using five types of mononucleotide repeat markers (Promega Panel: BAT-25, BAT-26, NR-21, NR-24, MONO-27) (Fig. 24). MSI is determined when the microsatellite length changes, with two or more markers indicating MSI corresponding to MSI-H, one or more markers indicating MSI corresponding to MSI-L, and neither marker indicating MSI corresponding to MSS (Fig. 24).

Conventionally used Bethesda markers (consisting of two mononucleotide repeat markers and three dinucleotide repeat markers) required normal tissue as a control for determining the length of microsatellites, but the mononucleotide repeat markers used in the Promega Panel showed little difference between individuals (i.e., quasi-monomorphic mononucleotide), so they can be determined only in the tumor tissue.

## IHC

Most Lynch syndrome-associated tumors have biallelic inactivation of one of the mismatch repair genes, namely, *MLH1*, *MSH2*, *MSH6*, and *PMS2*, and expression of the corresponding protein is lost in most cases. Because MSI-H is caused by mismatch repair deficiency, the results of MSI testing are highly consistent with those of IHC for mismatch repair proteins. The results of MSI testing and IHC are consistent in 90% of cases and that the false-negative rate of IHC for patients with Lynch syndrome is 5–10% [160, 185]. The advantages of IHC are that it can be implemented at many hospitals and it allows the causative gene to be deduced (Side Memo 15: Exceptional staining results).

Sufficient explanations and consent are required for implementing immunochemistry, similar to MSI testing.

Although the sensitivity and specificity of MSI testing and IHC are comparable, the cost and availability of each testing are different for each hospital. Therefore, the choice of which test to use depends on the situations of each hospital. If one testing is negative but Lynch syndrome is clinically suspected, the other testing may provide complementary screening.

- An internal positive control should be used to confirm the appropriateness of the staining during its evaluation.

## Internal positive controls

**Fig. 25:**
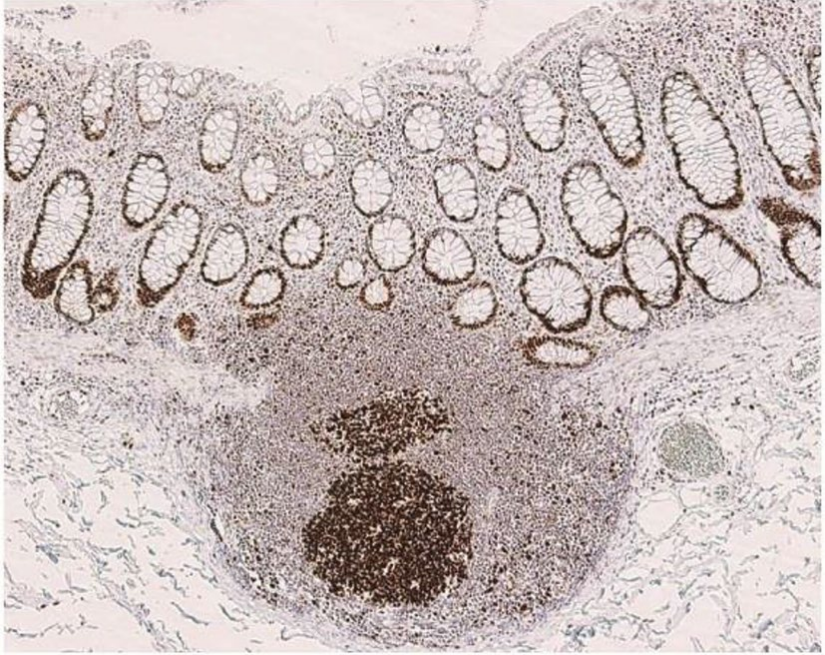
MSH2 expression in normal colon mucosa. Strong staining is noted in the germinal center of a lymphoid follicle and at the bottom of glands

Mismatch repair proteins are localized in the nuclei and more strongly expressed in proliferating cells. The base of the colonic crypts and germinal centers of lymph follicles are good positive controls in non-tumor tissues (Fig. 25). Because tumor cells generally have high proliferative activity, the evaluation of tumor tissue is usually feasible if staining of internal positive is confirmed.

## Staining patterns and evaluation

**Fig. 26:**
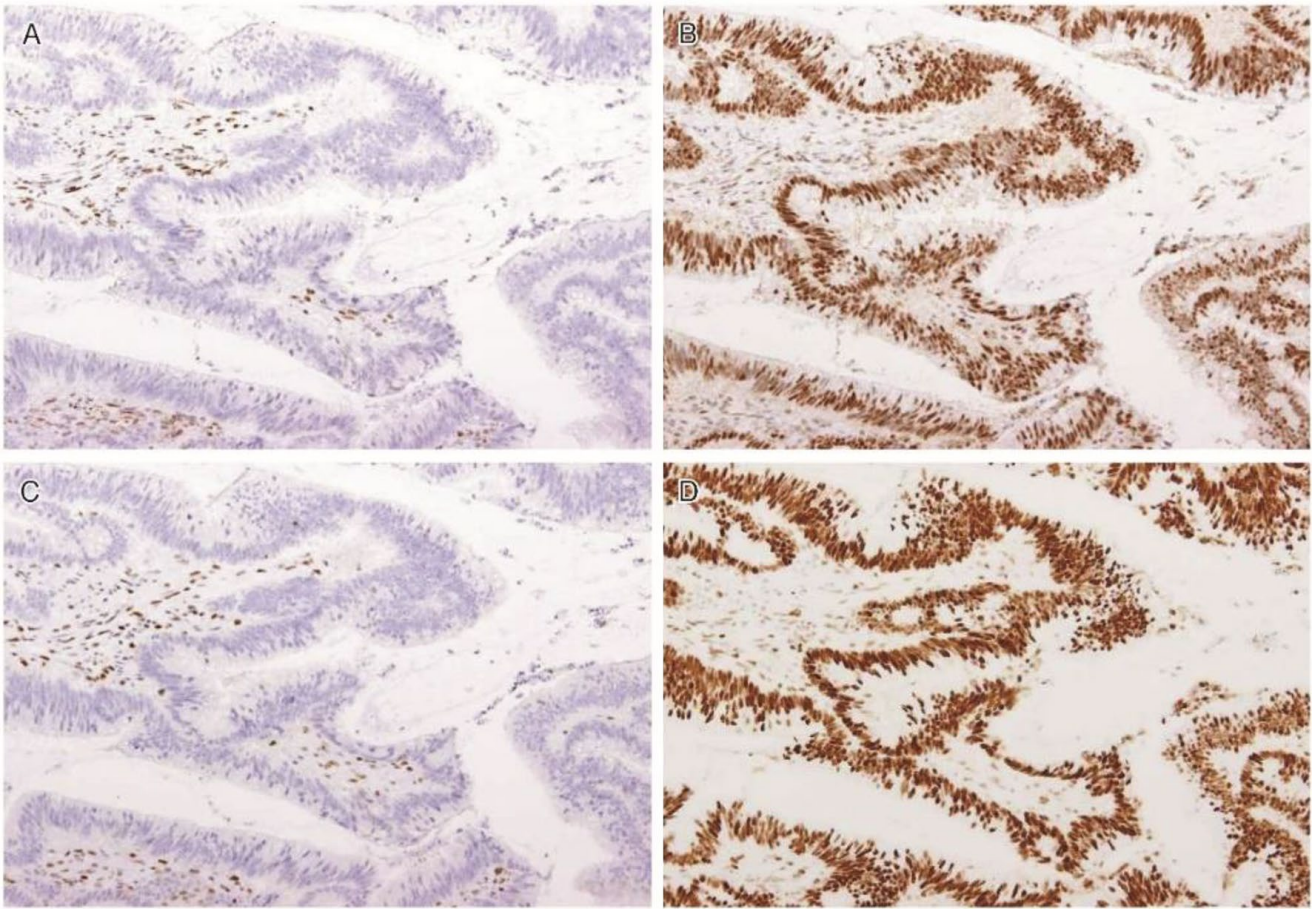
Immunohistochemistry for mismatch repair proteins in the colorectal cancer specimen resected from a patient with Lynch syndrome with a germline *MLH1 variant*. Loss of MLH1 (**a**) and PMS2 (**c**) and retention of MSH2 (**b**) and MSH6 (**d**). Stromal cells served as internal positive controls

In tumors without mismatch repair deficiency, expression of all four proteins is retained. In tumors with mismatch repair deficiency, expression of at least one protein is lost, reflecting the variants of mismatch repair genes. However, variants in mismatch repair genes do not always result in the isolated loss of the corresponding protein expression (Table 18, Fig. 26). Most cases exhibit one of the staining patterns shown in Table 18. If a staining pattern different from any of those shown in Table 18 is obtained, the validity of staining should be checked before considering the possibility of an exceptional case. In principle, invasive cancers show diffuse loss of expression.

**Table 18:** Relationship between immunohistochemical expression of the mismatch repair proteins and defective mismatch repair genes

	Immunohistochemical expressions			
	MLH1	MSH2	PMS2	MSH6
Suspected causative genes				
*MLH1*	−	+	−	+
*MSH2*	+	−	+	−
*PMS2*	+	+	−	+
*MSH6*	+	+	+	−

The expression of PMS2 and MLH1 is lost in tumors with *MLH1* variants, and the expression of MSH6 and MSH2 is lost in tumors with *MSH2* variants (Table 18). Therefore, the use of only two antibodies, anti-PMS2 and anti-MSH6 antibodies, allows the screening of Lynch syndrome to be performed with a sensitivity equivalent to that using four antibodies [232]. If the expression of PMS2 or MSH6 is lost, staining for MLH1 or MSH2, respectively, should be added to deduce the causative gene.

Side Memo 15 **■ Exceptional staining results**

- Expression of abnormal proteins due to missense variants

In some cases of missense variants, nonfunctional proteins are expressed. This is known to be relatively common in Lynch syndrome cases with *MLH1* variants, and only PMS2 expression is lost in most of these cases. However, there are rare cases in which no abnormalities are detected by IHC. Even if no abnormalities are detected by IHC, if the patient is clinically strongly suspected as having Lynch syndrome, the addition of MSI testing may allow the detection of microsatellite instability.

- Secondary variants in mismatch repair genes due to MSI

Some mismatch repair genes have repeat sequences in their coding regions, and secondary variants may occur in these genes. In some patients with *MLH1* variants (loss of MLH1/PMS2), MSH6 expression is lost diffusely or focally [233].

- Loss of MSH6 expression due to preoperative chemoradiotherapy

It has been reported that, in patients with colorectal cancer receiving preoperative chemoradiotherapy, the tumor MSH6 expression may be lost even in the absence of *MSH6* variants [233].

## *BRAF* V600E testing and *MLH1* promoter methylation testing:

- The majority of sporadic colorectal cancers exhibiting mismatch repair deficiency are caused by loss of MLH1 expression due to *MLH1* promoter methylation. Tests for the *BRAF* V600E variants and/or *MLH1* promoter methylation should be considered in cases with MSI-H and those with loss of MLH1 and PMS2 expression [234].
- Lynch syndrome can be principally ruled out in patients who are positive for the *BRAF* V600E variants and/or *MLH1* promoter methylation, so patients who do not need to continue to STEP 3 can be selected. *BRAF* V600E variant testing has been covered by the national health insurance program since 2018.
- Many international guidelines recommend methylation testing since *MLH1* promoter methylation testing can exclude sporadic cases at a higher sensitivity than *BRAF* V600E variant testing [13, 126, 235–238].
- As an exception, the *BRAF* V600E variants may be detected in colorectal cancers in patients with Lynch-syndrome due to *PMS2* variants [239].

## STEP 3.Tests for definitive diagnosis

## Genetic testing for mismatch repair genes

The patients’ blood is used to directly determine the presence or absence of pathogenic germline variants in the mismatch repair genes and 3′ deletions of *EPCAM*. If a pathogenic variant is identified, the patient is diagnosed with Lynch syndrome. Genetic counseling must be provided before and after the testing (Chapter I 2–3: Genetic counseling [p. 21]).

## Comments

- Even if genetic testing for variants of the mismatch repair genes is considered unnecessary in the screening process or genetic testing does not reveal any pathogenic variants in the causative genes, the patient may still have Lynch syndrome.
- For families with clinical features strongly suggestive of Lynch syndrome, genetic testing for variants in the mismatch repair genes is sometimes performed directly without screening by MSI testing or IHC.
- It is desirable to perform genetic testing for variants in the mismatch repair genes in individuals whose family members show clinical features suggestive of Lynch syndrome (multiple cancers, including colorectal cancer, endometrial cancer, early-onset cancer, etc.).

Side Memo 16

- ■ Muir–Torre syndrome

Muir–Torre syndrome is a condition characterized by synchronous skin tumors (sebaceous adenoma, sebaceous epithelioma, or sebaceous carcinoma) and/or keratoacanthoma in association with various tumors associated with Lynch syndrome, including colorectal cancer. Germline pathogenic variants are identified mostly with *MSH2* [240].

- ■ Turcot syndrome (type 1)

Turcot syndrome type 1 is a condition in which Lynch-syndrome-associated colorectal cancer is accompanied by brain tumors, mostly glioblastoma. Germline pathogenic variants in *MLH1* or *PMS2* as well as methylation of the promoter region of *MLH1* have been identified [241]. Caution should be exercised, since brain tumors are reported to be a major cause of death in patients with Lynch syndrome [242] (CQ9: Turcot syndrome type 2).

- ■ Constitutional mismatch repair deficiency (CMMRD)

CMMRD [36] is caused by pathogenic germline variants in both alleles of mismatch repair genes, and is inherited in an autosomal recessive manner. The types of cancers and age of onset are different from patients with Lynch syndrome. Of 146 cases from 91 families with CMMRD, 85 (58%) had pathogenic germline variants of *PMS2* in both alleles [37]. Cancers found with 146 patients were 81 (55%) central nervous system tumors (median age, 6 years), 59 (40%) colorectal cancer (median age, 16 years), and 48 (33%) hematologic tumors (median age, 6 years). Furthermore, a study reported a characteristic finding that more than 80% had café-au-lait spots similar to neurofibromatosis type 1 (NF1) [37].

## Diseases and conditions that should be differentiated from Lynch syndrome

## Sporadic colorectal cancer with MSI-H

Sporadic colorectal cancer with MSI-H is commonly characterized by occurrence in elderly women, occurrence of poorly differentiated adenocarcinoma, right-sided preponderance, etc. The primary cause of MSI-H is considered as an acquired aberrant methylation of the promoter region of the *MLH1* gene [243]. In these tumors, IHC shows loss of expression of the MLH1 protein. In addition, *BRAF* V600E variant is found in the tumor tissue in 35–43% of patients [244, 245]. In contrast, *BRAF* V600E variant is not detected in most colorectal cancers associated with Lynch syndrome, even if they show MSI-H [246]. Therefore, checking for the presence or absence of *BRAF* V600E variant is sometimes used to differentiate between these diseases.

## PPAP

The phenotype of PPAP [37–39] may be similar to that of FAP (AFAP) or Lynch syndrome and requires differentiation (Chapter II 2–3: Diseases needing differentiation). PPAP-associated colorectal cancers where POLE is the causative gene may exhibit MSI-H.

## Familial colorectal cancer type X

Patients who fulfill the Amsterdam criteria I [214] but in whom no pathogenic germline variants are detected in the mismatch repair genes or in whom colorectal cancer does not show MSI-H are unlikely to have Lynch syndrome, and the term “familial colorectal cancer type X” [247] have been proposed for the condition. Familial colorectal cancer type X is speculated to comprise multiple conditions. Studies from both Western countries and Japan [248] have shown that the risk of developing Lynch syndrome-associated tumors other than colorectal cancer is significantly lower in cases of familial colorectal cancer type X.

Note: Amsterdam criteria I: While colorectal cancer, endometrial cancer, renal pelvic/ureteral cancer, and small-bowel cancer are considered Lynch syndrome (HNPCC)-associated tumors in the Amsterdam criteria II, only colorectal cancer is considered Lynch syndrome (HNPCC)-associated tumor in the Amsterdam criteria I [214].

## Lynch-like syndrome

Colorectal cancers with mismatch repair deficiency (loss of MSI-H or mismatch repair protein expression) in which *MLH1* promoter methylation is not observed while not exhibiting mismatch repair genes or pathogenic *EPCAM* variants that cause Lynch syndrome are referred to as Lynch-like syndrome. Causes include biallelic somatic variants of mismatch repair genes, unidentifiable germline mismatch repair gene variants, and germline variants other than mismatch repair genes. There are still many unknown elements to this disease [249, 250].

## Treatment

## Treatment of colorectal cancer

- The following options exist for the extent of resection of the colorectum (types of surgical procedures) in patients with Lynch syndrome:

1. Extent of resection equivalent to that adopted for sporadic colorectal cancer.
2. Total colectomy.
3. Total proctocolectomy (TPC).

- No consensus has been reached on the usefulness of prophylactic proctocolectomy, and it is not generally recommended.

## Comments

- Because colorectal cancer tends to develop at multiple sites of the colorectum in patients with Lynch syndrome, including synchronous or metachronous development, the entire colorectum should be examined preoperatively.
- Some studies from Western countries have recommended extended operations, such as total colectomy for colonic cancer and TPC for rectal cancer, in patients with Lynch syndrome. (CQ16).
- Prophylactic colectomy for Lynch syndrome variant carriers is not generally recommended because its efficacy has not been assessed (CQ16).
- Colorectal cancers in most cases of Lynch syndrome show MSI-H. Although 5-fluorouracil (FU)-based anticancer drugs have been reported to be generally ineffective in colorectal cancers showing MSI-H, the usefulness of chemotherapy specifically in Lynch syndrome-associated colorectal cancer has not yet been clarified (CQ17, CQ18).

### Management of extracolonic tumors

1. Gastrointestinal tumors (gastric cancer, small-bowel cancer, bile duct cancer, pancreatic cancer, etc.).
2. Gynecologic tumors (endometrial cancer, ovarian cancer, etc.) (CQ12, CQ13).
3. Urological tumors (renal pelvic/ureteral cancer, etc.).
4. Other tumors (brain tumor, skin tumor, etc.).

There is no clear evidence on any special considerations required for the abovementioned tumors (1)–(4), except for the case of gynecologic cancers, in patients with Lynch syndrome. Presently, treatment such as that for the corresponding sporadic cancers (tumors) is used.

## Comments

- In patients with Lynch syndrome with colorectal cancer, it is desirable to conduct screening for other Lynch syndrome-associated tumors (particularly, gynecologic cancers, urological cancers, and gastrointestinal cancers) before elective colectomy.

## Postoperative surveillance

### Surveillance for multiple colorectal cancers and resection of adenomas

- Attention should be paid to the possible development of metachronous cancer in the remaining colorectum after surgery for colorectal cancer in patients with Lynch syndrome, and lifelong regular colonoscopy surveillance is required (CQ12).

## Comments

- Surveillance for recurrence of colorectal cancer after resection should be in accordance with the protocol used for cases of sporadic colorectal cancer.
- Colorectal adenomas, if detected, should be resected because they may develop into colorectal cancer.

### Surveillance for Lynch-syndrome-associated extracolonic tumors

- Specialist groups proposed surveillance methods shown in Table 19 for the main Lynch syndrome-associated tumors.

**Table 19:** Recommended surveillance protocols for common Lynch syndrome-associated tumors

Site	Test method	Age at the start of testing	Test interval	Comments	References
Colorectum	Colonoscopy	20–25 years	1–2 years		[13, 126, 235–238]
Uterus, ovary	Transvaginal US, endometrial biopsy	30–35 years	1 year		[13, 126, 235–238]
	(or cytology) (CA-125)				
Stomach, duodenum	HP infection	30–35 years		Sterilization if HP infection is present	[13, 126, 235–237]
	Upper gastrointestinal endoscopy	30–35 years	1–3 years	Consider in populations at high risk of gastric cancer or with a family history of gastric or duodenal cancer	[13, 126, 235–237]
Urinary tract	Urinalysis (or urine cytology)	30–35 years	1 year	Consider in cases where *MSH2* variants or a Family history of urothelial carcinoma are present	[13, 235–238]

## Comments

- Screening and treatment for *Helicobacter pylori* (*HP*)-associated gastritis should be considered. It was proposed that surveillance be conducted by upper gastrointestinal endoscopy every 1–3 years in areas where gastric cancer is common, such as East Asia, and in patients with Lynch syndrome with a family history of gastric cancer and their relatives [251].
- No consensus has been reached on the method or interval of regular surveillance for endometrial and ovarian cancers (CQ12).
- Lynch syndrome-associated urological tumors include renal pelvic/ureteral cancer. This type of cancer is common in patients with germline variants in the *MSH2* gene, but none of the surveillance methods, including regular urinalysis and urinary cytology, have been demonstrated to be useful in improving prognosis.

## Measures for patients with colorectal cancer without a diagnosis of Lynch syndrome

## When genetic testing has not been conducted

**Fig. 27:**
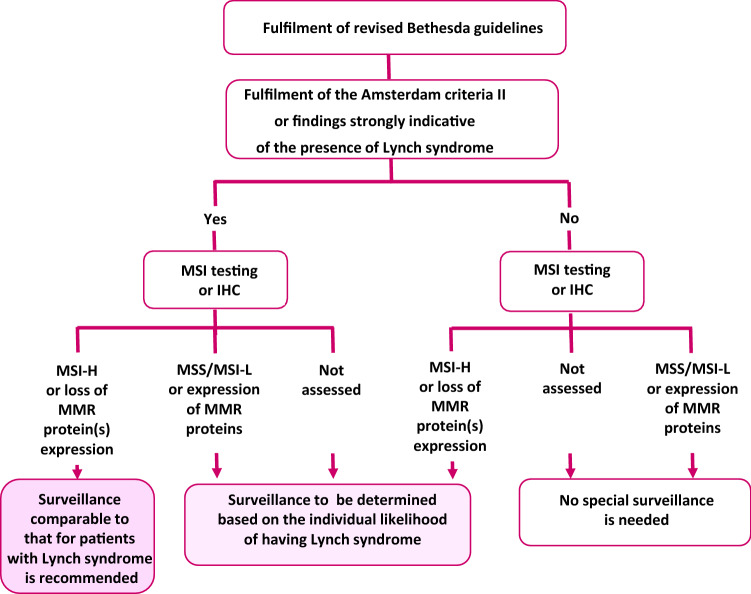
Management of individuals without a definitive diagnosis of Lynch syndrome

- In patients who are suspected to have Lynch syndrome but have not been diagnosed yet by genetic testing, the possibility of Lynch syndrome should be individually evaluated, and surveillance of Lynch syndrome-associated tumors should be conducted based on clinical information and results of MSI testing or IHC of the MMR protein (Fig. 27).

## When the genetic test result was VUS

- Patients with VUS who have undergone genetic testing for Lynch syndrome (Fig. 21) are offered surveillance according to their tumorigenesis status within the pedigree. In the absence of a dense family history, special surveillance is not required, and general screening is performed as that for sporadic colorectal cancer.

## When family history is strongly suggestive of Lynch syndrome but not confirmed by genetic testing

- Patients with colorectal cancer who have a dense family history suggestive of Lynch syndrome but do not have a pathogenic variant are considered to have Lynch syndrome for surveillance.

## Comments

- In cases where the patient “fulfills the Amsterdam criteria II” or “has a past or family history highly suggestive of Lynch syndrome,” in addition to the results of MSI testing showing MSI-H or loss of MMR proteins expression, the patient should be regarded as having Lynch syndrome, and surveillance should be conducted even if no genetic testing has been performed.
- In cases where the patient “fulfills the Amsterdam criteria II” or “has a past or family history highly suggestive of Lynch syndrome” but the results of MSI testing show MSS/MSI-L or expression of MMR proteins (there are no findings strongly suggestive of mismatch repair gene deficiency), Lynch syndrome cannot be certainly ruled out (Side Memo 13: Precautions for MSI testing in screening tests for patients with Lynch syndrome). In these cases, follow-up should be subsequently performed while paying attention to the personal and family history, with colonoscopy examination for colorectal cancer conducted at least every 3–5 years.
- In cases where the patient “fulfills the revised Bethesda guidelines, but not the Amsterdam criteria II, or does not have a personal or family history strongly suggestive of Lynch syndrome,” if the results of MSI testing show MSI-H or loss of MMR proteins expression, the patient may have Lynch syndrome (many patients are likely to have sporadic colorectal cancer). Follow-up should be performed while validating the past and family history.
- In cases where the patient has colorectal cancer with MSS/MSI-L or expression MMR proteins, in addition to the family and medical history showing unlikely to have Lynch syndrome, surveillance for patients with Lynch syndrome-associated tumors is not conducted. When patients have symptoms of colorectal cancer or Lynch syndrome-associated tumors are observed in the patients or their relatives, detailed examination and reevaluation for Lynch syndrome are recommended.

## Genetic counseling and management of families (relatives)

- It is desirable to provide genetic counseling to not only the patients but also their relatives.
- After providing an adequate explanation about the disease to first-degree relatives (parents, children, and siblings) and obtaining their consent, surveillance for Lynch syndrome-associated tumors should be conducted according to the assessed risk.

## Comments

- In principle, because Lynch syndrome-associated tumors generally develop in adulthood, genetic testing should be performed in adulthood.

## Management of the families (relatives) of patients who have been definitively diagnosed with Lynch syndrome

## Comments

- Relatives who are definite variant carriers or have not undergone genetic testing should be regarded as having Lynch syndrome and undergo surveillance for Lynch syndrome-associated tumors (Fig. 28).
- Relatives who have been confirmed to have no pathogenic variants should undergo general cancer screening (Fig. 28). Information on the necessity of surveillance and significance of genetic diagnosis should be provided to relatives who have reached the age of surveillance for Lynch syndrome-associated tumors. Everyone should decide, of his/her own free will, whether he/she wishes to undergo genetic testing or refuses to undergo genetic counseling.

## Management of patient families (relatives) who are suspected to have Lynch syndrome but for whom no definitive diagnosis has been made

**Fig. 28:**
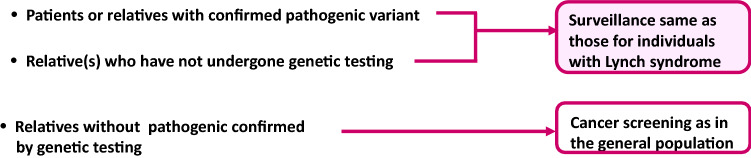
Management of families (relatives) of patients who have been definitively diagnosed with Lynch syndrome

## Comments

- In relatives of patients who have not undergone genetic testing or in whom genetic testing have failed to yield a diagnosis of Lynch syndrome, individual risk assessment should be conducted based on the age of onset, incidence, etc., of Lynch syndrome-associated tumors in family members, and surveillance for associated tumors should be conducted.
- In relatives of patients suspected as having Lynch syndrome, surveillance should be conducted according to the protocol shown in Table 19, or colonoscopy should be started at an age of 5–15 years younger than that of the earliest age at diagnosis of colorectal cancer in the family.

## Clinical Questions

### **CQ-11**: Is a different surveillance method according to causative gene for Lynch syndrome-associated tumors recommended?

A different surveillance method according to causative gene for Lynch syndrome-associated tumors is weakly recommended (Recommendation 2/Evidence level C).

## Comments

Patients with *MLH1* or *MSH2* variants have a higher risk of colorectal cancer compared to patients with *MSH6* or *PMS2* variants. Meanwhile, the risk of colorectal cancer is virtually equivalent between patients with *MLH1* variants and those with *MSH2* variants [13, 236]. Many studies suggest that patients with *MSH2* variants are at increased risk of Lynch syndrome-associated tumors other than colorectal cancer, particularly urinary tract cancer [252]. The risk of colorectal cancer development is lower in patients with *MSH6* variants than in those with *MLH1* or *MSH2* variants, but the risk of endometrial cancer development in patients with *MSH6* variants is equivalent to or higher than that in those with *MLH1* or *MSH2* variants. Patients with *MSH6* variants have an older age of onset for colorectal cancer (8–9 years) and endometrial cancer (3.9–5.7 years) compared to those with *MLH1* or *MSH2* variants [253, 254]. Although studies on *PMS2* variants are limited, the incidence of Lynch syndrome-associated tumors other than colorectal and endometrial cancers is low [255] (Table 20). Therefore, conducting surveillance for patients with Lynch syndrome according to causative gene, which considers the frequency and timing of Lynch syndrome-associated tumor development, is ideal.

**Table 20:** Lifetime risk of developing Lynch syndrome-associated tumors according to causative gene (up to the age of 70 years)

	*MLH1*	*MSH2*	*MSH6*	*PMS2*	General population (%)
Colorectal cancer	46–49%	43–52%	15–44%	12–20%	4.50
Endometrial cancer	43–57%	21–57%	17–46%	0–15%	2.70
Gastric cancer	5–7%	0.2–16%	0–5%	–	< 1
Ovarian cancer	5–20%	10–38%	1–11%	–	1.30
Renal pelvic/ureteral cancer	0.2–5%	2–18%	0.7–7%	–	< 1

It is generally advised that colonoscopy surveillance is started at the age of 20–25 years. If colorectal cancer was diagnosed before the age of 25 years in the family, then this should be started 2–5 years earlier than this age, but for *MSH6* variants, starting at the age of 30 years or 10 years earlier than the youngest age of onset in the family should be considered [13, 236]. There are insufficient data on *PMS2* variants, but starting at the age of 35 years should be considered [236]. However, the risk of development of Lynch syndrome-associated tumors for each causative gene has not yet been fully assessed in the Japanese population.

## **CQ-12**: Is surveillance for gynecologic cancers recommended in individuals with Lynch syndrome?

Surveillance for gynecologic cancer is weakly recommended in individuals with Lynch syndrome (Recommendation 2/Evidence level C).

## Comments

Lynch syndrome-associated gynecologic cancers include endometrial cancer and ovarian cancer. There is minimal evidence whether surveillance reduces mortality rate of endometrial cancer among pathogenic variant carriers of MMR genes. However, endometrial biopsy has a high sensitivity and specificity as a surveillance. There is minimal evidence for testing intervals of endometrial biopsy, but annual surveillance should be considered [256–261]. Endometrial cytology is not generally an alternative to endometrial biopsy because the former does not have a high accuracy rate of diagnosis although it may be considered at the discretion of the treating physician because it is less invasive at the time of examination compared with endometrial biopsy. Endometrial cancer surveillance by endometrial thickness using transvaginal ultrasonography has not shown sensitivity and specificity. Surveillance with transvaginal ultrasonography is not recommended [257–262], particularly in premenopausal women, because endometrial thickness varies widely depending on the menstrual cycle. In the case of abnormal genital bleeding, which is the main subjective symptom, it is also important to raise awareness, such as recommending gynecological consultations.

Although no effective surveillance method or interval is generally recommended for ovarian cancer, only transvaginal ultrasonography and serum CA-125 may be considered as physician’s choice. However, care must be taken against “interval cancer,” in which a previous consultation resulted in negative results but where subjective symptoms were observed and cancer is discovered prior to the next scheduled consultation. Although the initial subjective symptom is mild, it is important to raise awareness, such as recommending gynecology consultations, due to the possibility of an increased tumor size when symptoms such as lower abdominal pain, abdominal distension, abdominal circumference increase, feeding difficulty, urinary frequency, and urinary urgency were identified.

A recent prospective study of Lynch syndrome reported a 10-year survival rate of 98% for endometrial cancer and 89% for ovarian cancer when surveillance for gynecologic cancer was performed. Whether this result is due to the effectiveness of surveillance or the low malignancy of gynecologic cancers that develop in individuals with Lynch syndrome is currently unknown.

## **CQ-13**: Is risk-reducing surgery recommended for gynecologic cancers in individuals with Lynch syndrome?

Careful management is required to apply risk-reducing surgery for female individuals with Lynch syndrome, which is recommended overseas, under the medical care system in Japan. Risk-reducing surgery should be considered in women with Lynch syndrome after sufficiently examining the circumstances such as comorbidity and desire for raising children (Recommendation None/Evidence level C).

## Comments

Lynch syndrome-associated gynecologic cancers include endometrial cancer (uterine cancer) and ovarian cancer, each of which must be considered as another organ.

International guidelines and reports recommend risk-reducing surgery for women with Lynch syndrome who do not have gynecologic cancers, including cost-effectiveness considerations [13, 126, 237, 256, 258, 263]. This provides an opportunity, particularly before surgery for colorectal cancer, to consider performing risk-reducing surgery for gynecologic cancer.

Total hysterectomy is an option that should be considered as a risk-reducing surgery because it can prevent the development of endometrial cancer but has not shown to reduce mortality rate [13, 126, 237, 256, 258, 264, 265]. Endometrial biopsy may be considered instead of performing total hysterectomy for surveillance of endometrial cancer (CQ12).

No effective surveillance method has been proposed for ovarian cancer associated with Lynch syndrome. Therefore, risk-reducing salpingo-oophorectomy (RRSO) is an option to consider in a similar manner as the risk-reducing methods for hereditary breast and ovarian cancer syndrome [13, 126, 237, 256, 258, 264, 265]. RRSO reduces the onset of ovarian cancer in women with Lynch syndrome but has not been shown to reduce mortality rate of ovarian cancer [13, 126, 237, 256, 258, 264, 265].

The risk and age of onset of endometrial and ovarian cancers differ according to the type of MMR gene retained. Currently, there are no data regarding the relationship between the age of onset of Lynch syndrome and the incidence of endometrial and ovarian cancers in Japan, but the risk-reducing surgery can be individualized according to the desire to raise children, the presence of comorbidities including systemic diseases and/or Lynch syndrome-associated tumors such as colorectal cancer, and the type of causative MMR gene. However, specific methods have not yet been standardized. The age of individuals undergoing RRSO also affects some menopausal symptoms in the form of ovarian deficiency symptoms, changes in sexual activity, and lipid profile/bone metabolism. For this reason, the implementation of RRSO also requires the engagement of female health care professionals. Hormone replacement therapy may also be helpful in promoting health after RRSO in women without breast cancer history. Generally, it is recommended to perform risk-reducing surgery after the age of 35–40 years [126, 235–237, 256].

A recent prospective study of patients with Lynch syndrome reported a 10-year survival rate of 98% for endometrial cancer and 89% for ovarian cancer when surveillance for gynecologic cancer was performed [254]. The effectiveness of this method must also be verified in Japan to evaluate the effectiveness of risk-reducing surgery for gynecological cancer.

In conclusion, implementation of risk-reducing surgery for women with Lynch syndrome, which is recommended in other countries, must be sufficiently examined in advance under the approval of the institutional ethics review board and medical care system of Japan.

## **CQ-14**: Is universal MSI and IHC screening recommended in the diagnosis of Lynch syndrome?

Universal MSI and IHC screening is weakly recommended in the diagnosis of Lynch syndrome (Recommendation 2/Evidence level C).

## Comments

Universal screening is a method that involves MSI testing and IHC for mismatch repair proteins for all patients with colorectal or endometrial cancers (or those aged < 70 years), regardless of age or family history. Universal screening can be used to identify patients with Lynch syndrome with higher sensitivity compared to screening using age and family history. Among patients with Lynch syndrome identified by universal screening, 12–28% failed to meet the revised Bethesda guidelines [6, 9, 219, 220, 234, 266]. Universal screening is also useful in terms of avoiding efforts related to detailed interviews of family history and is also recommended by the international guidelines in terms of sensitivity and cost-effectiveness [13, 126, 235–238].

However, the reported incidence of Lynch syndrome in among patients with colorectal cancer based on universal screening was 2.4–3.7% [8, 9] overseas and 0.7% in Japan [10], and thus, the incidence of Lynch syndrome may be lower in Japan than that overseas. Moreover, the number of relatives who underwent diagnoses per proband was 3.6 [267], but there are limited data in Japan. Furthermore, the incidence of colorectal cancer among seniors in Japan has been increasing, the risk of Lynch syndrome in newly diagnosed colorectal cancer in a patient aged > 70 years is extremely low [10], and there is no established method of assessment for universal screening in Japan.

## **CQ-15**: Is genetic testing recommended for relatives in the family of Lynch syndrome?

Genetic testing for relatives in the family of Lynch syndrome is strongly recommended (Recommendation 1/Evidence level B).

## Comments

If a family member is diagnosed with Lynch syndrome, there is a high probability that their relatives will be variant carriers. The health risks can be reduced for relatives identified as variant carriers through various means, such as through surveillance. Moreover, family members who are found not to carry pathogenic variants can cease unnecessary screening. Therefore, it is strongly recommended that relatives be considered for genetic testing if there is a known pathogenic variant of Lynch syndrome in the family.

It is more efficient to test first-degree relatives and then second-degree relatives, respectively. The timing for genetic testing should be after the age of 18 years, unless there is a family history of cancer onset at a younger age (teenagers, 20 s) [268]. Undergoing genetic testing is at an individual’s own discretion.

The attending physician should, in principle, provide prior explanation and confirm consent for genetic testing with the objective of diagnosing patients who have already developed cancer [269].

In cases where relatives have not yet developed cancer, it is necessary to provide genetic counseling before and after genetic testing. To facilitate independent selections, it is important to provide not only information but also psychological and social support for patients and relatives; therefore, physicians with extensive clinical experience on Lynch syndrome and individuals familiar with genetic counseling should ideally cooperate with each other and implement these actions through team-based medical care [269].

## **CQ-16**: Is extended surgery (e.g., subtotal or total colectomy) recommended for newly diagnosed colorectal cancer in patients with Lynch syndrome?

Extended surgery is weakly recommended as an operative procedure for newly diagnosed colorectal cancer in patients with Lynch syndrome (Recommendation 2/Evidence level C).

## Comments

Studies on operative procedures for Lynch syndrome primarily involve retrospective observational studies, which compare subtotal and total colectomy as extended surgical procedures to segmental resection for sporadic colorectal cancer. Meta-analyses showed that metachronous colorectal cancer developed in 22.4–22.8% of patients underwent segmental resection and 4.7–6.8% of those underwent extended surgery. Segmental resection significantly increases the risk of developing metachronous colorectal cancer [270, 271]. For these reasons, extended surgery as a surgical procedure for newly diagnosed colorectal cancer in patients with Lynch syndrome is recommended with the objective of securely reducing the risk of metachronous colorectal cancer development [236]. Meanwhile, a study indicated that there were no differences in mortality rate between the two groups (relative risk of segmental resection, 1.65 [95% CI 0.90–3.02)] [271], but there has been insufficient discussion on this, and data relating to surgical procedures for newly diagnosed colorectal cancer in Japan are minimal [66, 272].

Although approximately 15% of newly diagnosed colorectal cancers in patients with Lynch syndrome are rectal cancers, most cases of metachronous cancers in patients undergoing proctectomy are right-sided colon cancers. A retrospective observational study showed that the cumulative incidence of multiple metachronous colorectal cancers detected by endoscopic surveillance at mean intervals of 14 months is 19% in 10 years, 47% in 20 years, and 69% in 30 years [273]. There are limited data on whether total proctocolectomy should be selected in newly diagnosed rectal cancer.

There is also no consensus on whether to perform prophylactic proctocolectomy for patient with MMR variants who are not yet affected by colorectal cancer. The lifetime risk of developing colorectal cancer in patients with Lynch syndrome is 54–74% in men and 30–52% in women, and there are a fair number of patients with MMR variants who did not develop colorectal cancer throughout their lives. We cannot uniformly advise prophylactic proctocolectomy as was the case for FAP.

Therefore, it is advisable to explain the risk of metachronous colorectal cancer, need for surveillance and its limitations, significance of prophylactic resection, postoperative quality of life, and status of comorbidities to patient with MMR variants. They should then be given the option to decide for themselves.

## **CQ-17**: Is adjuvant chemotherapy recommended for colorectal cancers in patients with Lynch syndrome?

Adjuvant chemotherapy is strongly recommended for Stage III colorectal cancer in patients with Lynch syndrome (Recommendation 1/Evidence level C).

## Comments

Because there is little evidence of chemotherapy specific to colorectal cancer in patients with Lynch syndrome, chemotherapy is often considered according to that for sporadic MSI-H colorectal cancers. However, the known differences between colorectal cancer in patients with Lynch syndrome and those with sporadic MSI-H colorectal cancer, such as the incidence of *BRAF* V600E variants or methylation status, should be recognized. Indeed, postoperative 5-fluorouracil (FU)-based adjuvant chemotherapy is ineffective in patients with sporadic MSI-H colorectal cancer but is effective in patients with MSI-H colorectal cancer aged < 50 years with suspected Lynch syndrome [274], suggesting that colorectal cancer in patients with Lynch syndrome should be considered differently from sporadic MSI-H colorectal cancers. There are almost no useful data on postoperative adjuvant chemotherapy for sporadic MSI-H rectal cancer or Lynch syndrome-associated rectal cancer.

Meta-analyses on the MSI status and efficacy of postoperative adjuvant chemotherapy including 5-FU in cases with stage II/III sporadic colorectal cancer showed that MSI-H colorectal cancer had a better prognosis than MSS colorectal cancer but that postoperative adjuvant chemotherapy did not improve the survival or recurrence-free survival in patients with MSI-H colorectal cancer [275, 276]. However, the National Surgical Adjuvant Breast and Bowel Project (NSABP)-C07 trial and the Multicenter International Study of Oxaliplatin/5-Fluorouracil/Leucovorin in the Adjuvant Treatment of Colon Cancer (MOSAIC) trial showed that oxaliplatin had an additive effect in postoperative adjuvant therapy for both MSI-H and MSS colonic cancers [277]. Therefore, presently, it is not recommended to determine whether stage III colonic cancer is an indication for postoperative adjuvant chemotherapy according to the MSI status. The usefulness of postoperative adjuvant chemotherapy has not been established for stage II colorectal cancer, and it is thought to be less useful, particularly in MSI-H cancers, because these cancers have favorable prognoses.

## **CQ-18**: Is chemotherapy recommended for advanced/recurrent colorectal cancer in patients with Lynch syndrome?

Chemotherapy for advanced or recurrent colorectal cancer in patients with Lynch syndrome is strongly recommended (Recommendation 1/Evidence level C).

## Comments

The incidence of MSI-H has been shown to be lower in stage IV than in stage II/III sporadic colorectal cancers [278, 279]. Chemotherapy specific to metastatic colorectal cancers associated with Lynch syndrome or colorectal cancers showing MSI-H has not yet been clearly investigated, and no conclusion has been reached. Therefore, regimens generally selected for sporadic colorectal cancers could be also indicated for these cancers. The response rate to irinotecan as a second-line treatment in cases with acquired resistance to 5- FU was reported to be significantly higher in MSI-H cancers than in other sporadic colorectal cancers [280]. The CALGB/SWOG80405 trial was a phase III trial that compared the efficacy of chemotherapies plus either cetuximab or bevacizumab in first-line treatment for advanced recurrent colorectal cancer, and the results did not show significant differences in OS between the two. A comprehensive genetic analysis of this trial showed that combinations with bevacizumab instead of cetuximab significantly extended the survival time in colorectal cancer with MSI-H, while neither combined therapy showed significant differences in colorectal cancer with MSS [281].This may also be due to the effect of the primary site, so it is necessary to wait and judge the results of future clinical studies regarding the efficacy of MSI-H colorectal cancer and molecular targeted drugs.

## **CQ-19**: Are immune checkpoint inhibitors recommended for advanced and recurrent colorectal cancers in patients with Lynch syndrome?

Immune checkpoint inhibitor therapies for advanced or recurrent colorectal cancer in patients with Lynch syndrome are strongly recommended (Recommendation 1/Evidence level B).

## Comments

A phase II trial (KEYNOTE-016) analyzing the efficacy of pembrolizumab in MSI-H/dMMR* colorectal cancer, MSI-H/dMMR solid cancers other than colorectal cancer, and MSS colorectal cancer after third-line treatment showed response rates of 40, 71, and 0%, respectively, with effectiveness of anti-PD-1 antibodies against MSI-H/dMMR solid cancers [282]. Follow-up studies on 86 patients with MSI-H/dMMR solid cancer extended to 12 carcinoma types showed that the response rate was 53% (52% for colorectal cancer, 54% for non-colorectal cancer). Of these, the response rates for Lynch syndrome-associated and non-associated tumors were 46 and 59%, respectively, and thus showed comparable results [283].

A phase II trial (CheckMate 142) that investigated the efficacy of either nivolumab alone or nivolumab + ipilimumab in MSI-H/dMMR colorectal cancers after third-line therapy showed that the response rates were 31 and 55%, respectively. The incidence of grade 3/4 treatment-related adverse events was 20 and 32%, respectively [284, 285]. Clinical data revealed that 36 and 29% of Lynch syndrome-associated tumors were included, respectively, with response rates of 33 and 71% being comparable to the overall results.

It is also known that approximately 90% of patients with Lynch syndrome colorectal cancers present with MSI-H/dMMR. Therefore, we recommend treatment with immune checkpoint inhibitors for advanced and recurrent colorectal cancer in patients with Lynch syndrome.

*dMMR: loss of MMR-protein expression by IHC.

## **CQ-20**: Are lifestyle remedies recommended to prevent carcinogenesis in patients with Lynch syndrome?

It is strongly recommended to implement lifestyle remedies to prevent carcinogenesis in patients with Lynch syndrome (Recommendation 1/Evidence level C).

## Comments

Several risk factors related to diet, alcohol consumption, and exercise have been shown to reduce the risk of colorectal cancer in patients with Lynch syndrome, in addition to maintaining adequate body weight and smoking cessation.

High body mass index (BMI) has been shown to increase the risk of developing adenomas or colorectal cancers, so we recommend maintaining within the mean body weight range [235]. A prospective cohort study has shown that in particular men with BMI > 25 kg/m2 were at higher risk of colorectal cancer [286]. A randomized controlled trial has also reported that obesity increased the risk of colorectal cancer by a factor of 3.72 when the *MLH1* variant is present but that no such increases in risk were present when aspirin was used or when *MSH2* or *MSH6* variants were present [287]. A cross-sectional study and systematic reviews recommended the cessation of smoking as results showed that smoking increased the risk of colorectal cancer [235, 288, 289].

Current smoking as opposed to past smoking in particular was shown to increase the risk of colorectal adenomas [290]. Moreover, a retrospective cohort study showed that the consumption of multivitamin and calcium supplements reduced the risk of colorectal cancer [291], case–control studies and a retrospective cohort study or prospective observational studies showed that increased fruit intake decreased the risk of colorectal cancer [292, 293], retrospective cohort studies or cross-sectional studies showed increases in colorectal cancer risk due to alcohol consumption and younger age of onset [293–295], and a retrospective cohort study suggested that increased physical activity reduced the risk of colorectal cancer [296].

## **CQ-21**: Is chemoprevention (aspirin) recommended to prevent carcinogenesis in patients with Lynch syndrome?

In patients with lynch syndrome, not to use aspirin to prevent carcinogenesis is weakly recommended at this time (Recommendation 2/Evidence level B).

## Comments

The Colorectal Adenoma/Carcinoma Prevention Program 2 (CAPP2) was the first double-blind randomized controlled trial to evaluate the preventative effects of aspirin (600 mg/day) against patients with Lynch syndrome-associated tumors and colorectal adenomas. There were no significant differences in the prevention of incidence of colorectal adenoma at the end of 4-year intervention, but in the group where aspirin was administered more than 2 years, long-term follow-up observations showed that its significant preventative effects on the incidence of colorectal cancer and Lynch syndrome-associated tumors [297]. Further CAPP2 analyses demonstrated that obese patients with Lynch syndrome had an increased risk of colorectal cancer by 7% for every increase of 1 kg/m2, but this risk increase was eliminated by aspirin [287].

Of note, long-term aspirin use increases the risk of gastrointestinal disorders. Moreover, it has been reported that the suppressive effects of aspirin on colorectal cancer risk may be weight-dependent in the general population. Thus, the disadvantage of aspirin may outweigh the benefit when patients given fixed dose without considering optimal body weight [298]. For this reason, we recommend not to use of aspirin as a chemopreventive agent against carcinogenesis at this time.

The optimal dose and duration of aspirin for patients with Lynch syndrome, including low-dose aspirin (100 mg/day), is currently being investigated.

## **CQ-22**: Is colonoscopy surveillance recommended in individuals with Lynch syndrome?

Colonoscopy surveillance in individuals with Lynch syndrome is strongly recommended (Recommendation 1/Evidence level B).

## Comments

Individuals with Lynch syndrome have a high risk of developing colorectal cancer, including those with remaining large intestine after surgery for colorectal cancer, and regular (repeated) and lifelong endoscopic surveillance is required with the aim of any resecting precancerous adenomas and early detection of colorectal cancer [13, 236]. Several studies have recommended that surveillance be started at the age of 20–25 years [13, 236].

Regarding the intervals of colonoscopy surveillance should be conducted, a prospective study by Järvinen et al. reported that colonoscopy surveillance at 3-year intervals decreased the mortality of colorectal cancer by 65% [299]. However, observational studies have confirmed the development of advanced colorectal cancer in a 3-year period in colonoscopy surveillance, so several studies recommend annual surveillance [126, 300, 301]. However, some studies showed no significant difference in the incidence or stage of colorectal cancers when comparing surveillance intervals between 1 and 3 years [302, 303], and no consensus has been available. However, these overseas reports on postoperative colonoscopy surveillance have few descriptions on bowel preparation, cecum intubation rate, adenoma detection rate, observation time, etc., which could serve as indicators for colonoscopy quality assurance. The higher rate of interval cancer found rather than stage I should also be considered for these reports. Future studies on surveillance in high-quality colonoscopies are anticipated.

In patients with Lynch syndrome, colorectal adenomas are characterized by a small number (generally within dozens), development at a young age (< 40 years), large size, villous features, MSI-H, high-grade atypia even if smaller than ordinary adenomas, and shorter time to malignant transformation [300, 304–307]. Several pathways have also been postulated for carcinogenic mechanisms in Lynch syndrome associated colorectal cancer [307], but they are difficult to distinguish at the time of colonoscopy observation, so neoplastic lesions are subject to aggressive endoscopic removal when detected.

## Figures and Tables

**Fig. 29:**
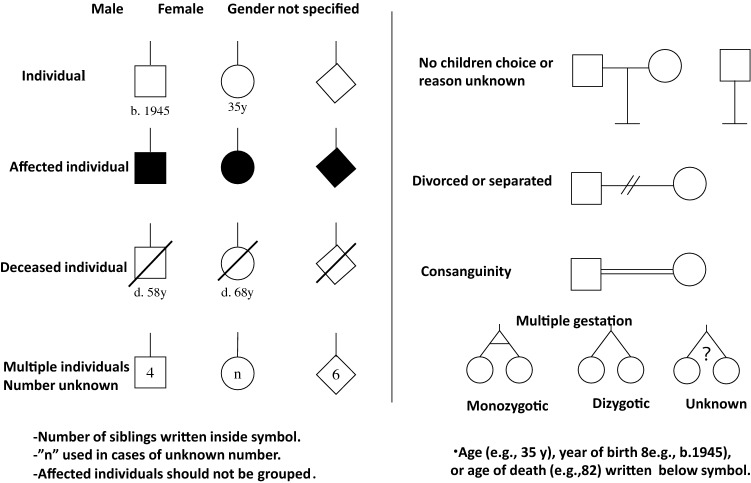
Symbols for family pedigrees

**Fig. 30:**
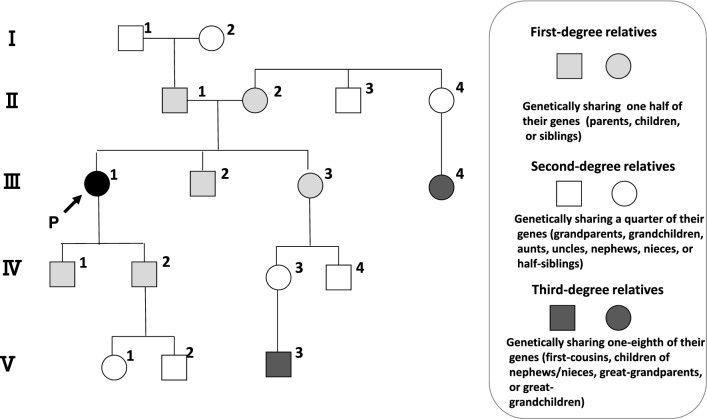
The first-, second-, or third-degree relatives of the proband

**Fig. 31:**
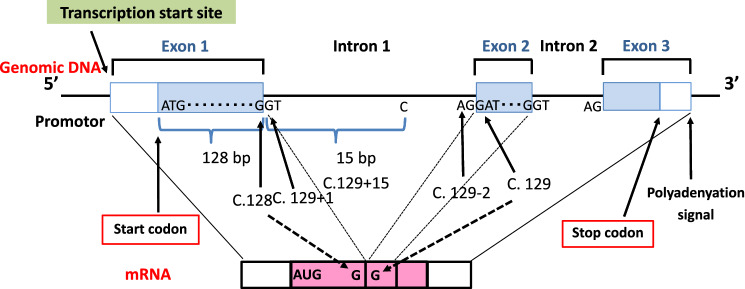
The gene structure and variant nomenclature

## Appendix

### I: Principles for writing and reading pedigrees

1. Points for taking family history (Appendix Fig. 29)

- Information on at least three generations should be obtained.
- It should be checked whether there are any consanguineous marriages (e.g., cousin marriages).
- Not only the number of affected individuals but also the number of unaffected individuals among siblings should be checked.
- Date of taking family history, name of the person providing the information, and name of the person taking the family history should be described in the pedigree.
- Maternal and paternal pedigrees should be separately evaluated.

2. Outline on how to draw pedigrees (Appendix Fig. 30)

- The proband (the affected individual leading to the detection of the affected family) should be indicated by P↗
- Clients should be indicated by↗
- If possible, the husband (male partner) should be listed to the left of the wife (female partner).
- Siblings should be listed from left to right in order of birth.
- The generation number should be indicated in Roman numerals on the left side.
- Individual numbers should be given in Arabic numerals in order from left to right along generation lines.
- Necessary clinical information, such as the age at onset (age at diagnosis), affected site (left or right in the case of bilateral disease), course of treatment, surgical procedure, and pathological diagnosis, should be described.

Symbols generally used to draw pedigrees are shown below.

### II: Method for describing genomic variants

- The description method proposed by the Human Genome Variation Society (http://varnomen.hgvs.org/) is generally used to describe genomic changes. Usually, information on reference sequences, their location, and any changes should be given in that order.

1. Symbols for reference sequences

Genomic reference sequence: g.

Coding DNA reference sequence^#^: c.

RNA reference sequence: r.

Protein reference sequence: p.

^#^Coding DNA sequence is a DNA sequence between the start and stop codons that serves as a template for the synthesis of mRNA, which is translated into protein.

2. Locations of variants
(1) Changes at the genomic DNA level should be indicated by “g.,” and the first nucleotide of the reference genome sequence should be numbered 1.
(2) Changes at the coding DNA level should be indicated by “c.,” and the A of the start codon ATG (translation start point) should be numbered 1 (in Appendix Fig. 31, the last nucleotide of exon 1 is the 128th nucleotide from the A of the start codon ATG and should be indicated as c. 128). Because coding DNA sequences are translated into proteins and contain no introns, when a nucleotide position in an intron is shown, the nucleotide number counted from an adjacent exon should be indicated using “+” or “−”. For example, in Appendix Fig. 31, the 15th nucleotide from the start of intron 1 should be indicated as “c. 128 + 15”, and the second nucleotide upstream of the start of exon 2 (c. 129) should be indicated as “c. 129 − 2”.
(3) Changes at the RNA level should be indicated by “r,” in accordance with the method for describing the changes at DNA level.
(4) Changes at the protein level should be indicated by “p.,” and the translation initiation methionine should be numbered 1. Both three-letter and one-letter amino acid codes can be used.
3. Types of changes and their descriptions
(1) Changes at the DNA level should be described as follows, Substitution: >, deletion: del, insertion: ins, deletion-insertion: delins, duplication: dup, inversion: inv, and conversion: con.
(2) For changes at the protein level, “>” is not used in the case of substitution, but the original and changed amino acids are shown before and after the amino acid position (number), respectively. Other changes, such as deletion (del), insertion (ins), deletion-insertion (delins), duplication (dup), inversion (inv), and conversion (con), are described in a similar manner to those at the DNA level.

In general, changes are commonly described at the coding DNA (c.) or protein (p.) level.

Examples are provided below.

#### Example 1) Missense variant

- c. 146 T > A (p. Val49Glu)

The T nucleotide at the 146th position from the A of the start codon ATG is substituted with an A. This is associated with change of the 49th amino acid from valine (Val) to glutamic acid (Glu).

#### Example 2) Nonsense variant

- c. 184C > T (p. Glu62Ter or p. Glu62^*^)

The C nucleotide at the 184th position is substituted with a T. This is associated with the 62nd codon becoming a stop codon (* indicates a stop codon.), resulting in a stop of protein biosynthesis.

#### Example 3) Duplication and associated frameshift variant

- c. 175dupA (p. Ile59Asnfs^*^20)

The A nucleotide at the 175th position is duplicated, and the 176th nucleotide becomes A with the shift in the codon reading frame (this shift of the reading frame is called frame shift and designated as “fs”). This is associated with change of the 59th amino acid from isoleucine (Ile) to asparagine (Asn) and, furthermore, with the 20th codon from this site becoming a stop codon (fs*20), resulting in the termination of protein biosynthesis.

#### Example 4) Deletion and its associated frame-shift variant

- c. 3927_3931delAAAGA (p. Glu1309Aspfs^*^4)

The nucleotides the 3927th to 3931th position, AAAGA, are deleted. This is associated with change of the 1309th amino acid from glutamic acid (Glu) to aspartic acid (Asp) and the 4th codon from this site becoming a stop codon (fs* 4).

#### Example 5) Variant in an intron

- c. 792 + 1G > A

The G nucleotide at the first position following the last (792nd) of the exon is substituted with an A. This is speculated to be associated with abnormal splicing.

#### Example 6) Exon deletion

- c. 458-?_627 + ?del

At least one exon (DNA sequence from c. 458 to c. 627) is deleted (unknown nucleotides in the deleted intron region are indicated by “?”).

In addition, to assess whether the variant obtained causes disease or not, registration of the variant in databases, such as InSiGHT (http://insight-group.org/variants/database/) and ClinVar (http://www.ncbi.nlm.nih.gov/clinvar/), should be checked, and a comprehensive assessment based on the results is sometimes required.

It cannot be necessarily said that variants listed in databases cause disease, and careful management is required. Variants are usually classified into five categories according to whether they can be associated with disease (Details I 2-2: Genetic testing Table 6 [p. 20]).
